# Insect Lipids as Novel Source for Future Applications: Chemical Composition and Industry Applications—A Comprehensive Review

**DOI:** 10.1002/fsn3.70553

**Published:** 2025-07-20

**Authors:** Shahida Anusha Siddiqui, Asma Zeiri, Mohd Asif Shah

**Affiliations:** ^1^ Independent Researcher Germany; ^2^ Independent Researcher Italy; ^3^ Department of Economics Kardan University Kabul Afghanistan; ^4^ Division of Research and Development Lovely Professional University Phagwara Punjab India; ^5^ University Centre for Research & Development Chandigarh University Gharuan, Mohali Punjab India

**Keywords:** biodiesel, edible insects, energy, feed, food, lipid, oil

## Abstract

Nutritional values of some insects are correlated with species, developmental stages, temperature, reproduction, flight, migration, and their diet. Insect oil may be utilized as an ingredient in feed and food, in the cosmetic industry, or as biofuel. Lipid content is composed mainly of fatty acids (FAs) of nutritional importance. Insects' fat body (FB) is crucial for lipid synthesis, accumulation, and hydrolysis. Triacylglycerol (TAG) accumulation occurs mainly in adipocytes. The major transported lipid is diacylglycerol, which is carried by high‐density lipoprotein (lipophorin [Lpp]) from the gut into a trip to tissues for storage and utilization. About 1900 insect species, mainly from the Coleoptera, Lepidoptera, Hymenoptera, Orthoptera, Hemiptera, Isoptera, Odonata, and Diptera orders, were recorded to be consumed by humans in the world, particularly in Asia, Latin America, and Africa. Different methodologies used to extract lipids are currently applied to insects, such as the aqueous extraction, the Soxhlet method, the Folch method, the Matyash method, the supercritical fluid extraction method, the Aqueous Enzymatic method, and the acid fermentation method. This review shows that the Coleoptera, Lepidoptera, and Isoptera orders contain the higher amount of fat/oil at the same level as palm and palm kernel oil. In the larval stage, *Samia ricini* had the higher fat content in terms of crude fat, followed by *Rhynchophorus phoenicis*, *Rhynchophorus ferrugineus*, commercial palm oil, *Aspongopus viduatus* (adult), *Oryctes owariensis* (Larvae), and *Macrotermes nigeriensis* (Adult) and palm kernel oil. Understanding lipid metabolism, composition, and physiological implications is fundamental for insect mass rearing and harvesting according to the final use target. This review aimed to contribute to knowledge about insects as sources of fats and FAs. Lipids from insects could play an important role as a source of energy if incorporated into human diets and make a valuable integration into feed and food as long as they are safe, legal, and accepted.

## Graphical abstract

Fat body (FB) acts as a major metabolic center important for the regulation of a variety of physiological processes such as embryogenesis, reproduction, flight, overwintering, protection, and communication. Lipid amounts and fatty acid (FA) composition vary with species, stage of life, and their diet.

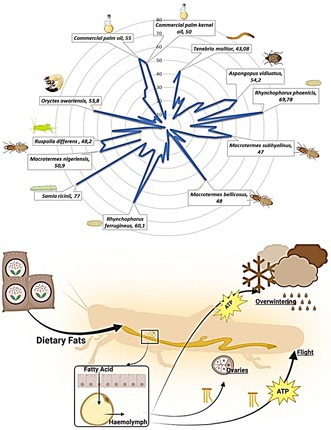

**Abbreviations**
ACBP: acyl‐CoA‐binding protein
ACC: acetyl‐CoA‐ carboxylase
ACS: acyl‐CoA synthetase
Adipo: adiponectin
AdipoR: adiponectin receptor
AGPAT: 1‐acylglycerol‐3‐phosphate *O*‐acyltransferase
AI: atherogenic index
AKH: adipokinetic hormone
AKHR: adipokinetic hormone receptor
AKT: serine‐threonine‐protein kinase
AMPK: AMP‐activated protein kinase
apo: apolipoprotein
APOE: apolipoprotein E
ApoLpI: apolipophorin‐I
ApoLpII: apolipophorin‐II
ApoLpII/I: apolipophorin‐II/I
ApoLpIII: apolipophorin‐III
ATGL: adipose triglyceride lipase
cAMP: response element‐binding protein
CHD: coronary heart disease
CSI: cholesterol index
CVD: cardiovascular disease
DAG: diacylglycerol
DGAT: diacylglycerol *O*‐acyltransferase
DH: diapause hormone
DHR: diapause hormone receptor
DILP: Drosophila insulin‐like peptide
EcR: ecdysone receptor
ER: endoplasmic reticulum
ERK: extracellular signal‐regulated protein kinase
FA: fatty acid
FABP: fatty acid‐binding protein
FA‐CoA: fatty acyl‐CoA
FAS: fatty acid synthase
FATP: fatty acid transport protein
FB: fat body
G3P: glycerol‐3‐phosphate
GABA: gamma‐aminobutyric acid
GPAT: glycerol‐3‐phosphate *O*‐acyltransferase
GPCR: G protein‐coupled receptor
HDL: high‐density lipoprotein
HDLp: high‐density lipophorin
HPGs: hypopharyngeal gland
IGF: insulin growth factor
ILP: insulin‐like peptide
InR: insulin receptor
IPC: insulin‐producing cell
JH: juvenile hormone
LD: lipid droplet
LDL: low‐density lipoprotein
LDLp: low‐density lipophorin
Lp: lipophorin
LpR: lipophorin receptor
LpR1: lipophorin receptor 1
LpR2: lipophorin receptor 2
LSD1: lipid storage droplet 1
LSD2: lipid storage droplet 2
LTP: lipid transfer particle
MAG: monoacylglycerol
MCPD: monochloropropandiol
MGAT: monoacylglycerol *O*‐acyltransferase
NPF: neuropeptide F
OA: octopamine
PA: phosphatidic acid
PAP: phosphatidic acid phosphatase
PKA: protein kinase A
PKC: protein kinase C
PLC: phospholipase C
SCP: sterol carrier protein
SFA: saturated fatty acid
TAG: triacylglycerol
TGL: triglyceride lipase
TI: thrombogenic index

## 1 Introduction

Thought insects might be the origin of some damage to the food and agriculture; they also can have a very good impact, and they can be consumed in different stages, such as eggs, larvae, pupae, and/or adults, in different regions of the world (Costa‐Neto and Dunkel 2016; dos Santos 2021).

**FIGURE 1:**
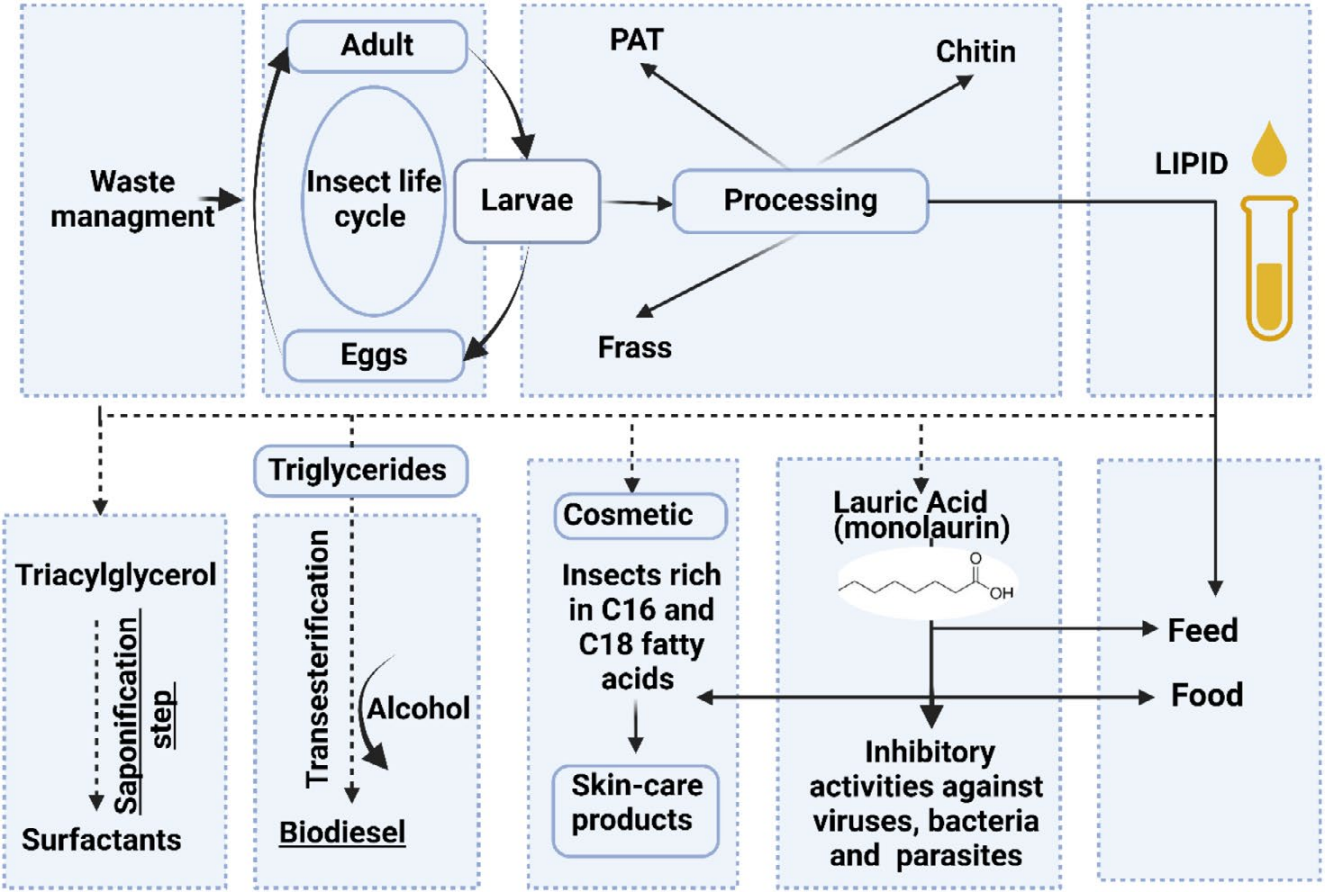
Recycling insects' fat for industrial uses.

**FIGURE 2:**
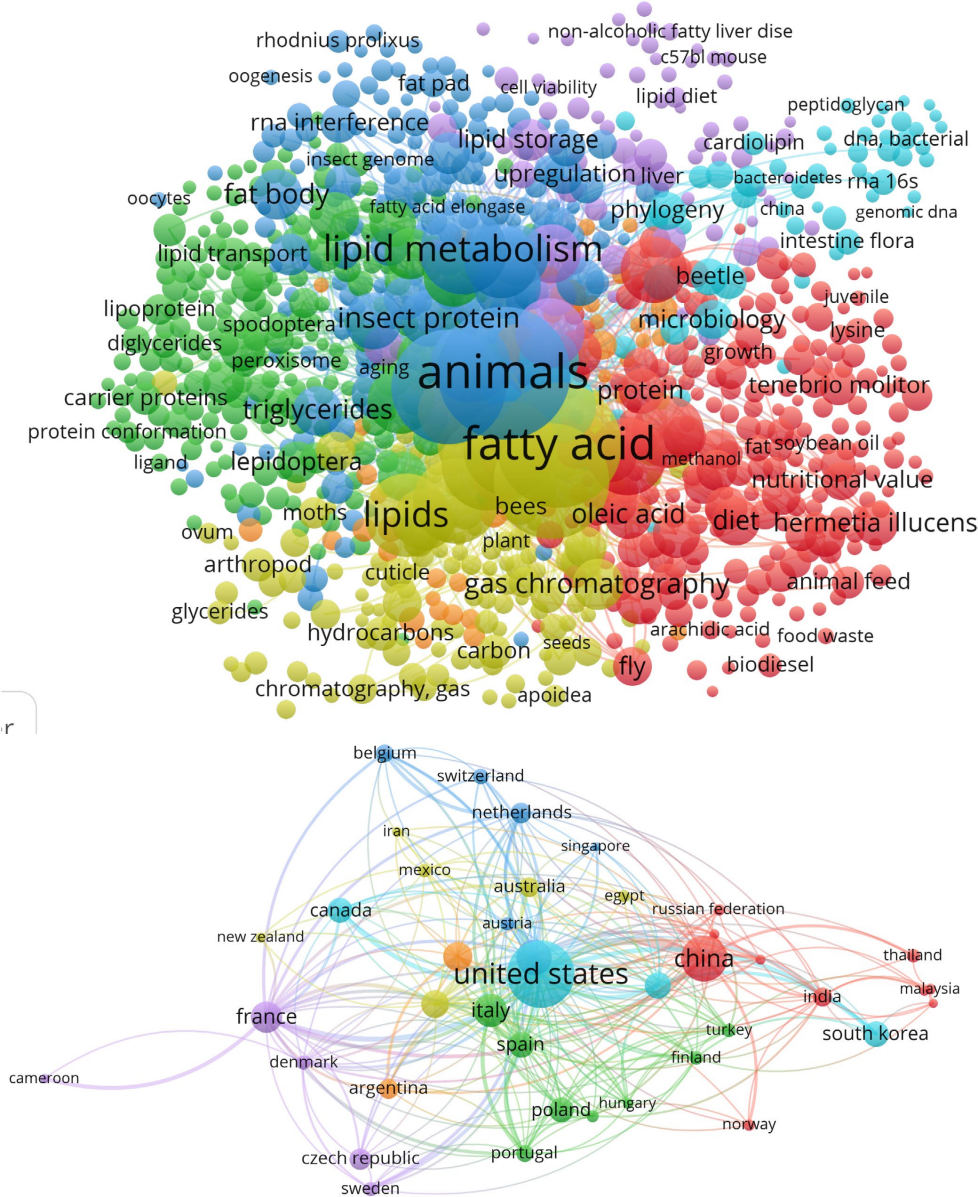
Bibliometric map on insects' fatty acid and lipid‐related bibliography and publications countries (keywords: Insects AND fatty AND acid AND lipid).

Nutritional values among species of insects are correlated to the stage of life and their diet. They can be a source of proteins, lipids, minerals, and vitamins. Lipids represent, beside the protein fraction, the second largest part of insects. Insect oil can substitute, as an ingredient, other fat sources in food or feed applications, in the cosmetic industry, or as biofuel (Figure 1). At an industrial scale, the use of insect lipid is still today poorly documented (Figure 2; Lorrette and Sanchez 2022). Lipid content is composed mainly of fatty acids (FAs) with great nutritional benefits (Nowak et al. 2016; Rumpold and Schlüter 2013a, 2013b; dos Santos 2021).

Lipids are implicated in different physiological processes such as diapause, development, metamorphosis, reproduction, embryogenesis, growth, and flight. Lipid reserve is a source for egg production, which is the reason why generally, the females' lipid contents are higher than males (Toprak and Musselman 2021; Wrońska et al. 2023; dos Santos 2021). During periods of food shortage, lipids enable the survival and overwintering of some insects (Wrońska et al. 2023). Triglyceride is the most predominant fraction in most insect species (Fruttero et al. 2017; Wrońska et al. 2023). In holometabolic insects, 95% of metamorphosis energy derives from the oxidation of FAs, which explains the increase in body lipid content during larval development (Palm and Rodenfels 2020; Wrońska et al. 2023). Migrating insects convert carbohydrates from the diet, during intensive lipogenesis, into lipids and then store them as triglycerides in the fat body (FB) (Hou et al. 2021; Wrońska et al. 2023).

Insect's lipids may be a result of feed or de novo synthesis (Tzompa‐Sosa and Fogliano 2017; dos Santos 2021). Insect lipids can be detected in many tissues such as flight muscles, midgut, and ovaries (Wrońska et al. 2023). However, the central tissue for fat storing and the center of lipid metabolism is the FB (Toprak et al. 2020; Wrońska et al. 2023). Many factors have an effect on the amount of lipids in insects (nutrition, sex, reproduction, flight and migration, temperature, developmental stages, etc.; Wrońska et al. 2023).

The FB of insects is the key place of lipid synthesis, accumulation, and hydrolysis. Triacylglycerol (TAG) accumulation occurs mainly in adipocytes, the main cells of the FB, in intracellular lipid droplets (LDs) (Skowronek et al. 2021; Wrońska et al. 2023). Under the control of the insect endocrine system, those physiological processes occur with the help of fatty acid synthase (FAS) and perilipins (Gondim et al. 2018; Toprak et al. 2020; Wrońska et al. 2023). The lipid transport system is very important in fat metabolism in insects. The lipophorin (Lpp), a high‐density lipoprotein, transports the diacylglycerol carried by reusable shuttles that pick up lipids from the gut for storage and use in different tissues (Canavoso et al. 2001).

During protein isolation, insect's lipids constitute the main produced component of insects (Yi et al. 2013; Tzompa‐Sosa et al. 2014). The extraction of fats and proteins from insects to be used as ingredients for food is an acceptable way to introduce them to the final consumer. Oleic, linolenic, palmitic, linoleic, and acids, for example, are considered healthy fractions of FAs (dos Santos 2021; Tzompa‐Sosa et al. 2014). Insects, in general, may have higher unsaturated FAs (dos Santos 2021; Rumpold and Schlüter 2013a, 2013b; Tzompa‐Sosa et al. 2014).

The extraction of insect's lipids is based on the diverse chemical compounds of lipids which are soluble in organic solvents. Extracted lipids, at room temperature, can be liquids called oils or solids called fats (dos Santos 2021).

Mariod (2013) targeted in his research *Aspongopus vidiuatus* (melon bug) and *Agonoscelis pubescens* (sorghum bug), insects of food and medicinal uses in Sudanese indigenous knowledge. The author investigated those species as oil, protein, and gelatin sources for both nutritional and industrial applications. *A. vidiuatus* and *A. pubescens* showed 45% and 60% oil with high percentages of oleic acid, linoleic acid, and palmitic acid. The insect oil also contained some traces of linolenic acid (Mariod 2013). The authors also measured the sterols and tocopherol in the two oils and found that β‐sitosterol is the main composite in all oils (60% of the total sterol).

Mariod (2013), also studied the behavior of the crude Sorghum bug oil during deep‐frying of par‐fried potatoes measuring its chemical, physical, and sensory parameters. Results showed the oil as suitable for deep‐frying of potatoes and that the oxidative stability of sunflower kernel oil can be improved if blended with melon bug oil. The tocopherol reacts with lipid radicals as chain‐breaking antioxidants content of foods stabilizing protecting food lipids against autoxidation and consequently increasing their storage life and their value. The amount insects' oil in tocopherols and sterols is lower than most edible oils; the sorghum bug oil was found to have higher amounts of tocopherol content comparing to melon bug oil. In the same study, authors tested the use of the bug oil as biodiesel transesterifying it with ethanol or methanol using the sulfuric acid.

Lipids from insects can contribute to combat human malnutrition by supplying energy and essential FAs, especially in developing countries (Tzompa‐Sosa et al. 2014). Lipid content from insects is quite rich in unsaturated C18 FAs like oleic acid (18:1 cis9), linoleic acid (18:2 cis9,12) and linolenic acid (18:3cis); it varies on a fresh weight basis to more than 30% (Tzompa‐Sosa et al. 2014). Rearing insects as a potentially interesting nutritious food source rich in protein, minerals, and vitamins has a low environmental impact with a future prospect of insect production business (Tzompa‐Sosa et al. 2014; A. Van Huis 2013). In the present, eating insects in most developed countries is still not largely accepted (Tzompa‐Sosa et al. 2014). About 1900 insect species are documented to be consumed as human food in the world, especially in Africa, Asia, and Latin America (A. Van Huis 2013; Tzompa‐Sosa et al. 2014). Jongema (2017) published a list of 1900 edible insect species worldwide. Globally, the major orders of edible insects are Coleoptera (beetles), Lepidoptera (caterpillars), Orthoptera (grasshoppers, locusts and crickets), Hymenoptera (Bees, ants and wasps), Hemiptera (leafhoppers, cicadas, planthoppers, true bugs and scale insects), Odonata (dragonflies), Isoptera (termites), and Diptera (flies) (Barennes et al. 2015). Some insects are consumed entirely at larval, pupal stages, or mature stage. Insects serve as a vital food source for many animals and humans. Their abundance and diversity make them an essential part of ecosystems worldwide. Insects' species number was estimated to be 5.5 million (Stork 2018). They play an essential ecological and economical role as decomposers like termites (Isoptera), promoting the reworking of soils and sediments and the cycling of nutrients, stimulating vegetal, animal, and microbial biodiversity (A. van Huis 2014). Globally, the number of beetle species is estimated to be approximately 1.5 million (Stork 2018). Coleoptera such as dung beetles' role in the ecosystem is crucial by mixing dung with soil to contribute to the growth of plants by providing seed dispersal and nutrient cycling (A. van Huis 2014). Many Lepidoptera species, especially butterflies, play an important role in pollination, supporting agriculture and biodiversity (Losey and Vaughan 2006).

The insect industry is a growing sector, and it is somehow restricted due to the various regulations out of date targeting the use of insect food and feed (Lahteenmaki‐Uutela et al. 2021; Lorrette and Sanchez 2022).

Currently, eight insect species are authorized for the production of processed animal proteins to include in feed for farmed animals within Europe: the house cricket *Acheta domesticus* , the tropical house cricket *Grillodes sigillatus*, the Jamaican field cricket *Gryllus assimilis* , the pest of the grain storages *Tenebrio molitor* , the lesser mealworm *Alphitobius diaperinus* , the black soldier fly (BSF) *Hermetia illucens*, the house fly *Musca domestica* , and the domestic silk moth *Bombyx mori* (Regulation 2017/893 (Annex X); Regulation (EU) 2021/1925).

The substrates, mainly of vegetal origin, on which those insects may be reared are strictly defined in the EU regulation (Lahteenmaki‐Uutela et al. 2021). Fat and oil from insects have much fewer restrictions compared to insect proteins.

Highly nutritional insect oils are suitable as food ingredients (Berezina 2017). Insects' solid fat may find potential applications in bakery. Delicato et al. (2020) envisaged the potential of bakery products that contain crude black soldier fly larvae (BSFL) fat at different percentages of incorporation to substitute butter for waffles, cookies, and cakes cooking. The replacement of butter at 25% was at the level of expectations.

Berezina (2017) reviewed insect lipid applications. The author considered also the potential utilization of insects oils coming from biofuels compared to some algal and plant biofuels and found that the time‐consuming “insect” source took much less time, followed by the plant source and consuming the algal source. The cetane number, the main intrinsic characteristic of the biofuel, was found to be higher in *T. molitor* and *H. illucens* than in canola oil and meets the European norm EN 14214 requirements (Zheng et al. 2013). Siow et al. (2021) demonstrated the effectiveness of the transesterification process, using a conventional base catalyst, to convert triglycerides from *T. molitor* oil into biodiesel. This finding was in accordance with (Lee et al. 2022) proving that *T. molitor* larvae extracted oil was easily converted to biodiesel by a thermal noncatalytic reaction.

Under Novel Food regulation (EU) No 2015/2283, insect food products may be marketed only when approved and authorized after a safety assessment by the European Food Safety Authority (EFSA). Thus, studies have proved that insect oil/fat is very similar to some plant oils; those requirements are also expected for insects lipid as a Novel Food product. Oil derived from insects might be subject to microbiological or chemical hazards such as fungi, bacteria, heavy metals, or mycotoxins. Those hazards have to be quantified and their safety has to be evaluated in order to obtain regulatory approval for products commercialization (EFSA 2015).

Alves et al. (2019), investigated *T. molitor* and *Pachymerus nucleorum* oil toxicity using rat models, using acute toxicity in a single oral administration and subacute toxicity in 28 consecutive daily administrations. Authors demonstrated that insects' oil has reduced cholesterol and glucose levels of the treated rats; besides, it did not cause any lethality, nor change hematological parameters, suggesting that in both acute and subacute toxicology experiments, *T. molitor* and *P. nucleorum* oil had low toxicity.

Various studies demonstrated insect‐derived fat and oil as potential substitutes in feed applications. Oil and fat extracted from insects, other than feed and food markets, may also have some promising applications in energy and therapeutic applications. Kierończyk et al. (2018), investigating the effects of replacing soybean oil with *T. molitor* and *Z. morio* oil on broiler performance, showed no impact on growth performance with the complete replacement of soybean oil. It instead affects broiler digestibility positively, improving FA profile and meat quality. The same results were demonstrated by other authors using *H. illucens* larvae oil for broiler chickens (Schiavone et al. 2018) and *T. molitor* and *H. illucens* fats on rabbits (Dabbou et al. 2020). The potential implication of *H. illucens* fat was reported by Hender et al. (2021). Previously, Henry et al. (2015) reported the use of domesticated silkworm pupa oil (Lepidoptera) as a fish oil substitute and its benefits, as it stimulates the appetite and the growth parameters in common carp.

Because of their chemical composition, insect fats can be used also in body care products and surfactants by a saponification step from triacylglycerol (Berezina 2017; Franco et al. 2022).

Several researches investigated lipids from insects of forensic, veterinary, and medical interest (Kaczmarek and Boguś 2021). Kim, Bang, et al. (2021) suggested that insect oil, especially *T. molitor* oil, could be utilized for therapeutic purposes in case of skin wounds. Furthermore, *H. illucens* larvae FAs showed antimicrobial properties against phytopathogens (Marusich et al. 2020; Mudalungu et al. 2023; Lückstädt and Panknin 2016).

All those researches may open the future for the industrialization of oil and fat from insects in different applications. Here in this review, we will treat in detail the most important aspects of fat from insects regarding biochemical composition and distribution. The overview of the physiological use of insects for fat and nutrient processing may help with the rearing and harvesting techniques when the target of the final products is fats or oils from insects.

## 2 Insects as Lipids Source

### 2.1 Processing of Insect Lipids

The extraction of insect's lipids requires specific organic solvents and methods. Their solubility and polarity in nonpolar solvents are key characteristics for the separation of complex lipid mixtures FAs contained in lipids bound with ester, which makes possible their hydrolyzation with acids, alkalis, or with hydrolytic enzymes. Neutral lipids like triacylglycerols, pigments, and waxes can rather be extracted with chloroform, ether, and benzene. Lipids from membranes are extracted by polar organic solvents such as methanol and ethanol. Those solvents contribute to reducing the hydrophobic interactions between the lipid molecules and, as a consequence to weakening hydrogen bonds and electrostatic interactions binding membrane proteins to lipids (Franco et al. 2021).

The procedure of lipids extraction steps consists of the homogenization of the sample with organic solvent, the separation of the organic phase that contains lipids from the aqueous phase, the nonlipid contaminants removal, and the removal of the organic solvent from the extract during the drying step (Franco et al. 2021).

There is no specific single standard method for the extraction of all lipid types (Pomeranz 2013; Gil et al. 2018; Franco et al. 2021). Indeed, the type of solvent and the extraction method should be chosen according to the type of target lipid in relation to the sample size and chemical structure. Small sample size increases the surface, permitting a better contact with the organic solvent, which raises its extractive yield. Organic solvents like diethyl ether or hexane cannot easily enter tissues, resulting in a noncomplete lipid extraction in some cases (Franco et al. 2021).

The water implication as a solvent in lipid extraction may lead to elevated costs (Franco et al. 2021). The access of the organic solvent into lipids is made easy with the implication of acid or basic hydrolysis, an important and mandatory step to separate lipids from proteins or carbohydrates and to break down emulsified fats. The key important property of the solvent is the high solubility for lipids and the low solubility for amino acids, proteins, and carbohydrates. It deactivates enzymes and prevents undesirable reactions (Franco et al. 2021). It must easily penetrate and evaporate with a low boiling point. The most usually implicated solvents or a mixture of solvents are acetone, ethanol, methanol, n‐butanol, chloroform, acetonitrile, dichloromethane, ethers, benzene, hydrocarbons, and hexane (Franco et al. 2021).

Diverse currently available methodologies reported in the literature for lipid extraction might also be applied to insect lipids (Franco et al. 2022; Tzompa‐Sosa et al. 2014; Laroche et al. 2019; Saviane et al. 2021; Ferdousi et al. 2023): Soxhlet method, the Folch method, aqueous extraction, supercritical fluid extraction method, the Matyash method, aqueous enzymatic method, acid fermentation method.

#### 2.1.1 Traditional Lipid Extraction Methods

**FIGURE 3:**
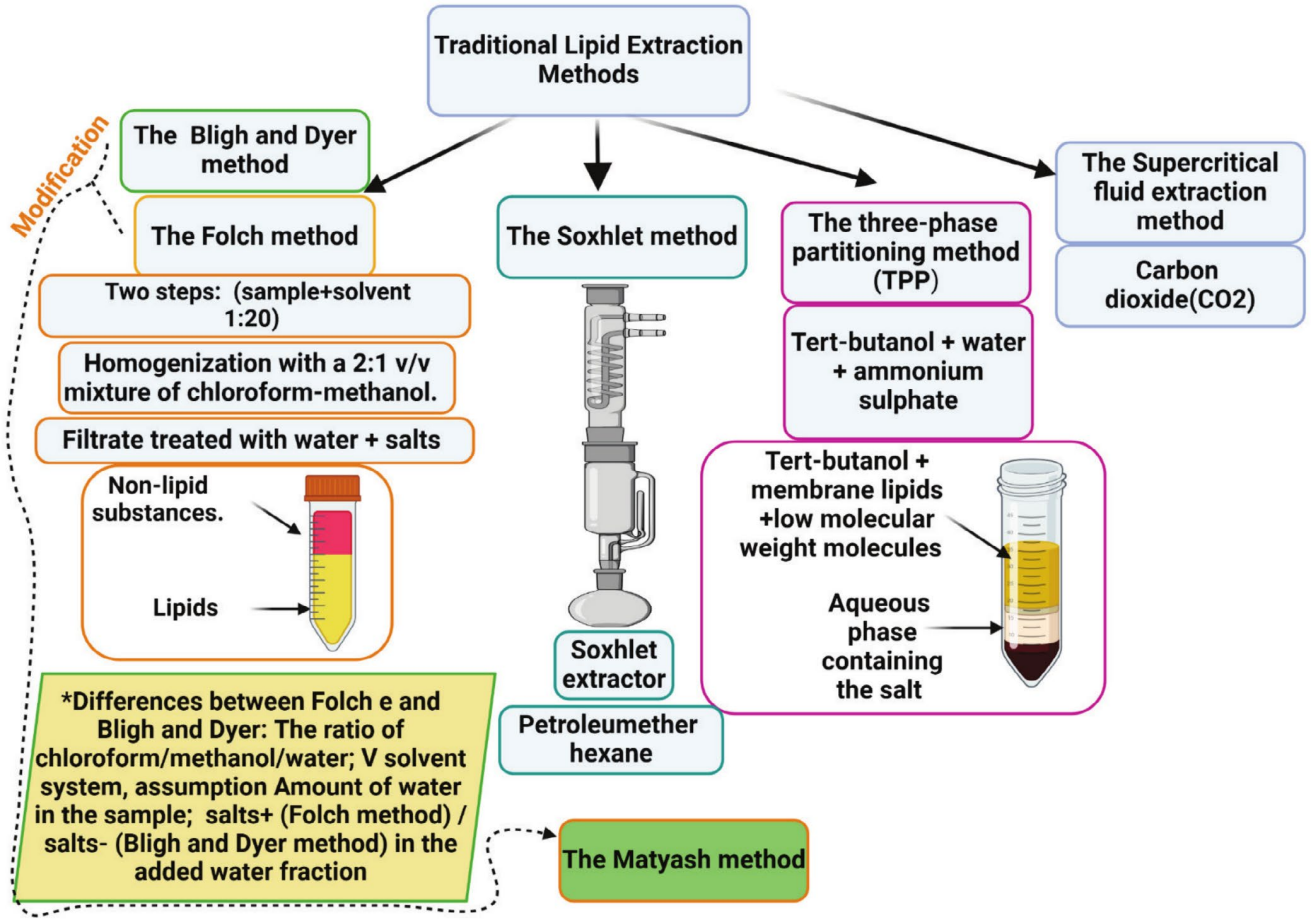
Main traditional lipid extraction methods for oil/fat from edible insects.

**FIGURE 4:**
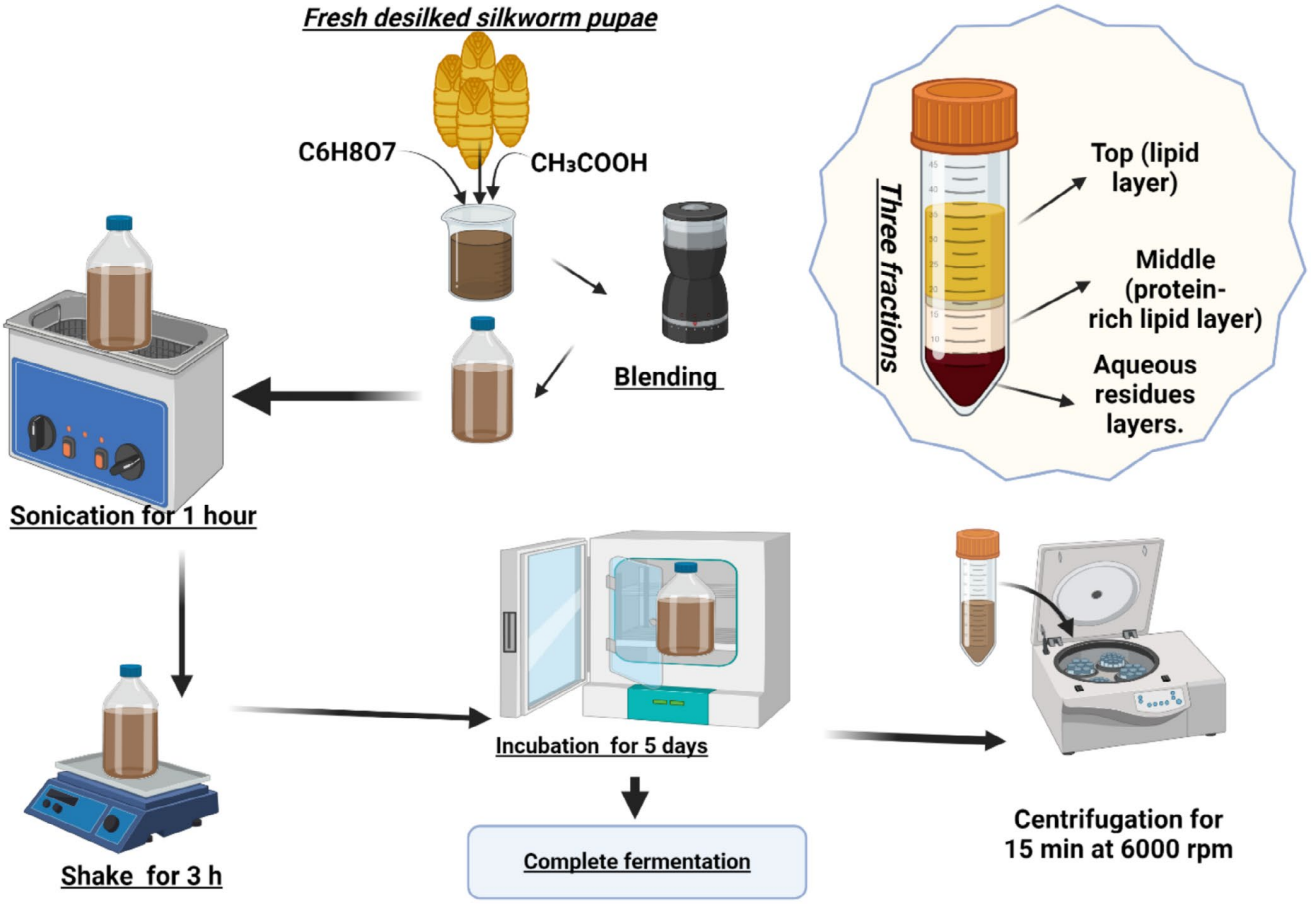
Extraction of oil from silkworm pupae (Figure created with Bioeditor according to Ferdousi et al. 2023).

Franco et al. (2021) have previously summarized and detailed the main traditional methods for oil/fat insect extraction. The main ones are represented by Figures 3 and 4.

The Folch method, modified and adopted by several extraction protocols, consists initially of two steps; the sample, after being homogenized in a 1:20 ratio with the solvent made of a 2:1 v/v mixture of chloroform‐methanol, is treated with water and a probable salts addition (Franco et al. 2021). The lower resulting phases contain lipids, while the upper phase consists of nonlipid substances (Franco et al. 2021). The Matyash method, consisting of a modification of the Folch/Bligh and Dyer methods and using Methyltert‐butyl ether (MTBE) allows for better recovery of almost all important lipid classes and to obtain a more accurate lipid profile (Tafi 2022). Chloroform, containing chlorine, generates the creation of a protein interphase between the lipid and the polar phase; therefore, it is to be avoided and substituted with a more appropriate alternative such as MTBE (Tafi 2022). The three‐phase partitioning method (TPP) is conducted using a solution of water and tert‐butanol with ammonium sulfate (Tafi 2022). The two‐phase mixture obtained consists of an aqueous phase containing low molecular weight molecules and salt in the lower phase and the tert‐butanol upper phase containing membrane lipids (Tafi 2022). Extracting lipids with consecutive gravimetric measurements could be achieved with the Soxhlet method, consisting of a “Soxhlet extractor” with a nonpolar liquid such as hexane and petroleum ether, permitting the determination of the raw lipid content (Franco et al. 2021; Tafi 2022). The supercritical fluid extraction method, instead, encompasses carbon dioxide (CO_2_) as a solvent (Tafi 2022).

#### 2.1.2 Methods of Insect Surface Lipid Extraction

Gołębiowski et al. (2011) reviewed different methods and strategies for the analysis of lipid and their composition. Epicuticular lipids are usually extracted with hexane to remove external hydrocarbons (Gołębiowski et al. 2011). Extractions with hexane and chloroform were used to obtain *Semidalis flinti* and nymphs and pupae of the giant whitefly, *Aleurodicus dugesii*, cuticular lipids (Nelson et al. 2000, 2003) while the extraction with petroleum ether was used to obtain surface lipids from *Galleria mellonella*, *Dendrolimus pini*, *Acanthoscelides obtectus* , and *Calliphora vicina* (Gołebiowski, Maliński, Boguś, et al. 2008; Gołębiowski, Maliński, Nawrot, et al. 2008). Alternative methods to study insect cuticular lipid composition have been used to replace solvent extraction (Choe et al. 2012; Ferreira‐Caliman et al. 2012; Alnajim et al. 2019). Solid‐phase microextraction (SPME) can be combined with GC, GC–MS, HPLC, or LC–MS. This contributes to cost reduction in terms of solvent purchase and preparation time, improving detection limits. Column liquid chromatography is also used to separate cuticular lipids, particularly hydrocarbons. Among the cited methods in the review by Gołębiowski et al. (2011), the most promising techniques were GC coupled with mass‐spectrometric techniques and liquid chromatography. According to the authors qualitative and quantitative analysis of surface lipid insect composition can be carried out by the extraction of analytes from biological material followed by the separation of lipid class using TLC, column chromatography, HPLC‐LLSD, derivatization, and a concluding with a determination by GC and GC–MS.

#### 2.1.3 Industrial Extraction Method

The extraction of fats at industrial levels from fresh or dried larvae tracks diverse procedures. The BSFL dried biomass can be pressed by a screw press which works at 100°C to obtain fat and partially defatted meals resulting in fat and a press cake (Tafi 2022). BSFL fat from the press liquid will be a subject of a further filtration processes (Franco et al. 2021). Two different methods for fractioning BSFL using a screw press can be cited: dry and wet processing (Franco et al. 2021). In the first process BSFL are dried before pressing; in the second one, instead, fresh BSFL are subject to direct pressing (Franco et al. 2021). The processing of fresh larvae takes more time; a further drying and grinding step is required in order to reduce the moisture content and particle size, and the difficulty of the pressed liquid separation requires advanced equipment (Franco et al. 2022; Tafi 2022). The microwave‐dried larvae are easier to press; however, wet heavy larvae contain both fat and water, which require more processing time in order to release also the water (Franco et al. 2021).

The silkworm pupae ( *B. mori* ) oil was extracted for the first time by acid fermentation. The simplified extraction method is shown in Figure 4 as described in Ferdousi et al. (2023): citric acid and acetic acid were added to desilked fresh pupae and blended with a grinder. After being transferred into a glass container, the mixture was sonicated for 1 h in an ultrasound cleaner, then shaken for 3 h in an orbital shaker, and finally kept in an incubator for 5 days at 40°C–45°C till complete fermentation (Ferdousi et al. 2023). A centrifugation step of the mixture is necessary (15 min at 6000 rpm) in order to obtain three fractions: the top layer (lipid), middle layer (protein‐rich lipid), and bottom layer (aqueous residues; Ferdousi et al. 2023). Oil/lipid layer and protein‐rich lipid layers contained in the top and middle layer were pipetted out then mixed with n‐hexane. The final mixture, after being shaken for 30 min, was filtered through Whatman filter paper (Ferdousi et al. 2023). The resulting residue solvent was volatilized by a rotary evaporator, and the resulting oil was heated in the oven at 80°C (Ferdousi et al. 2023). The silkworm oil obtained by acid fermentation could be a potential candidate as edible oil after extensive research on the method to increase the percentage of oil yield and to reduce time through modifications in the extraction steps (Ferdousi et al. 2023). The method was found by the authors, adopting suitable chemicals and reagents, to be suitable for large or industrial scale lipid extraction (Ferdousi et al. 2023). Conventional solvents such as methanol and ethanol in several lipid extraction methods were described in the literature and recommended as alternative environmentally friendly.

The common oil extraction method, supercritical CO_2_ (SC‐CO_2_), presents the method with low ecological impact and working cost (Franco et al. 2021). The TPP is considered to be efficient for extracting oil from oleaginous seeds and kernels (Franco et al. 2021). Organic solvents for lipid extraction were mostly performed on edible insects (Franco et al. 2021). For example, Tzompa‐Sosa (2014) used the Soxhlet, the aqueous, and the Folch extraction methods to extract lipid from four insect species ( *A. diaperinus* , *A. domesticus* , *Blaptica dubia*, and *T. molitor* ). Authors extracted the lowest lipid yield with the aqueous ion in all edible insects. Soxhlet and Folch methods instead produced similar yields (Franco et al. 2021; Tzompa‐Sosa et al. 2014). Zhao et al. (2016) obtained similar defatting efficacies from *T. molitor* with ethanol or a mixture of isopropanol and hexane (lipid extraction yield of about 30%). The application of the SC‐CO_2_ method to extract *T. molitor* lipids had a defatting range of 21%–95%, in relation to the applied duration, pressure, and temperature (Purschke et al. 2017; Laroche et al. 2019; Franco et al. 2021). The SC‐CO_2_ method is solvent‐free and reduces the lipid components oxidation.

As previously demonstrated in various studies, the lipid extract quality is significantly affected by the extraction technique (Franco et al. 2021). Therefore, the lipid extraction method has to be carefully selected (Tzompa‐Sosa et al. 2019). The aqueous oil extraction makes a green alternative to conventional oil extraction with n‐hexane and produces high quality proteins and oil with no further needed oil refinement (Latif et al. 2011; Mat Yusoff et al. 2015; Tzompa‐Sosa et al. 2016; Tzompa‐Sosa et al. 2019; Kumar et al. 2017; Preece et al. 2017). This method, according to Tzompa‐Sosa et al. (2014, 2019) had great impacts on the extracted oil yield and negligible effect on the extracted oil FA composition.

Recently, studies on the performance of the aqueous extraction method have been conducted to extract and characterize proteins from five diverse insect species (Yi et al. 2013; Tzompa‐Sosa et al. 2014). In this extraction, Tzompa‐Sosa et al. (2016) obtained three main fractions: a liquid fraction, an insoluble fraction, and an oil‐in‐water emulsion. The lipid fraction, here, was obtained as a by‐product along with some protein‐rich fractions. The lipid composition of extracts obtained by the aqueous method studied Tzompa‐Sosa et al. (2014), was compared with two other extraction methods (Soxhlet with industrial application and Folch analytical method generally used at laboratory scale) of four insect species. As it was previously reported in the literature, lipid content and types in insects vary in relation to their life stage and species (Tzompa‐Sosa et al. 2014). Total lipid content for caterpillars (Lepidoptera) varies from 8.6 to 15.2 g/100 g compared with grasshoppers and correlated species (Orthoptera) with low lipid content ranging from 3.8 to 5.3 g/100 g fresh insects (Tzompa‐Sosa et al. 2014). Crude lipids extracted from insects by organic solvent‐based extractions are composed of various types of lipids such as phospholipids, triacylglycerols, glycolipids, and sterols. About 80% of the several lipid types present in the extracted lipid are present in the form of triacylglycerols (Tzompa‐Sosa et al. 2014). Triacylglycerols serve as an energy deposit for periods of high energy request, like prolonged flights (Tzompa‐Sosa et al. 2014). The other lipid types are phospholipids, a class present in crude insect lipid, playing an important role in the structure of cell membranes along with cholesterol, which acts as a precursor for steroid hormones, vitamin D, and bile salts. Phospholipid content in crude fat varies between insect species and their life stages, and it is usually below 20% (Tzompa‐Sosa et al. 2014). Diverse studies on the cholesterol content in termites' lipid (*Macrotermes bellicosus*), caterpillars (*Imbrasia belina*), insects consumed in Nigeria; *Zophobas morio* and *T. molitor* from the Czech Republic (Kourimská and Adámková 2016; Sabolová et al. 2016) were conducted.

Industrially, lipids from vegetable and animal fonts are extracted with nonpolar solvent dissolving lipids or with aqueous extractions based on lipids insolubility in water (Tzompa‐Sosa et al. 2014). Comparing with nonpolar solvent extractions, aqueous‐based lipid extraction is industrially used for animal fat and vegetable oils extraction because of quality, environmental, and safety requirements, and it produced lower yields (Tzompa‐Sosa et al. 2014). The used solvents comprise petroleum naphtha, carbon disulfide, trichloroethylene, benzene, pentane, alcohol, SC‐CO_2_, and specifically commercial hexane (Tzompa‐Sosa et al. 2014). Saviane et al. (2021) tested diverse extraction methods for *H. illucens* and then applied the best one to *B. mori* pupae. The authors demonstrated that hot pressing of larvae/pupae maximized the oil yield and fat quality both in *H. illucens* and *B. mori* pupae. As the method of extraction has an effect on the types of extracted lipids, it consequently affects FA composition (Liya 2015).

The supercritical fluid extraction method was found advantageous for food products processing, reducing solutes oxidation, removing extracts without solvent, the possibility to extract thermosensitive components, and nontoxic and nonexplosive solvent conditions (Franco et al. 2021).

In the future of introducing insect fat in appropriate quantities into feedstuff represents great advantages in feed industry. The extraction method, insect species and their rearing substrate, have a great impact on extracted oil yield quantity and quality and its antimicrobial activities (Mohamed et al. 2022). Oil profile of insects that can be reared on vegetable substrates and with significant economic interest, can be mainly composed of unsaturated FAs with very interesting properties (Saviane et al. 2021). Such rearing substrate can be controlled and improved to affect FA composition of insects (Saviane et al. 2021).

Traditional methods use organic which can leave residues causing environmental hazards with further costly waste disposal. Solvent‐free, mechanical pressing frequently results in lower lipid yields. While SC‐CO_2_ extraction which is a cleaner alternative, demands expensive equipment. The TPP instead despite its efficiency for protein‐lipid separation it can be complex and require additional processing steps.

In the other hand, emerging green technologies have numerous advantages over conventional methods, particularly in terms of sustainability and efficiency. It aims to improve sustainability by minimizing chemical waste, reducing solvent use, and enhancing efficiency with methods such as enzymatic hydrolysis and ultrasound‐assisted extraction. The microwave‐assisted extraction accelerates lipid release, which makes the process more energy‐efficient.

However, conventional methods like solvent‐based extraction are the most used due to their recognized efficiency and lower costs.

### 2.2 Origin, Composition, Processing, and Application of Insect Lipids From the View of Both Research and Industry

Harvesting edible insects from the wild compared to those obtained through intentional breeding is challenging as there are no guarantees for their safety and quality (Pilco‐Romero et al. 2023). Moreover, direct harvesting can cause the extinction of some insect species (Pilco‐Romero et al. 2023). The difficulties of edible insect rearing under artificial conditions and the possibility of pathogen spread in captive populations must be considered (Baiano 2020).

The most common insects consumed around the world are beetles in adult and larval stages (Coleoptera), caterpillars (Lepidoptera), consumed almost entirely as caterpillars mostly in sub‐Saharan Africa, Hymenoptera, which are consumed mostly in their larval or pupal stages (bees, wasps, and ants), especially common in Latin America, followed by Orthoptera, including grasshoppers, locusts, and crickets; Hemiptera (leafhoppers, planthoppers, cicadas, scale insects, true bugs, etc.); and at minor percentage Isoptera, mainly termites, which are mostly consumed in the mature stage; while Diptera (flies), Odonata (dragonflies), and other order groups are consumed at lower percentages (Benzertiha et al. 2020; Rodríguez‐Miranda et al. 2019; Marcucci 2020).

A worldwide literature inventory, including Western countries and temperate regions, was conducted by Jongema (2017). The author, listed 1900 worldwide edible insect species. Many kinds of edible coleopteran were cited including larvae and adults of dung beetles, wood‐boring larvae and aquatic beetles. The palm weevil from the genus of *Rynchophorus*, an important palm pest that is distributed in Africa, South America and southern Asia, is the most popular edible beetles. The principal palm weevils are *R. phoenicis* (found in tropical and equatorial Africa) and *R. ferrugineus* (found in Asia; Indonesia, Japan, Malaysia, Papua New Guinea, the Philippines and Thailand) and *R. palmarum* (in the tropical Americas; Central America and West Indies, Mexico and South America) (Benzertiha et al. 2020). Larvae of Tenebrionidae family such as the yellow mealworm ( *T. molitor* ), the superworm (*Z. morio*), and the lesser mealworm ( *A. diaperinus* ) are reared mainly as feed but they are also offered as human food in dedicated markets (Benzertiha et al. 2020).

Moths are consumed in larval stages while butterflies are consumed as larval, moth, and adult stages. The bogong moth *Agrotis infusa* is eaten by Indigenous Australians. *I. belina* or the mopane caterpillar is the most popular economically important consumed caterpillar (Benzertiha et al. 2020).

Other species were also reported as edible insects in Angola, Botswana, Cameroon, Congo, South Africa, Zambia, and Zimbabwe, such as *Imbrasia ertli* and *Imbrasia epimethea* (Table 1). The House cricket *A. domesticus* (Gryllidae), globally distributed due to human activity, is actually authorized in feed except for ruminants in Europe, but it was also reported for food consumption in Thailand and Mexico. Table 1 reports some species of edible insects around the world.

**TABLE 1:** Origin, composition, processing, and application of different insects and different insect lipids.

Insect species	Common names	Family	World consumption distribution	Life stages	Application	Lipids characterization	References
*Acheta domesticus*	House cricket	Gryllidae	Thailand, Mexico, globally distributed primarily due to human activity	Larva/adult	Feed/food	Fat 12.2%	Tzompa‐Sosa, Dewettinck, Provijn, et al. (2021); Tzompa‐Sosa, Dewettinck, Gellynck, and Schouteten (2021); van Huis and Tomberlin (2017); Seni (2017)
*Gryllodes sigillatus*	Tropical house cricket			Larva/adult	Feed	Fat 13.3%	Tzompa‐Sosa, Dewettinck, Provijn, et al. (2021); Tzompa‐Sosa, Dewettinck, Gellynck, and Schouteten (2021)
*Gryllus assimilis*	Jamaican field cricket		Brazil, North America	Larva/adult	Feed	Fat 7.00%–32.0%	Tzompa‐Sosa, Dewettinck, Provijn, et al. (2021); Tzompa‐Sosa, Dewettinck, Gellynck, and Schouteten (2021); Ribeiro Soares Araújo et al. (2019); Jongema (2017)
*Gryllus bimaculatus*	Two‐spotted cricket		Guinea Bissau, Sierra Leone, Guinea, Liberia, Benin, Togo, Nigeria, DRC, Kenya, South Sudan, Zambia, Thailand, Asia, India	Larva/adult	Feed/Food	Fat 28.90%	Tzompa‐Sosa, Dewettinck, Provijn, et al. (2021); Tzompa‐Sosa, Dewettinck, Gellynck, and Schouteten (2021); Seni (2017); Jongema (2017)
*Teleogryllus Occipitalis*			Asia	—	Food	—	Seni (2017)
*Teleogryllus mitratus*			Indonesia, Thailand	—	Food	—	Seni (2017); Tang et al. (2019); Jongema (2017)
*Brachytrupes portentosus*			Thailand	—	Food	18.70 crude fat	Seni (2017)
*Imbrasia (Gonimbrasia) belina*	Mopane worms	Saturniidae	Nigeria, Malawi; South Africa; Southern Africa; Zambia; Zimbabwe	Larva	Food	15.15–23.38	Jongema (2017); Kolobe et al. (2023)
*Usta terpsichore*	Mopane caterpillar		Angola, D.R. Congo	Larva	Food	—	Jongema (2017)
*Imbrasia ertli*	Mopane caterpillar		Zambia, South Africa, Cameroon, Congo, CA Republic, Zimbabwe, Botswana, Angola	Larva	Food	—	Raheem et al. (2019); Jongema (2017)
*Imbrasia epimethea*	Mopane caterpillar		DRC, Zambia, South Africa, Cameroon, Congo, CA Republic, Zimbabwe	Larva	Food	13.3	Raheem et al. (2019); Meyer‐Rochow et al. (2021); Jongema (2017)
*Samia ricinii*	Eri silkworm		Northeast India	Pupa	Food	77.00*crude fat	Seni (2017); Tang et al. (2019); Jongema (2017)
*Attacus ricinii*	Eri silkmoth		India	—	Food	—	Seni (2017)
*Antheraea assamensis*	Muga silkworm		India	Larva/pupa	Food	—	Seni (2017); Jongema (2017)
*Antheraea pernyi*	Tussar moth		China; India	Pupa	Food	—	Feng et al. (2020); Seni (2017); Jongema (2017)
*Philosomia ricini*	Eri silkmoth.		India	—	Food	—	Seni (2017)
*Acrida lata*	Grasshopper	Acrididae	Korea	Adult	Food	—	Jongema (2017)
*Oxya yezoensis*	Grasshopper		Japan	—	Food	—	Jongema (2017)
*Oxya japonica*	Grasshopper		Thailand; Japan	Adult	Food	—	Jongema (2017)
*Oxya velox*	Rice grasshopper		Japan; Korea	Adult	Food	—	Jongema (2017)
*Oxya sinuosa*	Grasshopper		Korea	—	Food	—	Jongema (2017)
*Boopedon flaviventris*	Boopies		Mexico	Nymph/adult	Food	—	Jongema (2017)
*Schistocerca gregaria*	Desert locusts		Zambia, South Africa, North Africa, Southern Europe, India, Middle East, Cameroon, Congo, Botswana, Tanzania, Sudan, Uganda, Ethiopia, Kenya, Sierra Leone, Morocco, Guinea, Lesotho, Mauritania, Somalia, Eritrea, Guinea, Bissau	—	Food/feed	Fat 12%–32.3%	Raheem et al. (2019); Siddiqui et al. (2023)
*Locusta migratoria*	Migratory locust		Zambia, Cameroon, Congo, Zimbabwe, Sudan, South Sudan, Thailand; China	Adult	Food	Fat 20.34%	Kelemu et al. (2015); Raheem et al. (2019); Feng et al. (2020); Siddiqui et al. (2023); Jongema (2017)
*Heiroglyphus bannian*	Rice grasshopper		India	Adult	Food	—	Seni (2017)
*Atta Mexicana*	Leafcutting ant	Formicidae	Mexico	Adult	Food	39	Seni (2017); Jongema (2017)
*Oecophylla* spp.	Weaver ant		Cameroun, Congo	—	Food	—	Jongema (2017)
*Polymachis dives*	Black		China, Bangladesh, India, Malaysia and Sri Lanka	—	Nutritional ingredient	—	Feng et al. (2020); Seni (2017)
*Oecophylla smargdina*	Weaver ant		Malaysia and Sri Lanka; India, Thailand; China	All stages	Food		Seni (2017); Jongema (2017)
*Dorylus orientalis*	Asian army ant		North‐East India	Eggs, adults	Food	—	Doley and Kalita (2012); Seni (2017); Devi et al. (2022)
*Atta cephalotes*	Leafcutter ant		Brazil Colombia Guyana Honduras Nicaragua Mexico	Alate female soldier winged adults	Food	—	Seni (2017); Jongema (2017)
*Phyllophaga nebulosa*	July beetles	Scarabaeidae	Ghana	—	Food	—	Jongema (2017)
*Copris nevinsoni*	Dung Beetle		Thailand	—	Food	13.60 *crude fat	Tang et al. (2019); Fontaneto et al. (2011); Raksakantong et al. (2010); Jongema (2017)
*Holotrichia parallela*	Large black chafer		Thailand, China	Adult	Food	3.76*crude fat	Yang et al. (2014); Jongema (2017)
*Oryctes boas*	Rhinoceros Beetle		Nigeria, Ivory Coast, Sierra Leone, Guinea, Liberia, Guinea Bissau DRC, Congo, South Africa, Botswana, Namibia	Larva	Food	1.5	Raheem et al. (2019); Jongema (2017)
*Oryctes Owariensis*	Rhinoceros beetles		DRC, South Africa, Congo, Ivory Coast, Sierra Leone, Guinea, Ghana, Equatorial Guinea, Guinea Bissau	Larva	Food	53.8	Raheem et al. (2019); Jongema (2017)
*Oryctes rhinoceros*	Coconut beetle		Solomon Islands (DeFoliart 2002)	Larva	Food	10.8	Meyer‐Rochow et al. (2021); Jongema (2017)
*Macrotermes falciger*	Termite	Termitidae	Zambia, Zimbabwe, Burkina Faso, Burundi, Benin	Winged adult soldier queen	Food	—	Raheem et al. (2019); Jongema (2017)
*Macrotermes natalensis*	Termite		DRC, Cameroon, Congo, CA Republic, Nigeria, Burundi, South Africa, Zimbabwe, Nigeria, Malawi	Winged adult queen	Food	—	Raheem et al. (2019); Jongema (2017)
*Macrotermes bellicosus*	Termite		DRC, Cameroon, Congo, CA Republic, Nigeria, C Ivory Coast, Kenya, São São Tomé e Príncipe, Guinea, Togo, Liberia, Guinea Bissau, Burundi	All stages	Food	Fat 26.30–48.00	Meyer‐Rochow et al. (2021); Kolobe et al. (2023); Tang et al. (2019); Raheem et al. (2019); Jongema (2017)
*Macrotermes* spp.	Winged termites		DRC, Zambia, Zimbabwe, Nigeria, Tanzania, Malawi, Senegal, Uganda, Côte d'Ivoire, Guinea, Ghana, Togo, Burundi, Benin	Winged adults soldiers	Food	—	Raheem et al. (2019); Jongema (2017)
*Odontotermes Obesus*			India, Mexico	—	—	—	Seni (2017)
*Brachygastra azteca*	Wasp	Vespidae	Mexico	Immatures	Food	—	Jongema (2017)
*Vespula*	Yellow jacket wasps		Japan	Immatures honey	Food	—	Jongema (2017); Seni (2017)
*Dolichovespula* spp.	Yellow jackets		Japan	—	—	—	Seni (2017)
*Vespula Lewisi*	Wasp		—	—	—	—	—
*Parachartegus apicalis*	Wasp		Mexico	—	Food	—	Jongema (2017)
*Polybia occidentalis*	Camoati		Brazil, Venezuela, Mexico	Larva/pupa	Food	—	Jongema (2017)
*Agonoscelis versicolor*	Pentatomid	Pentatomidae	Sudan	Adult	Food	—	Seni (2017); Jongema (2017)
*Euschistus egglestoni*	Stink bug		—	—	—	—	—
*Aspongubus viduatus*	Melon bug		Sudan	Oil	Food	45%	Mariod et al. (2011)
*Agonoscelis pubescens*	Sorghum bug		Sudan	Oil	Food	(60%)	Mariod et al. (2011)
*Ioba*		Cicadidae	Malawi, Malaisse	Adult	Food	—	Seni (2017); Jongema (2017)
*Platypleura*			Malawi	Adult	Food	—	Seni (2017); Jongema (2017)
*Pycna*			Malawi	Adult	Food	—	Seni (2017); Jongema (2017)
*Omphisa fuscidentalis*	Bamboo caterpillar	Crambidae	Thailand; Lao People's Democratic Republic; China; Asia	Larva	Food	—	Feng et al. (2020); Seni (2017); Jongema (2017)
*Ostrinia furnacalis*	Asian corn borer		Cina		Food	46.08%	Seni (2017); Jongema (2017)
*Chilo fuscidentalis*	Bamboo borer		China	Larva	Food	—	Seni (2017); Jongema (2017)
*Rhynchophorus phoenicis*	African palm weevil	Curculionidae	DRC, Cameroon, Congo, CA Republic, Nigeria, Angola, Ivory Coast, Niger, São Tomé e Príncipe, Guinea, Togo, Liberia, Benin, Guinea Bissau	Larva	Food	21.10–69.78	Fernando et al. (2023); Raheem et al. (2019); Jongema (2017); Kolobe et al. (2023); Fontaneto et al. (2011)
*Rhynchophorus ferrugineus*	Red palm weevil		Indonesia, Japan, Malaysia, Papua New Guinea, the Philippines and Thailand	Larva, pupa, adult	Food	35.2%–60.1%	Meyer‐Rochow et al. (2021); Jongema (2017)
*Rhynchophorus palmarum*	South American palm weevil		Central America And West Indies, Mexico and South America	Larva, pupa	Food	—	Seni (2017); Jongema (2017)
*Periplaneta australasiae*	Australian cockroach	Blattidae	Nigeria	Adult	Food	27.3	Hlongwane et al. (2020); Meyer‐Rochow et al. (2021)
*Periplaneta americana*	Cockroaches		Nigeria, Brazil, Mexico	Larva	Food	28.2	Hlongwane et al. (2020); Meyer‐Rochow et al. (2021); Jongema (2017)
*Blatta lateralis*	Shelfordella Lateralis		—	—	Feed	21.90* crude fat	Tang et al. (2019)
*Tenebrio molitor*	Yellow mealworms	Tenebrionidae	China; Serbia	Larva	Food/Feed	24.70–43.08	Feng et al. (2020); Seni (2017); Kolobe et al. (2023)
*Alphitobius diaperinus*	Lesser mealworm		Africa and the Mediterranean regions	—	Food/Feed	8.5	van Huis and Tomberlin (2017); Seni (2017)
*Zophobas morio*	Superworm		Brazil, Mexico	Larva	Feed/Food	35	Ribeiro Soares Araújo et al. (2019); Jongema (2017)
*Lethocerus indicus*	Giant water bug	Belostomatidae	Thailand, Myanmar, Malaysia, Vietnam, India	Eggs, nymph, adult	Food	—	Seni (2017); Jongema (2017)
*Sphaerodema rusticum*	Giant water bugs		Thailand, Indonesia	Adult	Food	—	Seni (2017); Jongema (2017)
*Lethocerus grandis*	Giant water bug		—	—	Food	—	Seni (2017)
*Apis mellifera*	Honeybee	Apidae	Botswana, Nigeria, Tanzania, Senegal, Sierra Leone, South Sudan, Togo, Lesotho, Benin DRC, Zambia, CA Republic, Nigeria, Sierra Leone, Ghana, Benin; China	Larva, adult, pupa	Food	1.7–5.5	Raheem et al. (2019); Feng et al. (2020); Meyer‐Rochow et al. (2021)
*Apis indica*	Indian honey bee		India, Thailand, Philippines, Indonesia	Larva, pupa, honey	Food	—	Seni (2017); Jongema (2017)
*Hoplophorion monogramma*	Treehopper	Membracidae	Mexico	Nymph, adult	Food	—	Jongema (2017)
*Umbonia reclinata*	Thorn bug		Mexico	Nymph, adult	Food	—	Jongema (2017)
*Comadia redtenbacheri*	Moth	Cossidae	Mexico	Larva	Food	—	Seni (2017); Jongema (2017)
*Xyleutes leuchmochla*	Witjuti grub		Australian aborigines	Larva	Food	—	Jongema (2017)
*Sphenarium purpurascens*	Grasshopper	Pyrgomorphidae	Mexico, Niger, West Africa	Nymph, adult	Food	Fat 10.8%	Siddiqui et al. (2023); Meyer‐Rochow et al. (2021); Seni (2017); Jongema (2017)
*Sphenarium histrio*	Grasshopper		Mexico, Niger, West Africa	—	Food	Fat 12%	Siddiqui et al. (2023); Meyer‐Rochow et al. (2021)
*Krizousacorixa azteca*	Waterboatman	Corixidae	Mexico	Egg, nymph, adult	Food	—	Jongema (2017)
*Ruspolia nitidula*	Large Conehead	Tettigoniidae	Uganda, central and eastern Africa, Zimbabwe	—	Food	Fat 46.3%	Seni (2017)
*Neoconocephalus palustris*	Marsh conehead		India	—	Food	—	Seni (2017)
*Metroxylon sagu*	Sago palm	Arecaceae	—	—	—	—	Seni (2017)
*Bombyx mori*	Domestic silk moth	Bombycidae	Korea, Thailand, China; India	Larva, pupa	Food/Feed	Fat 23.45%	Feng et al. (2020); Seni (2017); Ghosh et al. (2020); Jongema (2017)
*Callipogon barbatus*	Longhorned Beetle	Cerambycidae	Mexico	Larva, pupa, adult	Food	—	Jongema (2017)
*Pachilis gigas*		Coreidae	—	—	—	—	—
*Agabetes acuductus*	Diving beetle	Dytiscidae	India		Food		Seni (2017)
*Ephydra hians*	Alkali Fly	Ephydridae	Nord America	Pupa	Food	—	Jongema (2017)
*Ephemerella jianghongensis*	Mayfly	Ephemerellidae	China	Nymph, adult	Food	—	Seni (2017); Jongema (2017)
*Pectinophora gossypiella*	Pink bollworm	Gelechiidae	China	Larva	Food	—	Seni (2017); Jongema (2017)
*Aegiale hesperiaris*	Tequila giant skipper	Hesperiinae	Mexico	Larva	Food	—	Seni (2017); Jongema (2017)
*Hydrochera rickseckeri*	Ricksecker's Water Scavenger Beetle	Hydrophiloidea	India	—	Food	—	Seni (2017)
*Dendrolimus houi*		Lasiocampidae	China	—	Food	—	Seni (2017)
*Musca domestica*	House fly	Muscidae	Globally distributed primarily due to human activity	Larva, pupa	Feed/Food	11.49–24.31	van Huis and Tomberlin (2017); Jongema (2017); Kolobe et al. (2023)
*Euxoa auxiliaries*	Army Cutworm	Noctuidae	—	—	—	—	—
*Anaphe venata*	African silkworm	Notodontidae	Zambia, South Africa, Cameroon, Congo, CA Republic, Nigeria, Zimbabwe, Botswana, Angola	Larva	Food	—	Raheem et al. (2019); Jongema (2017)
*Protohermes grandis*	Dobsonfly	Neuroptera	Japan	Adult	Food	—	Jongema (2017)
*Arytaina mopane*	African mopane psyllid	Psyllidae	South Africa	—	—	—	Seni (2017)
*Sarcophaga (Neobelliaria) bullata*	Gray flesh fly	Sarcophagidae	—	—	—	—	—
*Hermetia illucens*	Black soldier fly	Stratiomyidae	Widely distributes	Larva	Feed	18.03–34.5	Jongema (2017)
*Copestylum anna*	Hoverfly	Syrphidae	Mexico	Larva	Food	—	Jongema (2017)

**FIGURE 5:**
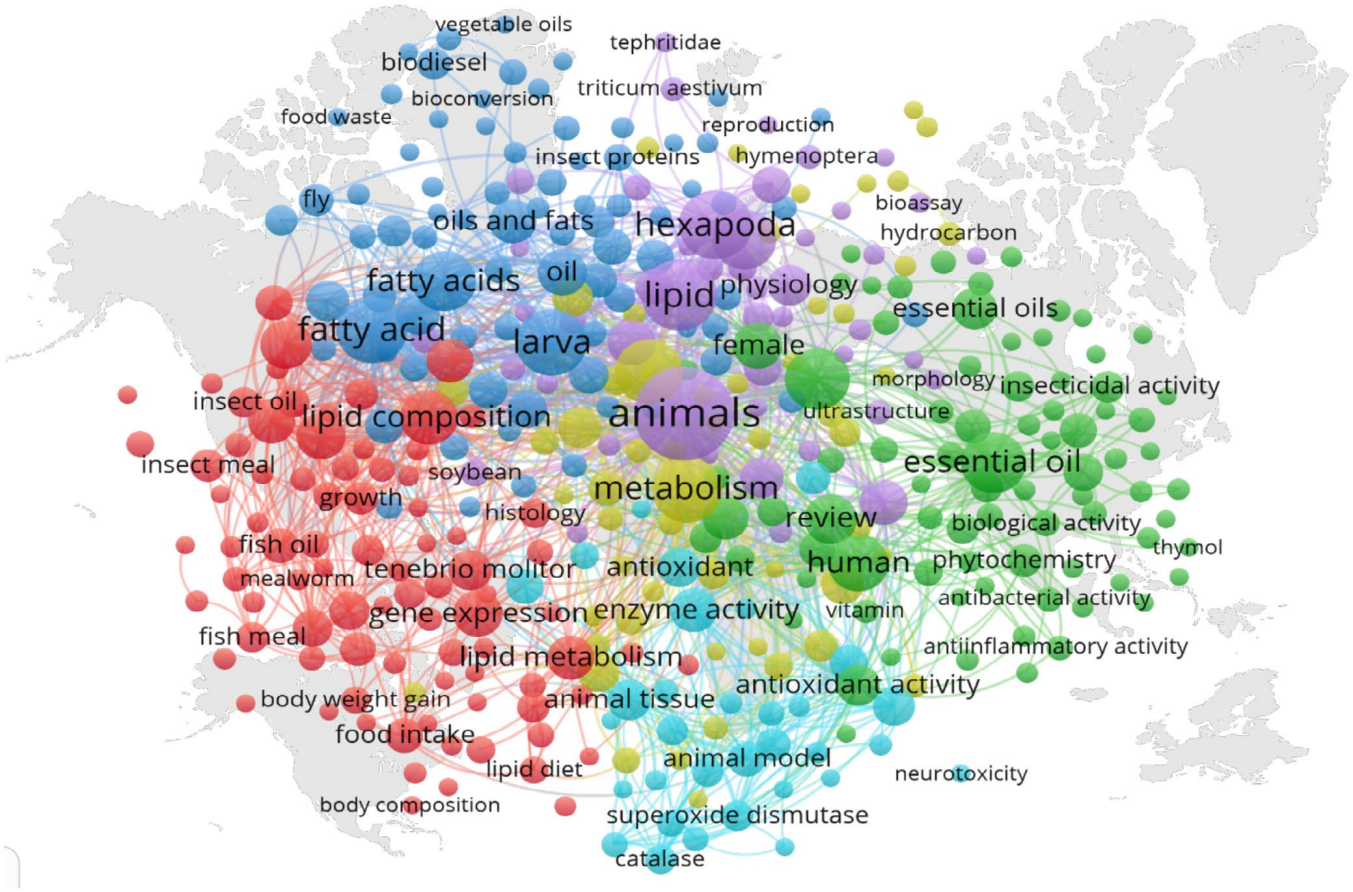
Bibliometric map of keywords in relation to lipid and fatty acid in studies from the world. Database used for bibliography extraction was Scopus. keywords: Insects AND lipid AND oil.

FB lipid content and composition are the result of numerous processes (Figure 5), including storage of dietary lipids, fat degradation and modification, and de novo synthesis.

**FIGURE 6:**
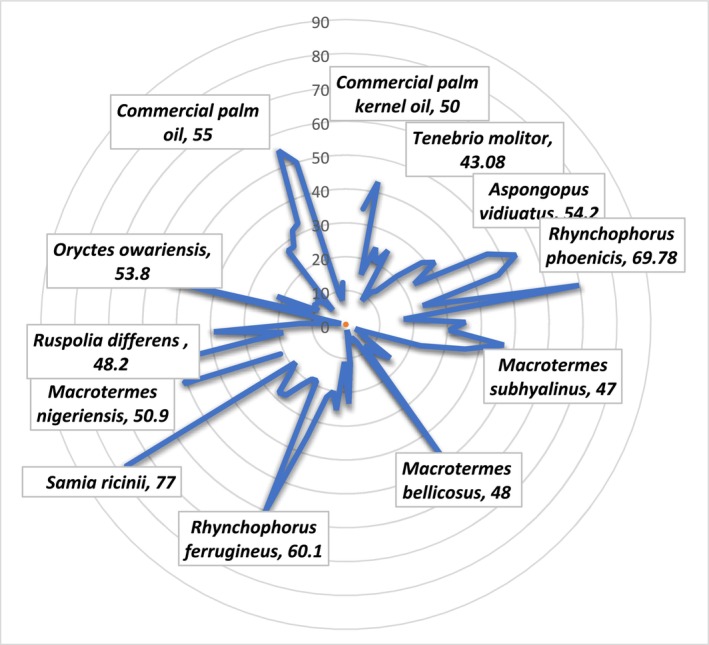
Lipid content in 100 g DW/WW of commonly used oils/fats in the market in comparison to the oils of different insects.

The amount of insect's lipids depends on species, diet, and metamorphic stage. The total insect's fat content has been reported to range from 2% to 62% (Lange and Nakamura 2023). The fat content of listed edible species in table and table are represented by Figure 6. The figure shows that the Coleoptera, Lepidoptera, and Isoptera orders have the most content of fat/oil at the same level of palm and palm kernel oil. The eri silkmoth larvae *Samia ricini* (Lepidoptera: Saturniidae) had the higher fat content in terms of crude fat (77% in crude fat of dry weight), followed by *Rhynchophorus phoenicis*, then *Rhynchophorus ferrugineus* and commercial palm oil. All three species were studied in their larval stage. Other three species showed higher fat content than commercial palm kernel oil; *A. vidiuatus* (adult) followed by *Oryctes owariensis* (Larvae) and *Macrotermes nigeriensis* (Adult).

## 3 Lipid Metabolism

The amount of lipids among insect species varies from 1% to 50% of the wet weight and is significantly affected by many factors such as the stage of development, sex, nutritional state, environmental temperature, hibernation, and migratory flight. A comparative characterization reporting the total lipid content and FA composition of a large number of insects from several orders at different development stages has been made (Table 2).

**TABLE 2:** Insect lipids yield (in % of DW = dry weigh or WW = wet weight), fatty acid compositions and related extraction methods, stage: larvae, pupae, or adult.

Insect species	Lipid content yield DW or WW%	Stage	C 12:0 (%) lauric acid	C 14:0 (%) myristic acid	C 16:0 (%) palmitic acid	C 16:1 (%) palmitoleic	Stearic acid (%) C 18:0	C18:1 (%) oleic acid	C18:2 (%) linoleic acid	Linolenic C18:3 (%)	Eicosa‐pentaenoic C20:5 (%)	C22:6 (%) Docosa‐hexaenoic	Omega 3 (n‐3)	Omega 6 (n‐6)	References
*Hermetia illucens*	18.03–34.5	L	8.37–49.3	2.9–6.9	9.44–16.1	0.99–3.5	NA	2.59–32.1	3.6–13.91	0.74–0.19	0.03–1.66	0.006–0.59	NA	8.16	Makkar et al. (2014); Kolobe et al. (2023)
*Tenebrio molitor*	24.70–43.08	L	0.15–0.58	1.58–5.89	10.96–28.20	0.76–3.01^b^ cis	0.68–2.96	28.08–42.01^b^ cis	19.5–51.51	0.33–2.98	0.02	0.07	NA	NA	Jajić et al. (2020); Kolobe et al. (2023), Son et al. (2019), Laroche et al. (2019), Otero et al. (2020), Wu et al. (2020), Benzertiha et al. (2019); Cito et al. (2017), Tang et al. (2019)
*Protaetia brevitarsis*	15.4	L	NA	0.07	1.81	1.61	0.27	7.85	0.91	0.01	NA	NA	NA	NA	Ghosh et al. (2017); Yeo et al. (2013)
*Musca domestica*	11.49–24.31	L	1.23	4.80	9.63–27.60	0.00	NA	5.09–21.75	8.84–16.44	0.81	2.94	NA	2.94	13.17	Kolobe et al. (2023); Anankware et al. (2021)
*Allomyrina dichotoma*	20.2	L	0.02	0.12	6.42	1.32	NA	7.94	0.69	0.01	0.01	NA	NA	NA	Ghosh et al. (2017)
*Teleogryllus emma*	25.1	A	0.02	0.18	3.06	0.91	NA	6.98	9.61	0.22	NA	NA	NA	NA	Ghosh et al. (2017)
*Alphitobius diaperinus*	8.5	L	0.34	1.57	26.68	1.06	NA	31.41	20.27	0.38	NA	0.002	NA	NA	Mattioli et al. (2024)
*Acheta domesticus house cricket*	12.2	A	0.028–0.7	0.107	5.870–23.4	0.153–1.3	NA	3.900	1.170	0.007	0.057	NA	NA	NA	Udomsil et al. (2019)
*Gryllodes sigillatus*	13.3	A	NA	0.80	24.61	NA	0.39	25.15	33.74	1.60	NA	NA	NA	NA	Tzompa‐Sosa, Dewettinck, Provijn, et al. (2021); Tzompa‐Sosa, Dewettinck, Gellynck, and Schouteten (2021)
*Gryllus bimaculatus*	11.90–20.74	A	0.04–0.045	0.05–0.271	2.16–27.73	0.17–0.295	NA	2.91–31.76	1.390—27.33	0.062–0.08	0–0.070	NA	0.08–1.15	4.25–27.92	Udomsil et al. (2019); Kolobe et al. (2023); Ghosh et al. (2017)
*Gryllus bimaculatus*	28.90	L	NA	NA	25.44	NA	8.74	25.86	37.05	NA	NA	NA	NA	NA	Zhao et al. (2021)
*Gryllus assimilis*	7.00–32.0	A	NA	0.82	24.50–25.85	NA	0.50	25.03–25.29	26.13–35.34	1.38	NA	NA	1.99	26.81	Kolobe et al. (2023); Tzompa‐Sosa, Dewettinck, Provijn, et al. (2021); Tzompa‐Sosa, Dewettinck, Gellynck, and Schouteten (2021)
*Bombyx mori*	23.45	P	NA	0.10	24.20	NA	NA	26.00	7.30	NA	NA	NA	NA	NA	Ghosh et al. (2020)
*Aspongopus vidiuatus*	54.2	A	NA	0.3	31.3	NA	3.5	45.5	4.9	NA	NA	NA	NA	NA	Mariod et al. (2011); Meyer‐Rochow et al. (2021)
*Pseudacanthotermes militaris*	46.6	A	NA	NA	26.0	NA	5.9	50.3	11.5	NA	NA	NA	NA	NA	Meyer‐Rochow et al. (2021); Kinyuru et al. (2013)
*Pseudacanthotermes spiniger*	47.3	A	NA	0.8	28.0	NA	6.1	49.3	10.5	NA	NA	NA	NA	NA	Meyer‐Rochow et al. (2021); Kinyuru et al. (2013)
*Imbrasia (Gonimbrasia) belina*	15.15–23.38	L	NA	NA	3.20–31.90	NA	NA	34.20	6.0	0.115 (γ), 0.380 (α)	NA	NA	3.70	1.60	Kolobe et al. (2023); Fontaneto et al. (2011); Bukkens (2020, 2005)
*Rhynchophorus phoenicis*	21.10–69.78	L	NA	NA	27.13–34.20	NA	NA	35.05	2.20–19.50	0.050 (γ), 0.015 (α)	NA	NA	0.00	2.20	Kolobe et al. (2023); Fontaneto et al. (2011); Bukkens (2005)
*Rhynchophorus bilineatus*	17.17 g oil/100 g FW	L	0.005	0.185	7.2	0.581	0.63	7.66	0.517	0.268	NA	NA	NA	NA	Köhler et al. (2020)
*Schistocerca gregaria*	12.97–35.30	A	NA	NA	23.26–26.90	NA	NA	31.70–40.87	10.10–14.04	NA	NA	NA	11.35–13.40	7.28–14.04	Kolobe et al. (2023)
*Locusta migratoria*	30.52	A	NA	NA	39.00–43.01	NA	NA	22.85–26.20	3.38–9.32	NA	NA	NA	6.74	10.71	Kolobe et al. (2023); Sanchez‐Muros et al. (2014); Fombong et al. (2021)
*Macrotermes subhyalinus*	44.82–47.00	A	15.4	NA	27.65–33.00	NA	NA	9.50–48.60	10.7 5–43.1	NA	NA	NA	NA	NA	Kolobe et al. (2023); Paoletti et al. (2003)
*Aspongopus nepalensis*	35.9	A	NA	0.4	32.3	NA	4.8	46.4	6.1	NA	NA	NA	NA	NA	Victor Benno Meyer‐Rochow
*Acheta* spp.	12.20–22.96	A	NA	NA	5.50–22.65	NA	NA	20.18–31.10	32.20–41.30	NA	NA	NA	0.01–0.39	2.08–42.63	Kolobe et al. (2023)
*Apis Dorsata*	6.2	P	NA	3.2	33.3	NA	11.2	47.7	0.8	NA	NA	NA	NA	NA	Meyer‐Rochow et al. (2021)
*Apis Dorsata*	3.1	A	NA	1.0	14.4	NA	14.4	61.0	2.2	NA	NA	NA	NA	NA	Meyer‐Rochow et al. (2021)
*Apis Florea*	7.2	P	NA	1.8	35.3	NA	8.8	47.6	1.0	NA	NA	NA	NA	NA	Meyer‐Rochow et al. (2021)
*Apis Florea*	5.4	A	NA	1.5	30.7	NA	9.7	49.7	1.1	NA	NA	NA	NA	NA	Meyer‐Rochow et al. (2021)
*Imbrasia truncata*	16.4	L	NA	0.2	24.6	NA	NA	7.6	7.6	0.076 (γ), 0.368 (α)	NA	NA	NA	NA	Meyer‐Rochow et al. (2021); Fontaneto et al. (2011); Bukkens (2005)
*Imbrasia epimethea*	13.3	L	NA	0.6	23.2	NA	NA	8.4	7.0	0.070 (γ), 0.351 (α)	NA	NA	NA	NA	Meyer‐Rochow et al. (2021); Fontaneto et al. (2011); Bukkens (2005)
*Bombus ignitus*	9.5	A	NA	2.6	16.1	NA	1.7	49.1	2.5	NA	NA	NA	NA	NA	Meyer‐Rochow et al. (2021)
*Bombus terrestris*	8.4	A	NA	3.8	15.2	NA	1.7	51.1	2.2	NA	NA	NA	NA	NA	Meyer‐Rochow et al. (2021)
*Macrotermes bellicosus*	26.30–48.00	D.A.	NA	0.2–2.17	38.35–46.54	2.10	2.86	12.84–41.74	5.03–34.42	3.90	NA	NA	NA	8.16	Meyer‐Rochow et al. (2021); Kolobe et al. (2023); Tang et al. (2019)
*Apis cerana*	6.1	L	NA	3.9	38.2	NA	8.1	46.9	0.5	NA	NA	NA	NA	NA	Meyer‐Rochow et al. (2021)
*Apis cerana*	6.3	P	NA	3.0	31.4	NA	10.6	49.8	0.9	NA	NA	NA	NA	NA	Meyer‐Rochow et al. (2021)
*Apis cerana*	4.2	A	NA	1.9	18.2	NA	12.1	57.7	2.6	NA	NA	NA	NA	NA	Meyer‐Rochow et al. (2021)
*Apis mellifera*	4.9	L	NA	2.4	37.3	NA	NA	47.5	NA	NA	NA	NA	NA	NA	Meyer‐Rochow et al. (2021)
*Apis mellifera*	5.5	P	NA	2.9	35.1	NA	NA	47.6	NA	NA	NA	NA	NA	NA	Meyer‐Rochow et al. (2021)
*Apis mellifera*	1.7	A	NA	0.6	14.4	NA	NA	45.2	7.8	NA	NA	NA	NA	NA	Meyer‐Rochow et al. (2021)
*Vespa velutina*	11.6	B	NA	6.0	31.9	NA	7.8	33.3	5.2	NA	NA	NA	NA	NA	Meyer‐Rochow et al. (2021)
Imbrasia belina	11.1–25.4	L	NA	0.5–1.2	22–46	NA	0.4–7.2	2–34.6	6.0–20	NA	NA	NA	NA	NA	Meyer‐Rochow et al. (2021)
*Vespa mandarinia*	20.2	B	NA	2.5	21.3	NA	5.0	27.7	33.7	NA	NA	NA	NA	NA	Meyer‐Rochow et al. (2021)
*Vespa basalis*	22.2	B	NA	1.4	15.8	NA	5.4	23.9	42.8	NA	NA	NA	NA	NA	Meyer‐Rochow et al. (2021)
*Zophobas morio*	35.0	L	0.46	1.87	30.32	0.67	NA	33.49	20.78	1.38	0.09	0.002	1.80	26.80	Mattioli et al. (2024); Jayanegara et al. (2020)
*Rhynchophorus ferrugineus*	35.2–60.1	L	NA	NA	NA	NA	NA	NA	NA	NA	NA	NA	NA	NA	Meyer‐Rochow et al. (2021)
*Meimuna opalifera*	19.00^a^	A	NA	1.99	2.47	0.28	52.53	0.92	NA	NA	NA	NA	NA	NA	Tang et al. (2019)
*Polyrhachis vicina*	22.00^a^	P	NA	0.60	17.67	8.77	4.30	61.43	2.07	0.80	NA	NA	NA	NA	Tang et al. (2019)
*Periplaneta australasiae*	27.3	A	NA	NA	NA	NA	NA	NA	NA	NA	NA	NA	NA	NA	Meyer‐Rochow et al. (2021)
*Periplaneta americana*	28.2	L	NA	NA	NA	NA	NA	NA	NA	NA	NA	NA	NA	NA	Meyer‐Rochow et al. (2021)
*Blatta lateralis*	21.90^a^	N	NA	1.90	26.80	3.30	18.10	38.00	8.70	0.70	NA	NA	NA	NA	Tang et al. (2019)
*Brachytrupens protentosus*	18.70	A	NA	1.61	35.79	0.71	0.13	3.40	58.37	NA	NA	NA	NA	NA	Tang et al. (2019)
*Samia ricinii*	77.00^a^ crude fat	L	NA	NA	26.97	1.78	5.53	17.33	5.11	43.06	NA	NA	NA	NA	Tang et al. (2019)
*Acheta confirmata*	21.14^a^	A	NA	26.10	5.50	2.40	1.20	31.10	32.20–0.322	1.70; 0.017 (α)	NA	NA	NA	NA	Tang et al. (2019); Yang et al. (2006)
*Xyleutes* sp.	NA	NA	NA	NA	NA	NA	NA	NA	NA	0 004 (γ)	NA	NA	NA	NA	Fontaneto et al. (2011); Bukkens (2005)
*Nasutitermes* sp.	NA	NA	NA	NA	NA	NA	NA	NA	NA	0.025 (α)	NA	NA	NA	NA	Fontaneto et al. (2011); Oyarzun et al. (1996)
*Oecophylla smargdina weaver*	NA	NA	NA	NA	NA	NA	NA	NA	NA	0.003 (α)	NA	NA	NA	NA	Fontaneto et al. (2011); Raksakantong et al. (2010)
*Antheraea assamensis*	NA	NA	NA	NA	NA	NA	NA	NA	NA	0.295 (α)	NA	NA	NA	NA	Fontaneto et al. (2011)
*Syntermes aculeosus*	NA	NA	NA	NA	NA	NA	NA	NA	0.248	0.069 (γ), 0.007 (α)	0.007	NA	NA	NA	Fontaneto et al. (2011), Paoletti et al. (2003)
*Morpho peleides*	NA	A	NA	NA	NA	NA	NA	NA	0.108	0.357 (α)	NA	NA	NA	NA	Fontaneto et al. (2011); Wang et al. (2006)
*Morpho peleides*	NA	L	NA	NA	NA	NA	NA	NA	0.167	0.417 (α)	NA	NA	NA	NA	Fontaneto et al. (2011); Wang et al. (2006)
*Macrotermes nigeriensis*	34.23–50.90	A	NA	0.6	31.4	NA	NA	52.5	7.6	NA	NA	NA	NA	NA	Victor Benno
*Nudaurelia oyemensis*	NA	NA	NA	NA	NA	NA	NA	NA	NA	0.057 (γ), 0.356 (α)	NA	NA	NA	NA	Fontaneto et al. (2011); Bukkens (2005)
*Chondracris rosea brunneri*	NA	NA	NA	NA	NA	NA	NA	NA	0.123	0.401 (α)	NA	NA	NA	NA	Fontaneto et al. (2011); Yang et al. (2006)
*Decticus verrucivorus*	NA	NA	NA	NA	NA	NA	NA	NA	0.446	0.000 (γ), 0.360 (α)	0.001	NA	NA	NA	Fontaneto et al. (2011)
*Gryllotalpa africana*	NA	NA	NA	NA	NA	NA	NA	NA	0.138	0.005 (α)	NA	NA	NA	NA	Fontaneto et al. (2011); Yang et al. (2006)
*Homorocoryphus nitidulus*	NA	NA	NA	NA	NA	NA	NA	NA	0.456	0.005 (γ), 0.161 (α)	NA	NA	NA	NA	Fontaneto et al. (2011); Womeni et al. (2009)
*Cybister limbatus*	NA	NA	NA	NA	NA	NA	NA	NA	0.133	0.015 (γ), 0.063 (α)	0.016	NA	NA	NA	Fontaneto et al. (2011); Yang et al. (2006)
*Hydrous cavistanum*	NA	NA	NA	NA	NA	NA	NA	NA	0.215	0.031 (α)	0.280	NA	NA	NA	Fontaneto et al. (2011); Yang et al. (2006)
*Lethocerus indicus*	NA	NA	NA	NA	NA	NA	NA	NA	0.090	0.034 (α)	0.019	NA	NA	NA	Fontaneto et al. (2011); Yang et al. (2006)
*Ruspolia nitidula*	46.3	A	NA	NA	NA	NA	NA	NA	NA	NA	NA	NA	NA	NA	
*Ruspolia differens*	48.20	A	NA	0.90	31.50	NA	5.50	24.60	31.20	NA	NA	NA	NA	NA	Zhao et al. 2021
*Protohermes grandis*	NA	NA	NA	NA	NA	NA	NA	NA	NA	0.004 (γ), 0.046 (α)	0.136	NA	NA	NA	Fontaneto et al. (2011)
*Gomphus vulgatissimus*	NA	NA	NA	NA	NA	NA	NA	NA	NA	0.003 (γ), 0.059 (α)	0.094	NA	NA	NA	Fontaneto et al. (2011)
*Brachytrupes portentosus*	18.70 crude fat	A	NA	1.61	35.79	0.71	0.13	3.40	NA	NA	NA	NA	NA	NA	Tang et al. 2019
*Stenopsyche griseipennis*	NA	NA	NA	NA	NA	NA	NA	NA	NA	0.002 (γ), 0.038 (α)	0.190	NA	NA	NA	Fontaneto et al. (2011)
*Termes* sp.	NA	NA	NA	NA	NA	NA	NA	NA	0.119	NA (γ), 0.025 (α)	NA	NA	NA	NA	Fontaneto et al. (2011); Raksakantong et al. 2010
*Philosamia ricini*	NA	NA	NA	NA	NA	NA	NA	NA	NA	NA (γ), 0.373 (α)	NA	NA	NA	NA	Fontaneto et al. (2011)
*Atta mexicana*	39.0	A	NA	NA	NA	NA	NA	NA	NA	NA	NA	NA	NA	NA	
*Copris nevinsoni*	13.60 crude fat	L	NA	1.76	28.97	2.75	13.29	47.66	3.92	0.017–0.84 (α)	0.129	NA	NA	NA	Tang (2019); Fontaneto et al. (2011); Raksakantong et al. (2010)
*Holotrichia parallela*	3.76 crud fat	A	NA	0.53	13.29	1.1	7.05	40.75	35.72	NA	NA	NA	NA	NA	Yang et al. (2014)
*Oryctes boas*	1.5	L	NA	NA	NA	NA	NA	NA	NA	NA	NA	NA	NA	NA	
*Oryctes owariensis*	53.8	L	NA	2.5	0.2	NA	NA	5.2	45.5	0.012 (γ), 0.041 (α)	NA	NA	NA	NA	Meyer‐Rochow et al. (2021); Fontaneto et al. (2011); Raksakantong et al. (2010)
*Oryctes rhinoceros*	10.8	L	NA	3.5	28.7	NA	NA	41.5	0.454–14.1	0.012	NA	NA	NA	NA	Meyer‐Rochow et al. (2021)
*Epophthalmia elegans*	9.14	L	NA	1.67	21.88	NA	7.85	16.97	4.46	NA	NA	NA	NA	NA	Zhao et al. (2021)
*Anax parthenope julius*	11.06	L	NA	1.18	24.41	NA	7.00	17.65	7.01	NA	NA	NA	NA	NA	Zhao et al. (2021)
*Ictinogomphus rapax*	10.59	L	NA	2.07	19.30	NA	6.98	19.01	7.49	NA	NA	NA	NA	NA	Zhao et al. (2021)
*Pantala flavescens*	10.4	L	NA	0.75	21.6	NA	8.55	11.98	9.59	NA	NA	NA	NA	NA	Zhao et al. (2021)
*Inictinogomphus clavatus*	11.9	L	NA	0.89	24.61	NA	7.74	20.58	6.66	NA	NA	NA	NA	NA	Zhao et al. (2021)
*Orthetrum pruinosum neglectum*	5.72	L	NA	2.34	17.57	NA	7.65	5.85	5.77	NA	NA	NA	NA	NA	Zhao et al. (2021)
*Stictochironomus pictulus*	NA	L	NA	4.70	16.10	NA	6.20	11.00	6.70	NA	NA	NA	NA	NA	Zhao et al. (2021)
*Anopheles albimanus*	NA	L	NA	1.94	28.03	NA	7.76	22.26	7.59	NA	NA	NA	NA	NA	Zhao et al. (2021)
*A. vestitipennis*	NA	L	NA	1.86	21.01	NA	6.62	22.06	12.28	NA	NA	NA	NA	NA	Zhao et al. (2021)
*A. darlingi*	NA	L	NA	1.26	25.42	NA	6.64	22.44	18.66	NA	NA	NA	NA	NA	Zhao et al. (2021)
*Cybister japonicus*	27.66	A	NA	3.41	12.01	NA	5.18	35.61	9.82	NA	NA	NA	NA	NA	Zhao et al. (2021)
*Dytiscus danmcus*	27.56	A	NA	2.86	21.63	NA	2.45	29.94	6.53	NA	NA	NA	NA	NA	Zhao et al. (2021)
*Hydrophilus aoninatus*	31.86	A	NA	9.16	19.09	NA	NA	1.83	1.98	NA	NA	NA	NA	NA	Zhao et al. (2021)
Coconut oil	33.00	NA	44.10–51.00	10.50–13.10	7.50–10.50	5.00–8.20	NA	NA	NA	NA	NA	NA	NA	NA	Aluyor et al. (2009); Franco et al. (2022)
Commercial palm oi	45.00–55.00	NA	0.20	1.10	44.00	39.00	NA	NA	NA	NA	NA	NA	NA	NA	Franco et al. (2022); Manciniet al. (2015); Marcus et al. (2013)
Commercial palm kernel oil	50.00	NA	47.80	16.30	8.50	15.40	NA	NA	NA	NA	NA	NA	NA	NA	Franco et al. (2022); Dijkstra (2016)
Beef	9.3	NA	NA	0.77	16.74	NA	9.53	10.52	36.10	6.16	NA	NA	NA	NA	Tang et al. (2019)
Chicken	7.2	NA	NA	1.33	22.67	0.27	8.00	41.33	14.00	0.67	NA	NA	NA	NA	Tang et al. (2019)
Pork	12.4	NA	NA	3.43	21.68	2.93	12.71	39.39	7.29	1.71	NA	NA	NA	NA	Tang et al. (2019)

Abbreviations: B, brood; D.A, dewinged adults; NA, not available.

^a^ Crude fat.

^b^ cis.

**FIGURE 7:**
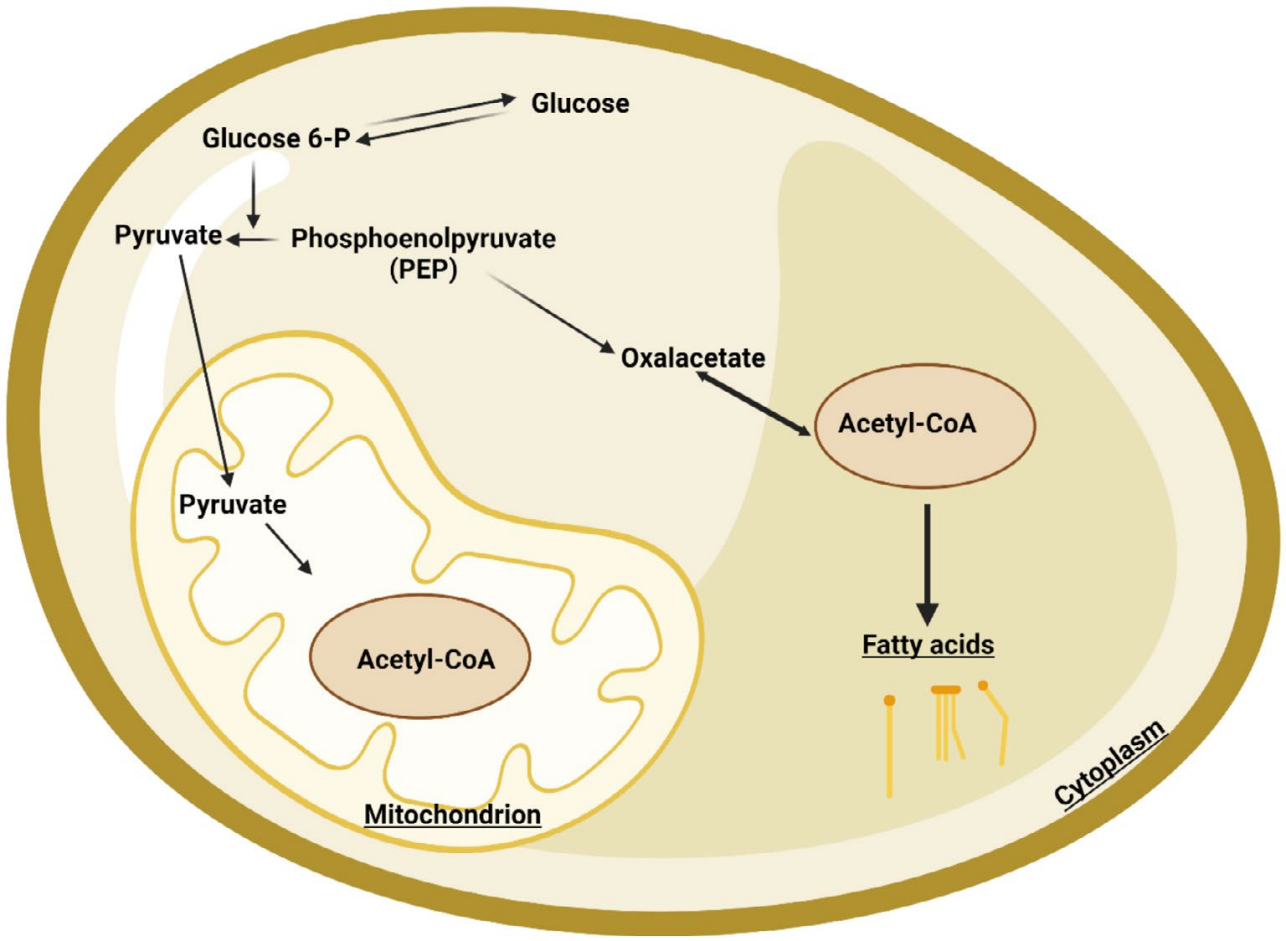
Metabolic pathway implicated in the conversion of carbohydrate into fatty acids in insects' fat body.

The FB, consisting of aggregates of cells forming lobes or sheets of tissue, is the principal storage site for insect lipids. The FB is distributed throughout the body, serving not only for the storage of carbohydrate, lipid, and protein, but it also serves as the major center for metabolism under the control of hormones, transcription factors, and secondary messengers. The FB arrangement in the abdomen, with large FB deposits in relation to the gut, facilitates the uptake of dietary nutrients. Oenocytes and adipocytes are the primary cells in relation to lipid metabolism, which starts with hydrolyzing dietary lipids. Lipid is then transported from the midgut to the FB where lipogenesis or lipolysis occurs. From the FB, lipid is transported to other sites requiring energy (Toprak et al. 2020). A scheme of insect lipid metabolism is given in Figure 7. After food consumption, lipid metabolism begins in the midgut where lipid compounds are first digested by lipases. The digested products are transported by Lpps to the targets organs such as the FB, muscles, and ovaries. FAs are transported to target cells by FA transport and binding proteins (Wrońska et al. 2023).

**FIGURE 8:**
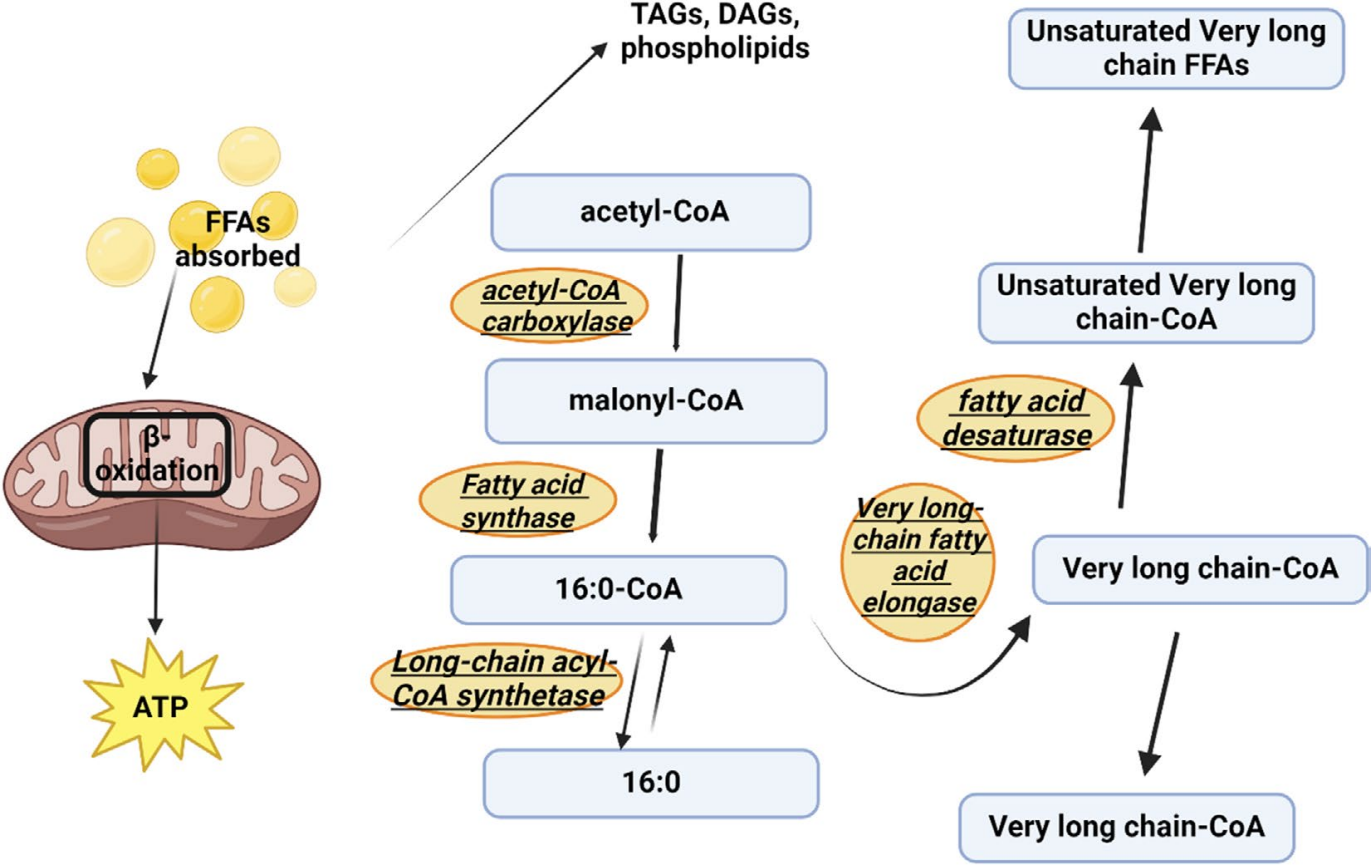
Fatty acid metabolism: FFA, free fatty acid; DAG, diacylglycerol; TAG, triacylglycerol.

**FIGURE 9:**
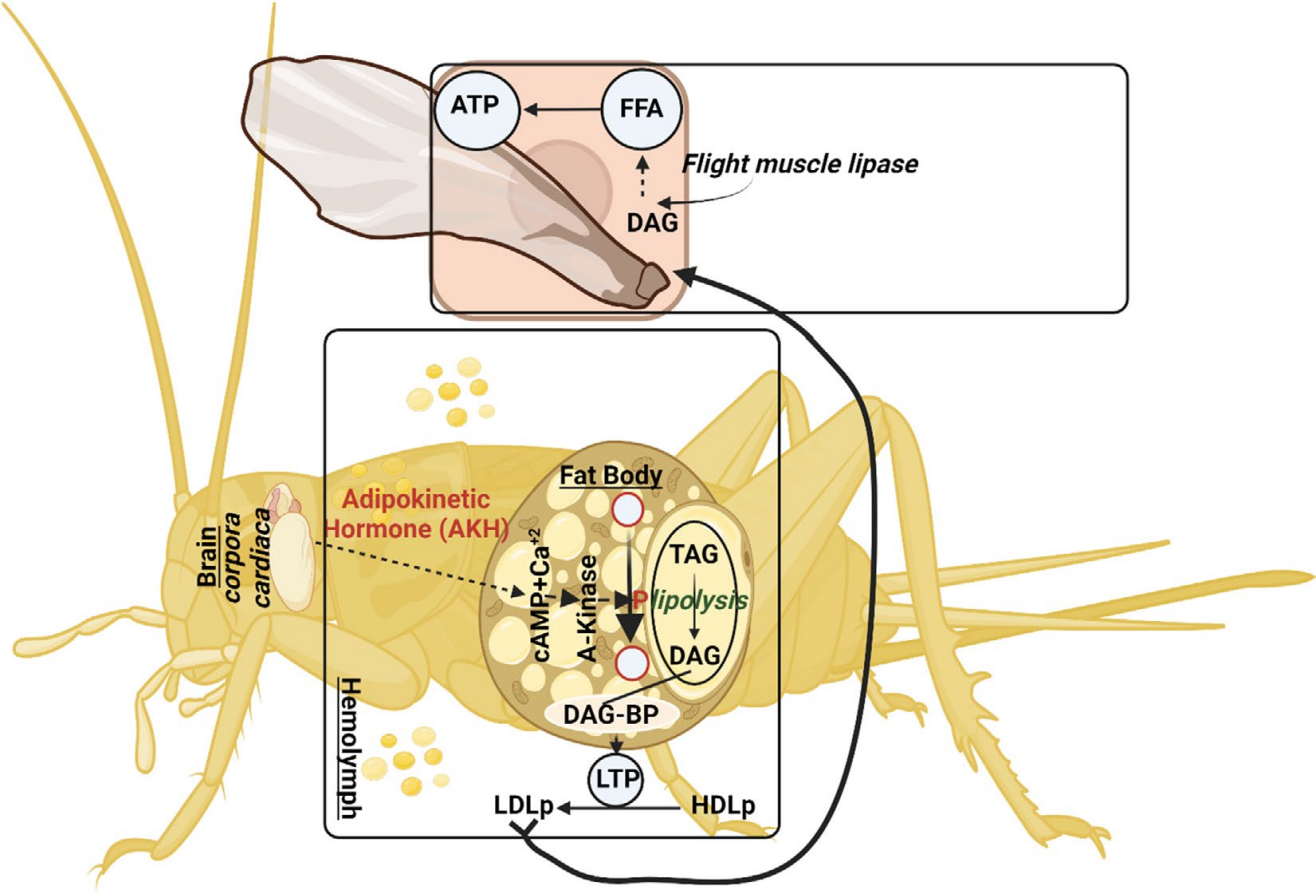
Lipid transport to target sites via lipophorins from fat body to flight muscle. AKH, adipokinetic hormone; ATP, adenosine triphosphate; cAMP, cyclic adenosine monophosphate; CC, corpora cardiaca; DAG, diacylglycerol; FB: fat body; FFA, free fatty acids; HDLp, high‐density lipophorin; LDLp, low‐density lipophorin; TAG, triacylglycerol.

Regardless of the external delivery diacylglycerols (DAGs), the FB maintains constant Triacylglycerol (TAG) stores through de novo synthesis (Figure 8). The FB indeed takes up DAGs from the hemolymph. This transfer to lipophorin Lp is mediated by lipid transfer particle (LTP), which is also indispensable in the transfer of lipids between lipophorin Lp and the adipocytes in the FB (Toprak et al. 2020). Fatty acid transport proteins (FATPs) and FA‐binding proteins (FABPs) are implicated in the FAs cellular uptake and intracellular lipid transport, implicated in different metabolic functions (Toprak et al. 2020). FAs have to be activated by conversion into their CoA‐esters before being transported into mitochondria for β‐oxidation. For FA deployment as an energy supply needed during flight, FABP is activated by adipokinetic hormone AKH (Toprak et al. 2020). The diacylglycerol load into the HDLps turns it into an LDLp (Figure 9). Lps particles, composed of two integral apolipoproteins, are produced by the FB, not by the midgut. Apolipophorin‐I (ApoLpI) and apolipophorin‐II (ApoLpII) are present in an invariable molar ratio of 1:1. Lps are termed according to their density: low‐density lipophorin (LDLp), high‐density lipophorin (HDLp), and very high‐density lipophorin VHDLp (Toprak et al. 2020). Their activity is controlled by the adipokinetic hormone AKH. Released from the FB as an underloaded HDLp, the newly synthesized Lp reaches the midgut to acquire dietary fat. Once DAG is loaded into the HDLps, it returns to the FB as an LDLp, discharges its DAG, then returns to the hemolymph as an HDLp (Figure 9). When HDLp acquires DAGs from the FB, it takes up the apolipoprotein particle apolipophorin‐III (ApoLpIII) that circulates free in the hemolymph. During lipid loading, the ApoLpIII increases the Lpp particle surface. HDLp is the main circulating lipoprotein in the insects hemolymph. In *L. migratoria* and *M. sexta* , which use lipid as energy for flight, apolipophorin‐III (ApoLpIII) is present in the hemolymph of adults as a lipid‐free protein. It is also necessary in cases of starvation (Toprak et al. 2020). LDLp also transfers DAGs to target cells such as epidermal cells, ovaries, cells in flight muscles, and oenocytes that unload the DAGs, hydrolyze them, and oxidize the liberated FAs for energy. The LTP, a very high‐density lipoprotein typically found in the cytoplasm of epithelial cells, stabilizes Lp and facilitates primarily DAG lipid transport from the midgut to the unloaded Lp in the hemolymph. Lps participate in lipid loading in the midgut and unloading in target tissues without being degraded (Toprak et al. 2020).

## 4 Biochemistry of Insect's Fat

The FB usually gathers large quantities of lipids, which vary significantly during insects' life Wrońska et al. (2023). In some insects, it might be more than 50% of the total dry weight (Table 2). Lipid amounts can be high mainly in reproducing female insects; nevertheless, it can be equally high in males (Wrońska et al. 2023). Once emerged, adults enter an intensive period of feeding activity to prepare for sexual maturation by accumulating lipids in the FB (Wrońska et al. 2023). Zhang et al. (2019) found a positive relationship between the reserves of lipids in the FB and the predisposition of insects toward flight behavior. Triacylglycerol (TG) represents more than 90% of total FB lipids, making it the key lipid constituent in most insects (Toprak et al. 2020; Wrońska et al. 2023). The monoacylglycerol (MG), diacylglycerol (DG), and free fatty acids (FFAs) composition varies between insects. If detected, sterols and sterol esters are minor components of total lipids (Toprak et al. 2020; Wrońska et al. 2023). Physiological processes in insects, such as starvation, flight, or egg development, require the mobilization of triacylglycerol (TAG) reserves in the insect FB in a process called lipolysis. This physiological process, carried out by enzymatic hydrolysis, aims to supply diacylglycerol (DAG) to target organs (Toprak et al. 2020).

Physiological processes in insects such as starvation, flight, or development of eggs require the mobilization of triacylglycerol (TAG) reserves in the insect FB (Toprak et al. 2020). This process is called lipolysis, and it is carried out by enzymatic hydrolysis aiming to supply diacylglycerol (DAG) to target organs (Toprak et al. 2020). This lipolytic system, present in the insect FB and activated in response to quick changes in lipid requirements, is called AKH‐signaling‐dependent lipolysis (Toprak et al. 2020). The adipokinetic hormone receptor (AKHR) is found to increase fat storage; the overexpression of AKH instead decreases the lipid content (Gáliková et al. 2015). The triacylglycerol hydrolase activity in the FB is maximum in the adult stage, producing energy from larva‐accumulated lipid reserves that will be later used by adults as energy for reproduction and flight (Toprak et al. 2020). In AKH‐dependent lipolysis, the AKHR in adipocytes releases the prolipolytic AKH signal via cyclic adenosine monophosphate (cAMP) to the protein kinase A (PKA), leading to phosphorylation of Triglyceride Lipase and Lipid Storage Droplet‐1 (Toprak et al. 2020). PKA‐dependent lipolysis in the LDs represents the majority of the lipolytic response induced by AKH (Toprak et al. 2020).

DAG are transferred by Lps to the surface of adipocytes (Toprak et al. 2020). ApoLpI/II, the apolipoprotein component of HDLp existing in important levels in the cytosol and in LDs, interacts with both TGL and LD (Wu et al. 2014; Toprak et al. 2020). Those substances, in a process mediated by Triglyceride Lipase, may reload the DAG from LDs and transport it to the plasma membrane for secretion into haemolymph circulation (Wu et al. 2014; Toprak et al. 2020).

### 4.1 FAs Composition

Most studied FAs of a large edible insect species are listed in the table. This raw data was also reinforced with two heat maps in figure and figure to give a big image of the fat and FAs content of various insects listed in the table.

Good quality FA particularly long chain omega‐3 FAs such as alpha‐linolenic acid and eicosapentaenoic acid are contained in edible insects made of different FA profiles (Jajić et al. 2020). Mealworms contained high quantities of unsaturated FAs, principally linoleic acid, oleic acid, and saturated fatty acids (SFA) (palmitic acid; Lenaerts et al. 2018). Jajić et al. (2019) found that *T. molitor* larvae contain mainly 55.83% of crude protein and 25.19% of crude fat. Paul et al. (2017) found comparable results and concluded that oleic acid (35.83%) and linoleic acid (22.83%) were the major fatty components of lipids from *T. molitor* larvae and *A. domesticus* , respectively, in agreement with Ravzanaadii et al. (2012) and Tzompa‐Sosa et al. (2014). Almost all insects have the capacity to biosynthesize palmitic, stearic, and oleic acids (Jajić et al. 2020). Zielińska et al. (2015) and Mlcek et al. (2019), studying the nutritional composition of mealworm larvae, found that oleic acid was higher at (36.9%–40.86%) followed by linoleic acid (29.68%–30.9%) and palmitic acid (18.0%–18.6%). Jajić et al. (2020) have detected the presence of docosanoic, eicosapentaenoic, and docosahexaenoic acid and, contrary to Paul et al. (2017), did not find eicosanoic, docosanoic, eicosapentaenoic, and docosahexaenoic acid. In research by Ravzanaadii et al. (2012), no eicosapentaenoic and docosahexaenoic acid were reported. Those results from different research highlight the differences in the levels of FAs in the chemical composition of *T. molitor* larvae. The different feeding substrates of insects could be an important reason for these differences.

Mariod (2013), investigated the oil content and FAs from the melon bug *A. vidiuatus* and the sorghum bug *A. pubescens*. The oil content of sorghum bugs was 60% of dry matter while that from melon bugs amounted to 45% of dry matter. Palmitic acids, stearic acids, oleic acids, and linoleic acids were the major FAs in both cases and were similar to cottonseed, peanut, sesame, and sunflower seed oils in terms of SFA and unsaturated FAs (Mariod 2013). The BSF‐derived oil makes a high‐quality biodiesel with a high content of medium‐chain SFAs of 67% (w%) and a low concentration of polyunsaturated fatty acids (PUFAs) of 13% (Lin et al. 2023).

The composition of FA from *A. viduatus* and *A. pubescens* oils is oleic (45.53% and 41.15%), linoleic (4.90% and 35.21%) and palmitic (31.33% and 11.41%) acid, with respectively, 37.9% and 20.5% of SFAs (Mariod 2013). The author concluded that the quantities of SFA and unsaturated FAs from *A. viduatus* and *A. pubescens* oils are similar to those of commonly used oils in Sudan, such as sesame, groundnut, sunflower, and cottonseed.

Insect oils have high oxidative stability as it contains low amounts of PUFAs such as linoleic and linolenic acid. In previous studies, the author found that blending sunflower oil with melon bug oil increased oleic acid content and a decreased linoleic acid, improving the sunflower oil oxidative stability that increased with the increase of the percentage of melon bug oil in mixtures. The author also mentioned that the storage of both melon and sorghum bug oils at 30°C ± 2°C in the dark for 24 months did not affect their FA compositions.

**FIGURE 10:**
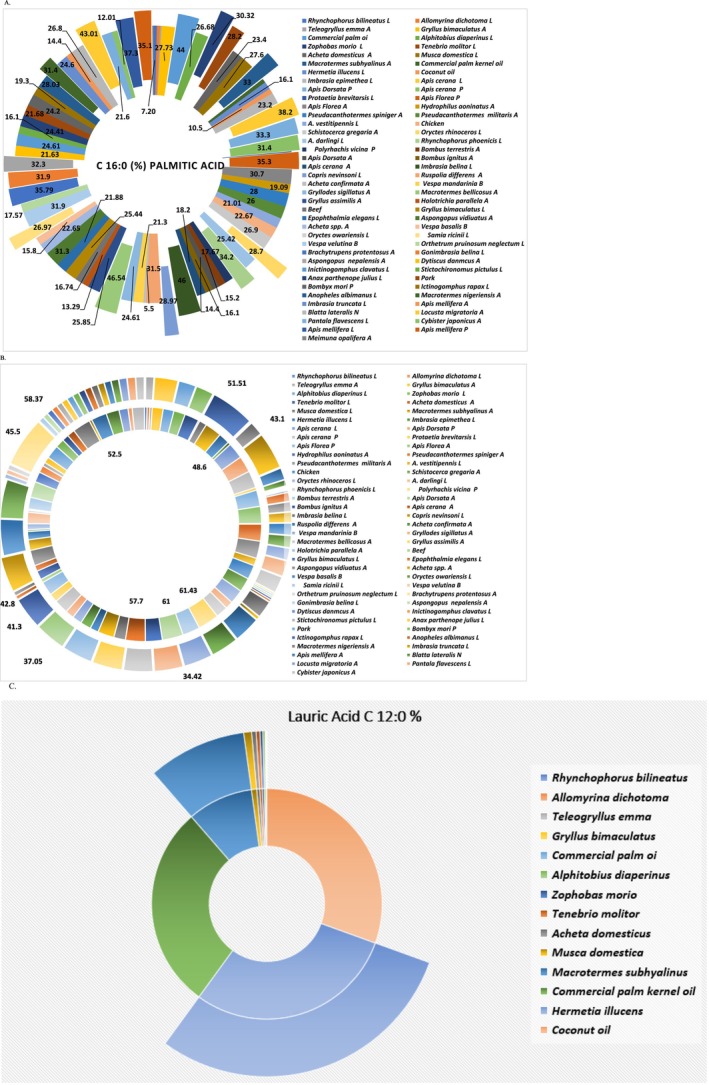
Insect fatty acid: A. C16:0; Oleic acid (C18:1) and Linoleic acid (18:2); C. Lauric acid (C12:0) of insects listed in the table.

**FIGURE 11:**
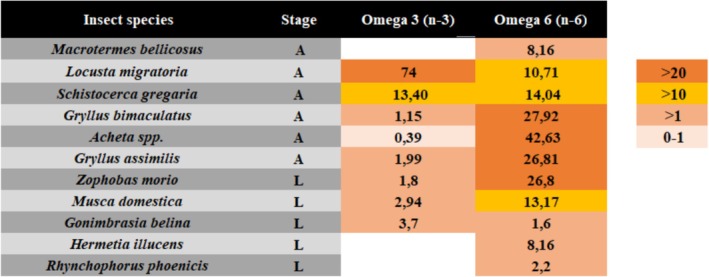
Available data on edible insects' content in Omega 3 and 6 by species and development stage.

FA analysis indicated that palmitic acid (C16:0), oleic acid (C18:1), and linoleic acid (18:2) are in general the most important ones in insect fat/lipid (Table 2). Lauric acid (C12:0) was the major component in BSF FB instead followed by oleic acid and palmitic acid (Table 2; Figure 10). The amount of omega 3 and 6 are shown in the table and the Figure 11. Data shows that *Locusta migratoria* adult present the higher amount of omega 3 while *Acheta* spp. had the higher amount of omega 6. The heist values of omega 6 were shown in all listed Orthoptera species and *Z. morio*. This is in line with Jajić et al. (2020) who found that *T. molitor* larvae lipids display a very high n‐6/n‐3 ratio. *A. viduatus* oils have a n‐6/n‐3 FAs ratio (10.8) while *A. pubescens* (27.5), a value that should be minor than 4.0 as proposed by the UK Department of Health (Mariod 2013).

Edible insects are a source of high quantities of unsaturated FAs (up to 75% of total FA content) even though their FA profile appears to be generally comparable to vegetable oils and animal fats. In fact, mealworms omega‐3 polyunsaturated composition and other FAs are higher compared to pig and cattle and similar to the ones in fish (Lange and Nakamura 2023). Furthermore, terrestrial insects were found to contain high quantities of long‐chain PUFAs compared to aquatic insects (Ohler et al. 2023).

### 4.2 Tocopherols and Sterols

Some edible insects containing a quite large fat amount can be a possible source of minor lipophilic compounds like sterols (cholesterol and phytosterols) and tocopherols in the human diet (Sabolová et al. 2016). Sabolová et al. (2016) characterized the fat composition in tocopherols and sterols from larvae of *Z. morio* and *T. molitor* . The authors found that cholesterol was the predominant sterol in all analyzed samples. Both larvae of *Z. morio* and *T. molitor* contained also a high amount of phytosterols. The authors found no significant impact of different regional origins on sterols composition; the effect of the genus instead was crucial. Storage under different conditions also had no impact on tocopherols composition. In this study, the content of mealworm larvae tocopherols was lower compared to edible oils but with significant nutritional characteristics. Larvae of edible insects might represent a possible good source of cholesterol, vitamin D_3_ isomers, tocopherols, and phytosterols (Sabolová et al. 2016).

Major steroid‐like phytosterols and cholesterol are synthesized in plants and organisms via complex mechanisms. The biosynthesis of bile acids and steroid hormones in the body occurs using cholesterol (Velíšek 2014). Insects, not able to synthesize cholesterol de novo, have to use plant phytosterols such as campesterol, β‐sitosterol, and stigmasterol in order to obtain cholesterol important for the vitamin D3 and steroid hormone (ecdysteroids) synthesis (Mlcek 2019). Cholesterol and 7‐dehydrocholesterol synthesis occur during the synthetic pathway by the adjacent dealkylation on the C‐24 alkyl group of dietary phytosterols (Mlcek 2019).

**FIGURE 12:**
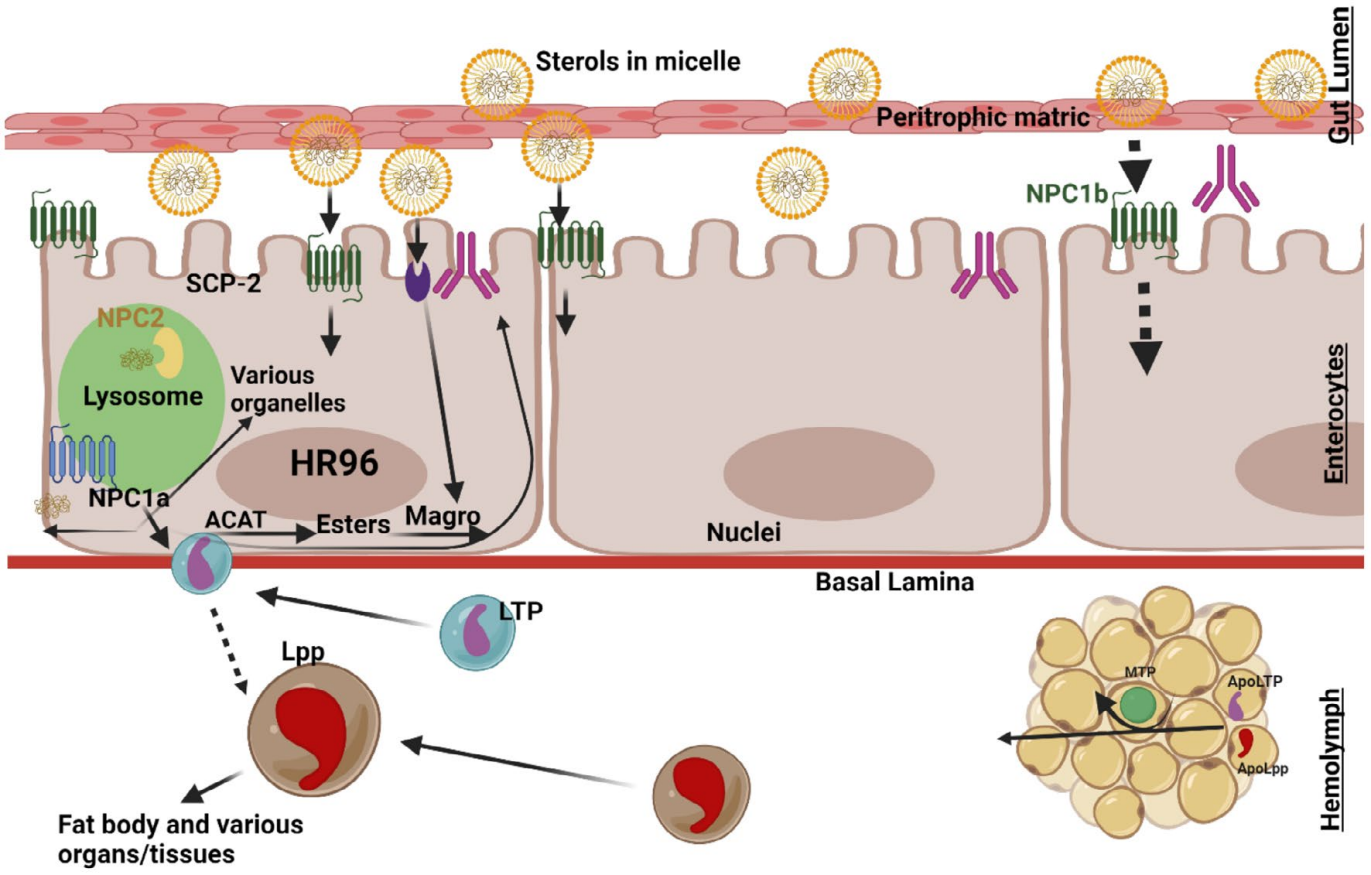
Hypothetical cholesterol uptake, transport, and intracellular trafficking in insects.

The Figure 12 is a graphical depiction of cholesterol transport, uptake, and intracellular trafficking. After foods are ingested, micelles formed contain cholesterol that travels via NPC1b across the peritrophic matrix and through the lipid bilayer or enterocyte membrane to be transported into lysosomes. Within lysosomes, the transfer of cholesterol to membrane‐bound NPC1a is facilitated by NPC2 (Mlcek 2019).

Carried by SCP‐2 proteins, the cholesterol is diffused through the cytoplasm into cells. When inside cells, it can be inserted into the membrane of enterocytes, moved into the endoplasmic reticulum and mitochondrion of various organelles where it can be catalyzed by sterol O‐acyltransferase (ACAT) into cholesteryl esters or can be a membrane structural component. Some cholesterol is excluded via ABC transporters from enterocytes; other, most likely, is transported via Lpp to various organs and tissues. Lpp and LTP, constructed by apoLpp and apoLTP, respectively, are synthesized in the FB. Loading of cholesterol into Lpp occurs through the assistance of LTP. Depending on levels of dietary cholesterol, HR96 instead regulates the cellular homeostasis of cholesterol, coordinating the cellular absorption and reversing transport.

### 4.3 Lipid Implication in Physiological Process

**FIGURE 13:**
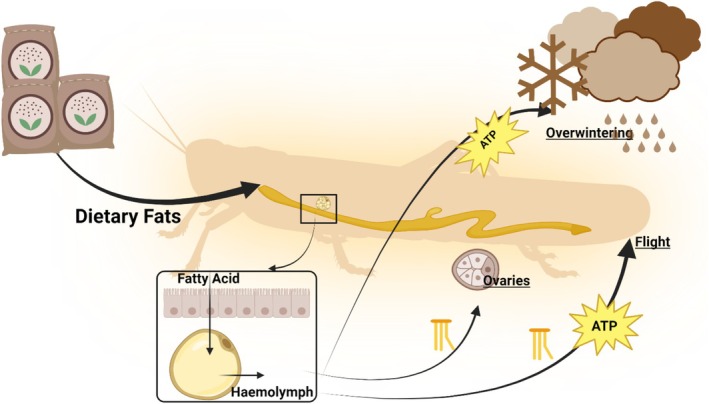
Lipid implication in physiological processes.

Lipid metabolism is fundamental for reproduction, embryogenesis, metamorphosis, flight, starvation, and diapause (Figure 13; Toprak et al. 2020). Ectothermic species like insects face seasonal challenges during the winter due to cold temperatures and food scarcity; in the winter, they fuel their metabolism through their energy stores to increase hemolymph viscosity, likely to reduce lipid transport or meet energetic requirements like reproduction and development (Enriquez and Visser 2023). In fact, in freeze‐tolerant insects, the acetylated triacylglycerols are liquid at low temperatures. Sinclair and Marshall (2018) studied the accumulation, use, and conservation of fat stores in insects overwintering and their selectivity toward fat consumption or conservation and concluded that lipids are vital for overwintering insects. Most insects prepare for winter by lipid accumulation that will play a key role as a source of energetic fuel during overwintering behavior (Enriquez and Visser 2023). In order to prevent the effect of cold shock during extended periods of low temperature, most insects enter into a photoperiod diapause and metabolic suppression (Kaczmarek and Boguś 2021). Lipid stores are crucial for the survival throughout the winter of diapausing insects Through the accumulation of LDs as compensation strategies, insects might prepare for diapause and overwintering. They might either convert TAGs stores to FFAs and transport them to the mitochondria to meet energetic needs, as in the case of Diptera (Culicidae; Kaczmarek and Boguś 2021; Zhang et al. 2019).

The presence of LDs characterizes different fat cells such as trophocytes (Skowronek et al. 2021). Adipocytes are present in highest contact with the circulating hemolymph. Nutrients in the form of glycogen and triglycerides (TAGs) are stored in the FB. Insects store FAs and glycogen during the larval feeding stages, which will serve as energy during nonfeeding periods and for adult life fecundity (Arrese and Soulages 2010). FA are also important for phospholipids, pheromones, and eicosanoids synthesis (Arrese and Soulages 2010). The majority of circulating proteins and metabolites in the hemolymph are produced by the FB (Van De Bor et al. 2021).

**FIGURE 14:**
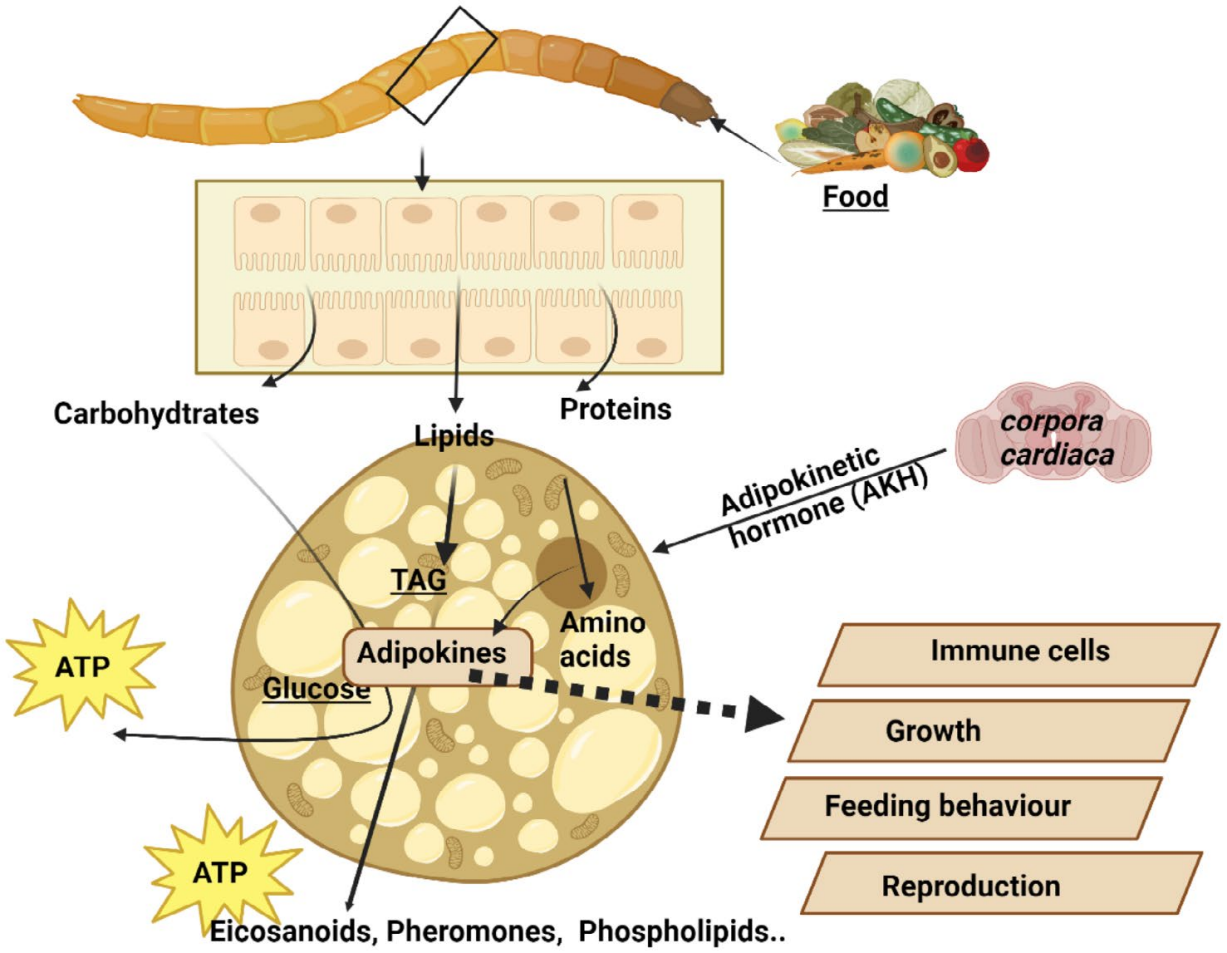
The central role of adipokines in fat cells coordinates fly physiological and developmental processes in insects: Intrinsic neurosecretory cells in the glandular part of the corpora cardiaca CC secrete adipokinetic hormone (AKH). AKH mobilizes lipids or carbohydrates from FB cells in order to produce a variety of adipokines. Furthermore, it synthesizes in the general circulation antimicrobial peptides (AMPs) and releases proteins and metabolites.

The lipid metabolism responds to various factors like the availability of nutrients, the composition of food, and various hormones like the adipokinetic hormone. The FB is a unique organ with great ability to synthesize and store TAG as an energy source. This storage plays a key role through secreted signaling molecules such as the adipokines, particularly during periods of nonfeeding like metamorphosis or prolonged periods of flight (Meschi and Delanoue 2021). Dietary lipids, carbohydrates, and proteins are transferred across the gut epithelium after being hydrolyzed in the midgut. Dietary FA serve to synthesize and accumulate TAGs in a process called lipogenesis (Meschi and Delanoue 2021; Chng et al. 2017). Lipogenesis occurs in the FB; the synthesis of de novo FAs can also happen through acetyl‐CoA. After digestion, FAs are absorbed by enterocytes that are located in the midgut and then converted into diacylglycerol (DAG). Most of them are absorbed by adipocytes and later esterified by diacylglycerol transferase, producing TAGs which will be stored in LDs (Meschi and Delanoue 2021). In the case of high energy demand instead, the FB performs lipolysis, distributing DAG to other tissues (Meschi and Delanoue 2021). The corpora cardiaca (CC), in the retrocerebral complex, contains intrinsic secretory cells producing adipokinetic hormone among others (Chng et al. 2017; Meschi and Delanoue 2021; Figure 14).

The insect FB plays a role in the production of antimicrobial peptides (AMPs) during infection. The energy requirements of the immune system during the immune response affect other physiological processes; this implicates adequate regulation of the metabolic mechanisms because energy is not unlimited. Fat is one of the nutrients supporting and stimulating the immune system; it covers the high energy requirement to activate the immune system (Al‐Esawy 2023).

Furthermore, they provide ω‐3 and ω‐6 oil acids, absorb fat‐soluble vitamins (A, D, E, and K) enhancing permeability and stability for cell membranes (Al‐Esawy 2023).

Cuticular lipids of a grand part of insect species are mainly composed of hydrocarbons; nevertheless, other compounds such as free FAs, alcohols, and esters have been identified (Gołębiowski et al. 2011). Several factors affect the vulnerability or the resistance of insects to fungal attack, such as the important exoskeleton composition, structure, and the efficacy of their defense system. Furthermore, they are involved in different types of chemical communication between species (Gołębiowski et al. 2011; Batalha et al. 2020). Cuticular lipids in insects help to maintain water balance, reduce insecticides and toxins penetration; moreover, they act as a first barrier against parasites, microorganisms, and predators (Gołębiowski et al. 2011). Thus, the determination of the cuticular FA profile is fundamental. Lipid classes such as hydrocarbons, triacylglycerols, and free FAs were detected in extracts from the three insect species: the wax moth *G. mellonella*, the pine‐tree moth *D. pini*, and the blowfly *C. vicina* (Gołebiowski, Maliński, Boguś, et al. 2008; Gołębiowski, Maliński, Nawrot, et al. 2008). Triacylglycerols were identified as the main compounds in G. mellonella and *D. pini* extracts, while FAs dominated all lipids in *C. vicina* extracts. Gołębiowski et al. (2011) also discussed the probable role of cuticular FAs in preventing fungal infection.

Batalha et al. (2020) conducted a study to characterize the cuticle and internal lipid compounds of *Rhynchophorus palmarum* adults male and female by gas chromatography coupled to mass spectrometry (GC–MS) and to assess their antimicrobial activity. The authors identified 10 methyl esters of FAs esters of C14–C23, with only in male cuticle of C22:0, C21:0, and in female C20:2. Cuticular and internal fractions obtained were found to have interesting antimicrobial activity. Extracted lipids resulted in minimum inhibitory concentrations against Gram‐positive bacteria ( *Staphylococcus epidermidis* , *Enterococcus faecalis* ), Gram‐negative ( *Pseudomonas aeruginosa* , *Escherichia coli* , *Klebsiella pneumonia*), and fungal species ( *Candida albicans* , *Candida tropicalis* ). *R. palmarum* 's cuticle could be used in bio insecticidal strategies and a bioactive solution against bacteria and fungi (Batalha et al. 2020).

The Table 3 summarizes the role of lipids and fat bodies in physiological processes:

**TABLE 3:** Role of lipids and fat bodies in physiological processes and biochemical regulation in commonly reared and harvested edible insects.

Insect species	Stage	Physiological process	Biochimical regulation	References
*Hermetia illucens (BSF)*	Larvae	Modulation of lipid metabolism‐related gene expression during larval development	Larvae with a higher weight have a higher percentage of saturated fatty acids and a lower percentage of unsaturated fatty acids (eicosapentaenoic (EPA) and docosahexaenoic acid (DHA))	Giannetto et al. (2020)
*Musca domestica* (Common housefly)	Adult	Low‐density lipoprotein	Take up lipids released from the midgut/fat body cells and transport them to the fat body or to target tissues without endocytosis and lysosomal degradation	Kaczmarek and Boguś (2021)
*Tenebrio molitor* (Mealworm)	—	Reared on the diets based on 100% bread and 100% oat flour showed SFA, PUFA percentages, and an n‐6/n‐3 ratio more suitable for human consumption	Dietary fatty acids satisfied the balance between a fat composition of high quality and favorable growth condition	Dreassi et al. (2017)
*Acheta domesticus* (House Cricket)	Female and male adults	Lower lipid reserves in male and female crickets and the overall higher metabolism	Lower lipid storage in crickets is not directly related with their higher fecundity, but it would be related with their shorter lifespan. Males have relatively shorter lifespan than females	Gutiérrez et al. (2022)
*Gryllus bimaculatus* (Field Cricket)	Adult, fifth instar, nymphs	AKH administration significantly increased the proportion of unsaturated fatty acids (USFAs) to total fatty acids with decrease of the saturated fatty acids (SFAs) in hemolymph	Hemolymph lipid level and composition are modulated by AKH signaling with a complementary feeding behavior toward USFAs	Fukumura et al. (2018)
*Gryllus assimilis* (Jamaican field cricket)	—	The phospholipid fractions oils are rich in phosphatidylcholine (PC), phosphatidylethanolamine (PE), and phosphatidylinositol (PI), which are the main components of lecithin	Cricket oil makes a promising alternative source of lecithin can be an alternative sources for Lecithin, a surfactant used in wide range of food as an antioxidant, flavor protector and emulsifier, typically extracted from crude soybean oil	Tzompa‐Sosa, Dewettinck, Provijn, et al. (2021); Tzompa‐Sosa, Dewettinck, Gellynck, and Schouteten (2021)
*Locusta migratoria* (Migratory locust)		Adipokinetic hormone/corazonin‐related peptide (ACP), facilitate muscle lipid utilization in a famous long‐term migratory flighting L. migratoria	Fatty acid‐binding protein (FABP) mediated the effects of ACP in regulating muscle lipid metabolism during long‐term flight in locusts	Hou et al. (2021)
*Blattela germanica*	—	HDLp high‐density lipoprotein	Acquire fatty acids from midgut or fat body, deliver pheromones to the cuticle and specialized pheromone glands, hydrocarbons to the epicuticle, fat body, and ovaries	Kaczmarek and Boguś (2021)
*Schistocerca gregaria* (Desert locust)	Mature adult	Fatty acid binding protein (FABP) detected in the flight muscle. FABP is absent at the beginning of the adult stage, then is accumulated in muscle cytosol during the first 2 weeks after metamorphosis Fatty acid translocase	The expression of FABP impairs the ability to fly Transport of FFAs to the flight muscle during flight	Kaczmarek and Boguś (2021); Rajapakse et al. (2019)
*Bombyx mori* (Domesticated silkworm)	Female pupae	Ecdysteroids and juvenile hormone controls substance exchange between fat body, hemolymph and ovaries during metamorphosis. Fatty acid translocase	Fat body have a direct effect under “hormonal factors” on the ovary and egg development during metamorphosis. Selective carotenoid transport for cocoon coloration; transport of FFAs in the larvae midgut	Poyraz et al. (2013); Kaczmarek and Boguś (2021)
*Macrotermes bellicosus* (Termites)	—	The fatty acid mixture of the extract is mainly composed of linoleic and oleic acid	Antibacterial activity of *M. bellicosus* samples with a highest growth inhibition of *Staphylococcus aureus*	Hammoud Mahdi et al. (2020)
*Macrotermes subhyalinus* (Termite)	King and queen	The adipocytes of the queen store only a small quantity of reserves. The adipocytes accumulate large amounts of reserve substances	Vitellogenesis	Han and Bordereau (1982)
*Galleria mellonella* *(wax moth)*	Larvae, pupae, adults	Fatty acids C5‐12, C18:3, C20:0 and C20:1 retarded the fungal penetration	Some cuticle fatty acids are implicated in the inhibition of fungal propagule germination	Wrońska et al. (2018)
*Zophobas atratus* (Superworm)	—	Dose of Tenmo‐AKH induces mobility of free fatty acids and other free lipid components AKH influences on carboxylic acids metabolism and their incorporation to major lipids reserves in the fat body.	AKH signaling affect the fatty acid composition of the fat body	Gołębiowski, Cerkowniak, Urbanek, Słocińska, et al. (2014); Gołębiowski, Cerkowniak, Urbanek, Dawgul, et al. (2014)
*Zophobas morio* (Superworm)	Larvae	Present high proportion of lipids. High saturated fatty acid (SFA) and monounsaturated fatty acid (MUFA) content, with palmitic and oleic acid being the most abundant ones. Among polyunsaturated fatty acids (PUFA), the omega‐6 linoleic acid is abundantly found	Larvae are richer in fat than adults	Rumbos and Athanassiou (2021)
*Apis mellifera* (European honey bee)	—	High fat diets	Positive effect on the encapsulation and phenoloxidase—PO activity; Increase in hypopharyngeal gland HPGs enhancing their immunity and physiology	Stabler et al. (2021); Al‐Esawy (2023)

The house crickets were reported as the only species with low levels of the long chain‐3 FA DHA (0.35% of TFA), indicating the insect's ability to synthesize de novo this FA (Tzompa‐Sosa, Dewettinck, Provijn, et al. 2021; Tzompa‐Sosa, Dewettinck, Gellynck, and Schouteten 2021). The author registered the presence of phytosterols, in addition to the lower levels of LDL cholesterol, which reduce the absorption of intestinal cholesterol generally used as food additives (E499), in cricket oil as bioactive compounds that could bring health benefits (Tzompa‐Sosa, Dewettinck, Provijn, et al. 2021; Tzompa‐Sosa, Dewettinck, Gellynck, and Schouteten 2021).

**FIGURE 15:**
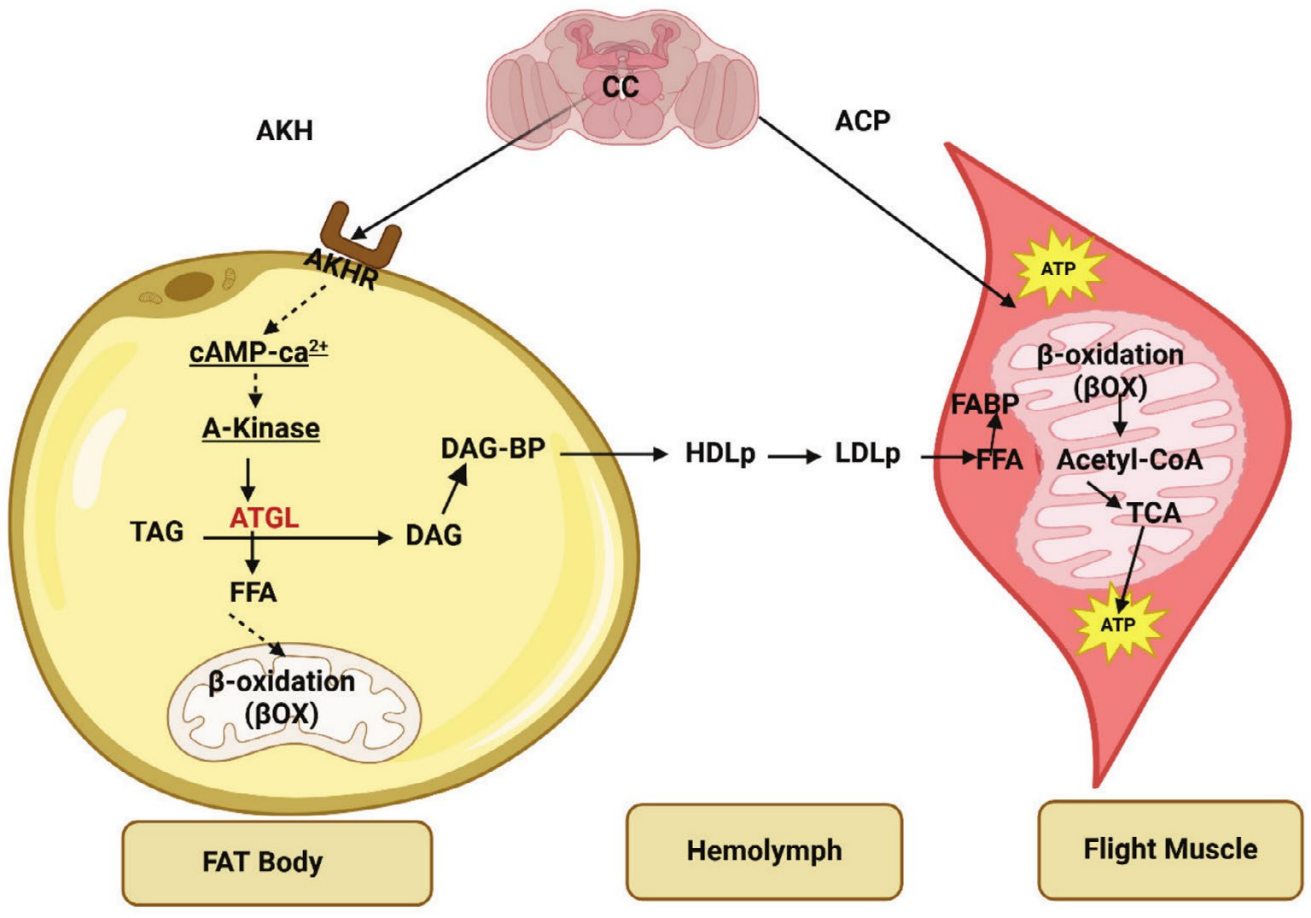
Fatty acid metabolism, including β‐oxidation (βOX) regulation process of energy during insect flight; *CoA* is acetylated to acetyl‐*CoA* by the breakdown of fatty acids through β‐*oxidation*: AKH/corazonin‐related neuropeptide; AKH, adipokinetic hormone; AKHR, AKH receptor; ATP, adenosine triphosphate; cAMP, cyclic adenosine monophosphate; CC, corpora cardiaca; DAG, diacylglycerol; DAG‐BP, DAG binding protein; FABP, fatty acid binding protein; FFA, free fatty acids; HDLp, high‐density lipoprotein; LDLp, low‐density lipoprotein; TAG, triacylglycerol; TCA, tricarboxylic acid cycle.

Lipids also serve as an energy source in long‐term flying insects (Kaczmarek and Boguś 2021). In the initial stages of flight, carbohydrates are generally used as an energy source. During longer flights, fats are generally used as an energy source. During the first few minutes of flight, octopamine, a hormone governing the use of carbohydrates and that increases trehalose concentration in the hemolymph as part of stress responses, induces the first release of DAG from the FB (Kaczmarek and Boguś 2021). The accentuated phase of TAG mobilization, though, happens through the adipokinetic hormone action. The release of AKH from the corpus cardiacum results in the increase of the concentration of DAG, which constitutes the principal fuel for flight in the hemolymph (Kaczmarek and Boguś 2021; Toprak 2020). AKH, thought to act through the AKHR, induces directly the mobilization of lipid in the adipose cells of the FB, releasing DAG from TAG stores (Skowronek et al. 2021; Kaczmarek and Boguś 2021). TAG molecules are then transported to flight muscles through the implication of Lpp, where they are digested to FFAs and oxidized to generate energy (Kaczmarek and Boguś 2021). Hou et al. (2021) have studied the metabolic regulation process of lipid substances during the flight of *L. migratoria* During the first 15 min of incessant flight, glycogen and glucose levels in the flight muscles of *L. migratoria* decrease gradually to a constant level. After 30 min of incessant flight, lipids are gradually accumulated in the hemolymph, and high‐calorie lipids are used as the principal energy supplier (Hou et al. 2021; Li et al. 2023). The metabolic regulation process of the energy substances during insect flight is shown in Figure 15.

Stored triacylglycerol (TAG) in the insect FB during flight is catalyzed by adipose triglyceride lipase (ATGL) in order to produce the main form of lipid transport, diacylglycerol (DAG) (Li et al. 2023). Lipoproteins, formed after diacylglycerol binding to apolipoproteins, are then transported through the hemolymph into the flight muscle (Li et al. 2023). According to the density and size of particles, apolipoproteins can be divided into carrier protein I (apoLp‐I), carrier protein II (apoLp‐II), and carrier protein III (apoLp‐III). Assembled diacylglycerol with apoLp‐I and apoLp‐II forms high‐density lipoprotein (HDLp) while high‐density lipoprotein combined with apoLp‐III is converted into low‐density lipoprotein (LDLp). HDLp is released into the hemolymph, and LDLp releases DAG into the flight muscle, where their decomposition into FFAs and glycerol occurs. The dissociated apoLp‐III from the complex contributes to new DAG transport with high‐density lipoprotein (Li et al. 2023). Insect flight can be related to an important increase in the transcription and expression of the *FABP* gene. After FFAs bind to the FABP in the cytoplasm, they are transported between the cytoplasm and mitochondria. The silenced *FABP* gene expression significantly shortened the flight duration of individuals of the desert locust *Schistocerca gregaria* and *L. migratoria* (Orthoptera: Acrididae), and in the case of *L. migratoria*, the flight distance was also decreased significantly (Hou et al. 2022; Li et al. 2023). The silencing of the *FABP* gene had a serious effect on the FAs oxidative metabolism in the flight muscle during sustained flight. FFAs are catalyzed by fatty acyl‐coenzyme A on the outer mitochondrial membrane to produce fatty acyl A that are next transported by carnitine palmitoyltransferase (CPT) to the mitochondrial matrix in the inner and outer membranes of mitochondria (Li et al. 2023).

Lipids are also involved as sources of energy in oocyte maturation in females and for membrane formation. The mobilization of reserves from the FB to the ovaries is required for egg development, even if oocytes have a reduced ability to synthesize FAs de novo (Arrese and Soulages 2010). The majority of the accumulated lipid in oocytes has its origin from the FB and was later transported to the ovaries via Lpp (Lu et al. 2018; Kaczmarek and Boguś 2021).

The different morphological changes occurring in the growth and reproduction phase in hemimetabolous and Holometabolous affect lipid content. Three developmental stages are included in the life cycle of hemimetabolous insects: egg, several nymph instars, and adult, while Holometabolous insects go through the larva and adult form. Holometabolous are adapted to different ecological niches; larvae and adults therefore do not compete for the same food. (Terra and Ferreira 2020). Early stages of hemimetabolous insects generally have less lipid compared with adults. Periods of energy accumulation are noticed in holometabolous insects during intense periods of growth and development that affect energy storage. The metamorphosis in holometabolous, nonfeeding stage is completely fueled by energy reserves accumulated during larval development, which might explain the higher relative lipid content of holometabolous larvae compared with hemimetabolous larvae (Lease and Wolf 2011; Kaczmarek and Boguś 2021). During the pupal stage, low amounts of internal FFAs are present; this might be connected with the larval FB disintegration in the pupal stage.

Larval feeding is important; nutritional deficiencies during this stage may impact the adult behavior and size, resulting in smaller adults. Moreover, insects may become vulnerable and have a reduced capacity for reproduction (Nestel et al. 2016; Kaczmarek and Boguś 2021). Furthermore, lipid deficits during the larval stage in the Lepidoptera may compromise immune system function and resistance to pathogens (Kaczmarek and Boguś 2021).

## 5 Application of Insect Lipids From Research to Industry

**FIGURE 16:**
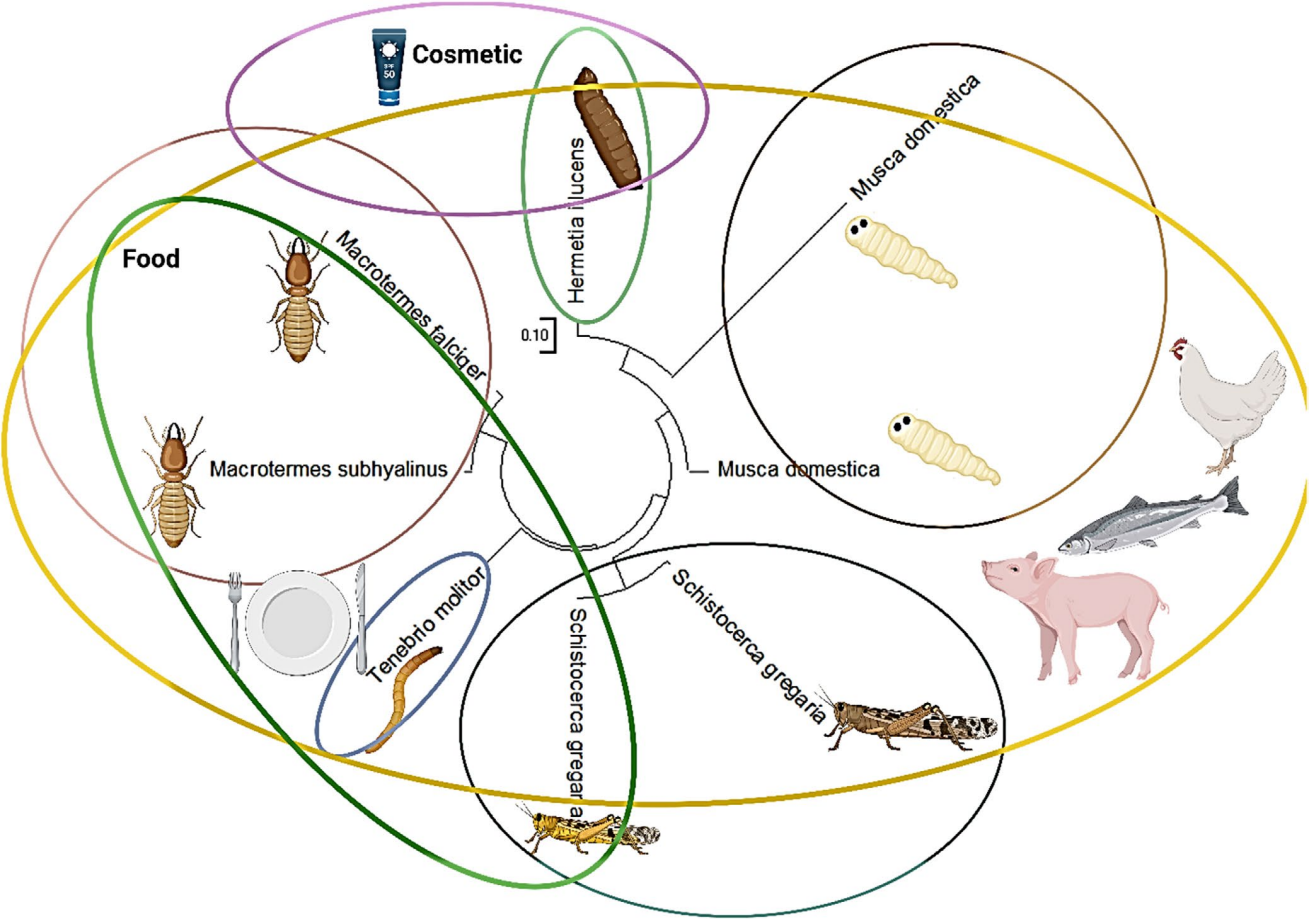
Industry application of insect lipids: Sequences to draw the tree were taken from Genebank (mitochondrion, complete genome). Evolutionary relationships of taxa are shown by the Neighbor‐Joining tree (Saitou and Nei 1987) and conducted in MEGA11 (Tamura et al. 2021).

During daily life, a lot of waste biomass is produced. The inappropriate handling of these organic wastes contaminates the environment and causes energy waste. Sustainable waste management, by recycling organic wastes such as poultry and livestock stool, plant straw, and daily food waste through the bioconversion by some edible insects, helps to reduce energy and nutrition wasting. Reared insects, by recycling organic waste, are a source of proteins and energy for livestock feed, while the insect frass serves as organic fertilizer and green compost suitable for the crop's growth (Figure 16; Jucker et al. 2020; Li et al. 2021; Ragossnig and Ragossnig 2021; Surendra et al. 2016; Zhang et al. 2021; Lin et al. 2023). BSF is a great example of insects able to convert organic waste into valuable products. Nevertheless, bioconversion or waste management by insects should go through rigorous security supervision and quality control measures according to safety risks associated with both human beings and animals (Lin et al. 2023). This section will review the implication of lipids from research to industry.

### 5.1 Biodiesel Production

The worldwide predicted usage of oil and other liquid fuels was about 121 million b/d by 2040 (Mangas‐Sánchez and Adlercreutz 2015; Energy UD 2016; Wang et al. 2017). Global energy consumption will rise by 48% to 815 quadrillions British thermal units (Btu) by 2040 (Mangas‐Sánchez and Adlercreutz 2015; Energy UD 2016; Wang et al. 2017). These concerns, in the contexts of reducing fossil reserves, global warming, energy security, higher oil prices, CO_2_ emissions, and have led to a global policy shift toward the development of sustainable, economic, and energy‐efficient processes and the use of renewable and low‐cost raw materials to generate liquid energy (Tan and Lee 2016; Wang et al. 2017).

**FIGURE 17:**
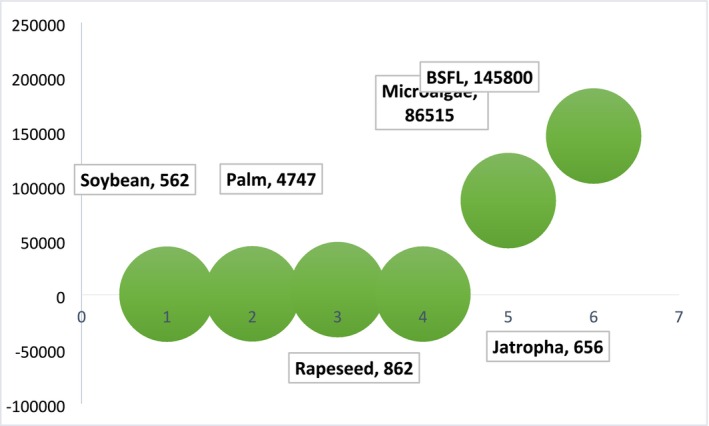
Estimation of Biomass Feedstocks Biodiesel Productivity in kg biodiesel ha^−1^ year^−1^ (Hoekman et al. 2012; Park et al. 2022).

**FIGURE 18:**
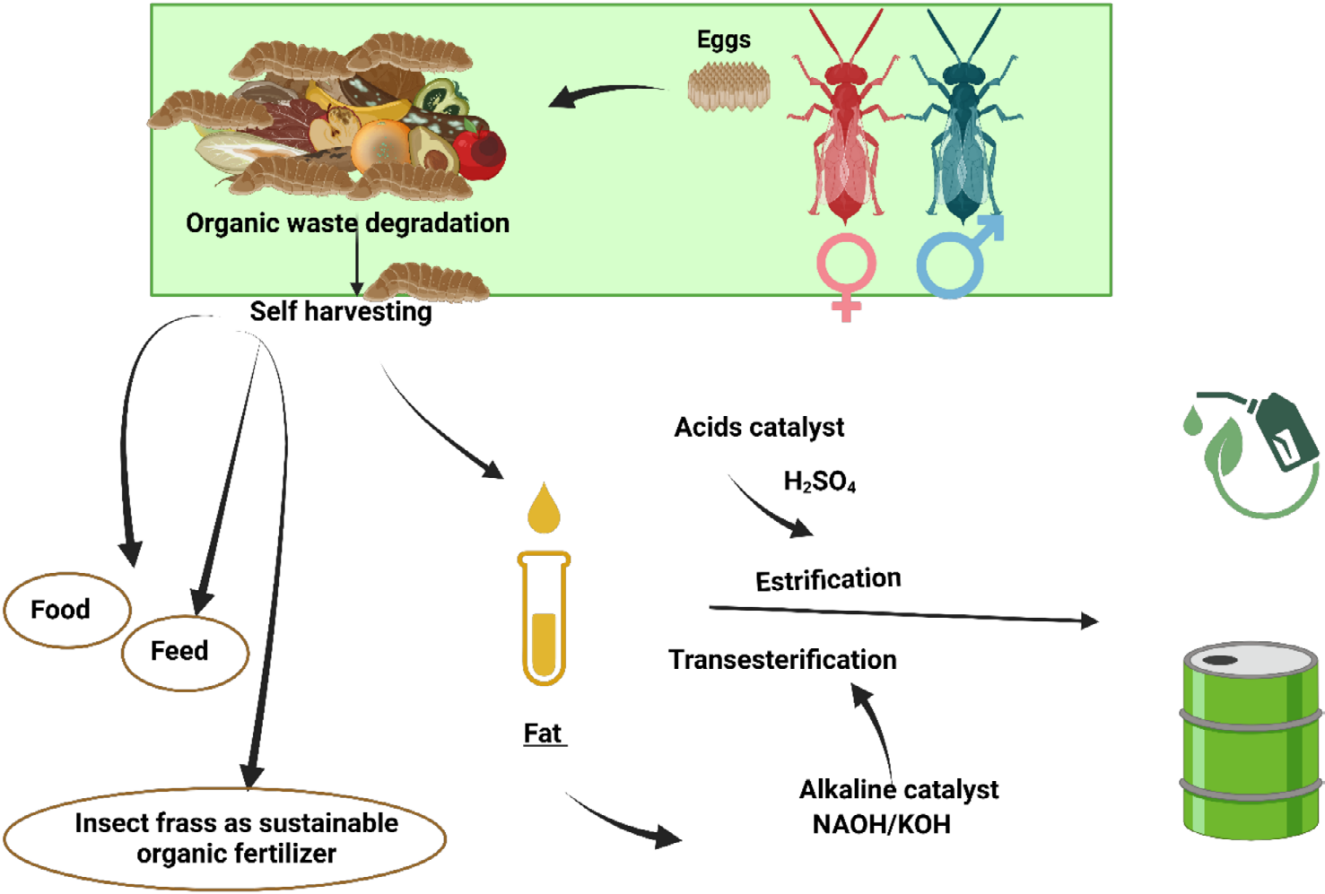
The production of biodiesel from BSFL using transesterification.

Particular attention in many countries has been dedicated to BSFL due to their high fat content (about 35% lipid) as a renewable energy source for biodiesel that is both environmentally friendly and biodegradable (Wong et al. 2019). Scientists developed a biochemical method to use BSFL to turn organic waste into biodiesel. Through a process known as transesterification, which involves the reaction of triglycerides (fat/oil) with alcohol, leading to the formation of esters and glycerol, it is possible to convert the fat/oil extracted from these insects into biodiesel (Figures 17 and 18).

Manzano‐Agugliaro et al. (2012) highlighted a great number of species with an ether extract higher than 25%, reaching even levels close to or above 77% in a study conducted on fat content of insects and their utilization in the production of biodiesel. Manzano‐Agugliaro et al. (2012) found that the fat content varies generally between orders, species, and their stages of development. The authors found that the larval stage had the most accumulated fat. Insect species can be fed with agricultural, industrial, or urban by‐products in order to accumulate a high amount of potentially excellent quality fat, like SFAs C16 and 18 with desirable physical and chemical properties, like kinematic viscosity, calorific value, and oxidation stability, which are conducive for conversion into energy through biodiesel production. Therefore, factors such as insect species, origin (wild or bred in captivity) and type of diet are important to consider for fat targets in the production of biodiesel (Manzano‐Agugliaro et al. 2012).

Fat from insects could be a future promising resource for the production of biodiesel (Lai 2014). The ecologically friendly production of biodiesel from yellow mealworms reared on decayed vegetable material was first studied by Zheng et al. (2013). The yellow mealworm larvae contained 23%–47% fat (Alves, Sanjinez Argandoña, et al. 2016; Veldkamp et al. 2012).

In a study, Ishak and Kamari (2019) investigated the production of biodiesel from BSFL reared on kitchen waste instead of plant‐based biodiesel. Authors reported that the produced biodiesel met the standards established by the American Society for Testing and Materials and the European Standard, in agreement with established quality benchmarks for biodiesel. A pertinent study was led by He et al. (2022) on the production of biodiesel from BSFL using enzymatic transesterification with lipase SMG1 and lipase Eversa transform 2.0 as catalysts. The results of their investigation proved that using a combination of lipase SMG1 and lipase Eversa Transform 2.0 as catalysts compared to the use of a single enzyme led to a significantly higher biodiesel yield.

BSF larvae contain high fat and are promising for biodiesel production. Traditional methods to extract fat from BSFL biomass for biodiesel production require a long time, a high volume of solvent, and specific thermal circumstances. To address such limitations, Su et al. (2019) proposed an enzyme‐assisted extraction method using pretreatment with different proteases prior to the extraction by n‐hexane. Su et al. (2019) suggested that the enzyme‐assisted extraction was efficient for extracting insect fat as potential for use as oil for biodiesel production. The enzyme‐assisted extraction method was revealed by the authors as suitable for use in large‐scale industrial applications.

BSFL, housefly larvae, and yellow mealworm beetle have been previously identified for biodiesel production (Zheng et al. 2013; Yang et al. 2014; Nguyen et al. 2016). BSFL have a short life cycle and a rapid reproduction rate without a negative impact on humans and the environment, which makes it a potential resource for biodiesel production (Makkar et al. 2014; Surendra et al. 2016). BSFL are capable of consuming different organic wastes, such as food waste, lignocellulosic biomass, and animal manure, accumulating fat to produce low‐cost oil feedstock (Li et al. 2011; Zheng et al. 2012; Salomone et al. 2017). BSFL biodiesel properties meet the standards ASTM D6751 and EN 14214 (Nguyen et al. 2018).

Fatty acid methyl ester (FAME) of biodiesel produced from insect larvae via transesterification of lipid has lately gained the interest of researchers, mainly on BSF's sixth instar larvae self‐harvesting nature (BSFL). Wong et al. (2019) harvested the fifth and sixth instar of BSFL reared on coconut endosperm waste (CEW) with the addition of mixed‐bacteria powder to CEW. Wong et al. (2019) found that the fifth instar BSFL had 8% more lipids than the sixth instar lipid content (34%) with predominantly FA methyl ester from all groups set composed of C12:0 (60%), C14:0 (15%), C16:0 (10%), and C18:1 (10%). In terms of FAME content, instead, the produced biodiesel from both instars shows no differences. The FA methyl ester yield from BSFL was higher (38.5%) with modification on raw CEW with mixed‐bacteria powder (0.5 wt%) fermented for 21 days without affecting the development of BSFL. Harvesting BSFL at earlier instars was advantageous and practical as it enhanced the FAME yield from the biomass of BSFL (Wong et al. 2019).

Park et al. (2022) synthesized biodiesel through (trans)esterification of BSFL grown on food waste, achieving both biofuel production from organic waste materials. Authors obtained 86.51% as the highest yield of biodiesel. Biodiesel produced from BSFL satisfied the Korea fuel standard (KS M 2965) in all its parameters except for oxidation stability.

Mariod (2011) studied the melon bug (*A. vidiuatus*) and the sorghum bug (*A. pubescens*). Biodiesel from those oils was transesterified in the presence of sulfuric acid using methanol and ethanol. The majority of the insect oil biodiesel characteristics, including FA esters, met the DIN specifications DIN 51606 for biodiesel (water content, iodine number, phosphorus). It is worth highlighting that the kinematic viscosity values of all samples were much higher than those for biodiesel standards. Mariod (2011) proposed blending those products with other low‐viscosity biodiesels to reduce kinematic viscosity.

### 5.2 Cosmetic and Pharmaceutical Industry

Due to their low toxicity, lipid classes are currently emerging in the pharmaceutical industry as a promising opportunity for drug delivery systems. Verheyen et al. (2018) evaluated fats extracted using petroleum ether and refined from three insect species: *H. illucens* (BSF), *L. migratoria*, and *A. domesticus* , for their possible implication in skin care. After being integrated into a hand cream formulation, fats were compared with generally used oils from other sources. BSF fat was found not to be much appropriate for skin‐care product application as it contains 60% of lauric acid, while fats from cricket and locust were rich in C16 and C18 FA, making them more appropriate in skin‐care products. Phospholipids and FFA levels in the three insect species were relatively high compared with commercial refined oils. Those contents need to be removed by appropriate refining protocols. Furthermore, physical refinement is required to remove color and odor, improving their applicability. Locust and cricket lipids presented a low amount of palmitoleic acid, a good component for better skin penetration. Despite that, they were considered more suitable in cosmetics compared to BSFL, with a FA profile comparable to palm kernel and coconut oil FA profiles better if implicated in shower gels and soaps production (Verheyen et al. 2018). Full toxicological assessment needs to be performed before considering fat from insects, including the potential presence of residual solvents or undesired contaminants like pesticides.

Theoretical antibacterial properties of BSFL lipids might be characterized by the plentiful presence of lauric acid that probably could be transformed into monolaurin, an antibacterial, antiviral, and antiprotozoal glyceride for animals and humans. Lauric acid and monolaurin from BSFL were reported with strong antimicrobial activity by Borrelli et al. (2021).

Recently, Sangduan (2018) patented and internationally marketed a skincare product with purified BSF larval fat with several beneficial characteristics improving skin conditions. Sangduan (2018) described the entire process for the skincare product's preparation from BSFL fats. Extracted fats need to be sterilized then mixed with vitamins in order to have a final product with good stability, appearance, and adsorption property. The other useful molecules that can be found in BSFL and could be applied as novel alternative preservatives in cosmetics as well are anti‐AMPs; small bioactive molecules with strong activity against several microorganisms (Franco et al. 2022).

### 5.3 Chemical Industry

Due to their chemical composition, soap and surfactants are additional opportunities that fat from insects may address (Lorrette and Sanchez 2022; Berezina 2017). Surfactant can be obtained from triacylglycerol through a saponification step. Considering the lower quality of body care using soap from animal fat, as tallowates are principally composed of SFAs, oil from insects may be a good alternative due to its better quality. However, the cosmetic market is completely focused on FAs from plant oils for the production of soaps and surfactants to avoid animal origin in the product, enhancing consumers acceptance.

### 5.4 Feed and Food Industry

High levels of fats and important FAs in edible insects are essential for better human nutrition and as a source of energy. Oils from edible insects could make a good supplement for human consumption as they have lower SFAs than beef and pork meat and contain more UFAs, which are more desirable than SFAs (Kinyuru 2014; Akullo et al. 2018). Stinkbugs and termites were found to be rich in PUFAs (Kinyuru et al. 2013; Musundire et al. 2016). PUFAs can play a crucial role in reducing deficits linked to the lack of α‐linolenic and linoleic acid in human diets with limited access to basic foods with adequate essential FAs in many communities exposed to malnutrition and are in charge of children s and infants' health and development (A. Van Huis 2013; Kinyuru et al. 2015; Anankware et al. 2015; Dobermann et al. 2017; Akullo et al. 2018; Khalil 2018; Hlongwane et al. 2020). Furthermore, PUFAs are found to have a direct effect on the food materials flavor, improving food palatability by allowing the retention of their flavor (Mishyna et al. 2021).

dos Santos et al. (2021) investigated the physicochemical properties of a novel insect oil from larvae of *Speciomerus ruficornis* obtained by artisanal nonsolvent extraction. The authors found that the oil presents low levels of acidity and peroxides as an indication of good quality and conservation standards. The composition of FAs in the oil from larvae of *S. ruficornis* displayed oleic (32.2%), lauric (21.3%), myristic (19.0%), and palmitic (17.6%) as major acid content, a significant amount of polyphenols, and a relevant antioxidant capacity. dos Santos et al. (2021) results highlighted the good quality of oil from larvae of *S. ruficornis* as a solvent‐free source of FAs, showing good thermal behavior and holding notable antioxidant capacity. The authors indicated that diverse technologies can increase the valuable implication of *S. ruficornis* larvae oil to produce a butter‐like product in the food, as an example.

**FIGURE 19:**
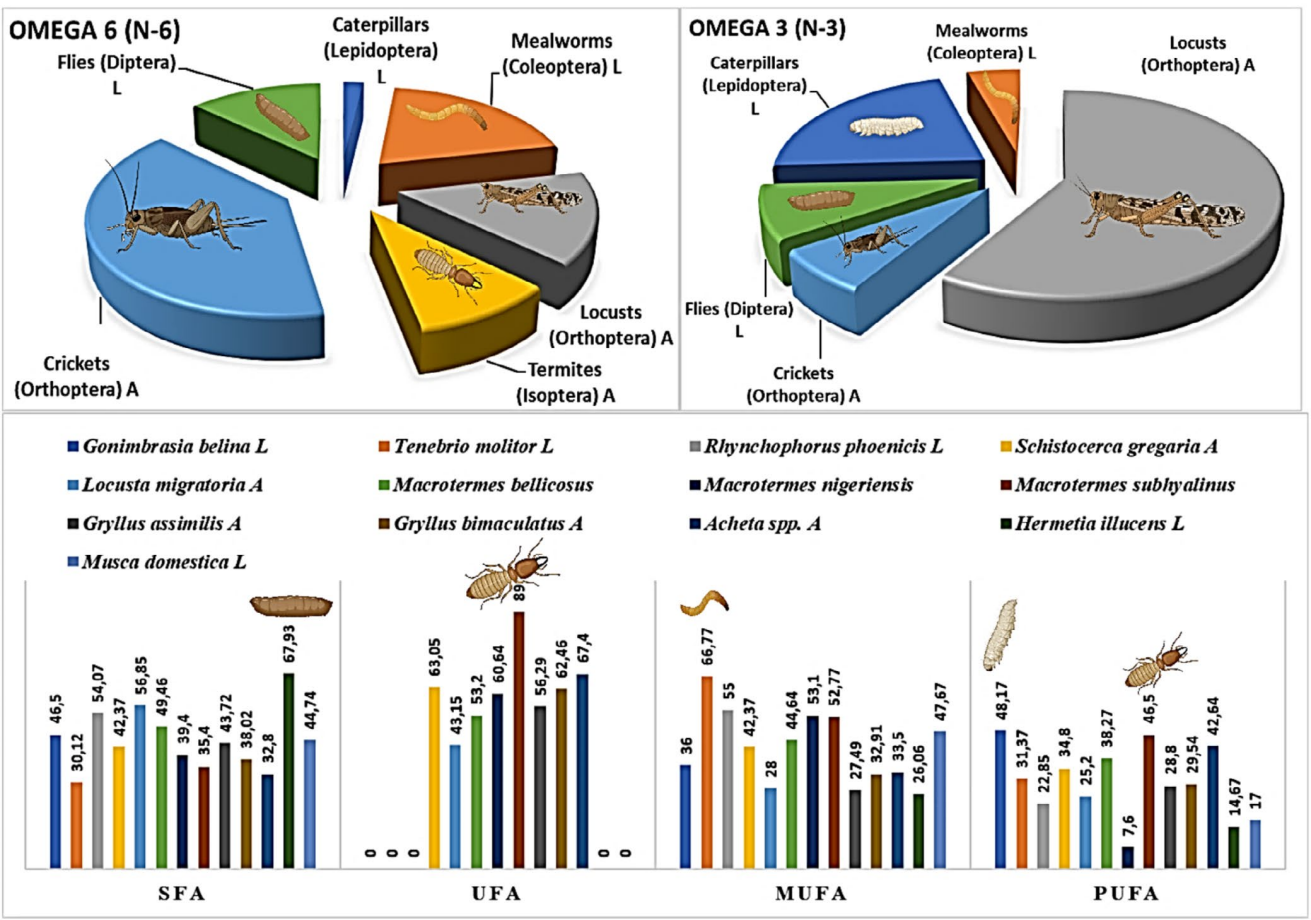
Fatty acid composition (g/100 g) of selected edible insects: A, adult; L, larvae; MUFA, monounsaturated fatty acids; PUFA, polyunsaturated fatty acids; SFA, saturated fatty acids; UFA, unsaturated fatty acids; (Data from Kolobe et al. 2023).

**FIGURE 20:**
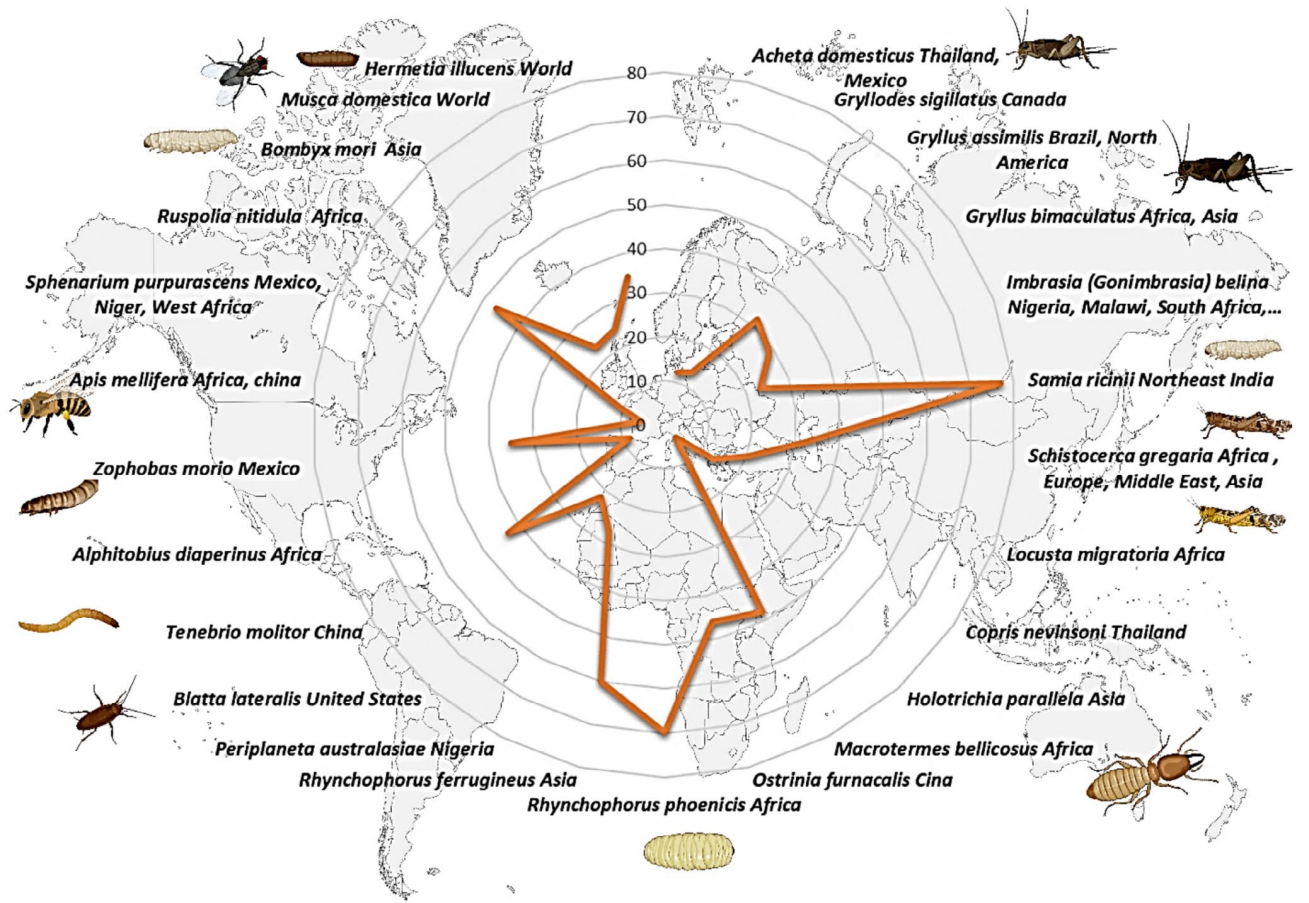
Lipid amount of selected insects distributed worldwide (Data from Table 1).

The Figure 19 shows the amount of SFAs, unsaturated FAs, monounsaturated fatty acids (MUFAs), PUFAs, omega‐3 and omega‐6 in selected edible insects from Kolobe et al. (2023). Larvae of *H. illucens* and *M. domestica* (Diptera order) had the highest amount of SFAs. The reason why insects at early developmental stages exhibit the highest levels of total lipid fraction compared to the adult stage (Mariod 2020) is that soft‐bodied skeletons such as termites had more lipids than those with hard‐body skeletons such as locusts (Dobermann et al. 2017; Kolobe et al. 2023). Termites have the highest amount of unsaturated FAs. The higher amount of MUFAs was recorded for larvae of *T. molitor* , while for PUFAs, larvae of *Gonimbrasia belina* and adults of *Macrotermes subhyalinus* presented high amounts. The Orthoptera order registered the highest amount of omega‐3 and‐6. Desert locust oil was reported to be rich in omega‐3 FAs, vitamin E, and flavonoids compared to major commercial plant oils (Cheseto et al. 2020) making it suitable for baking cookies, taking nonconsumers of insect products a step closer to the entomophagy concept (Cheseto et al. 2020). The amount of lipid and FA contents in different insect species depends on a many factors such as species, sex, development stage, temperature, feed substrates, geographical origin, and extraction methods (Figure 20, Meyer‐Rochow et al. 2021).

Edible insects such as *H. illucens* make an interesting alternative feed resource (protein and fats) to conventional ingredients like fish meal or soybean meal for livestock (Zheng et al. 2012; Li et al. 2016; Loponte et al. 2017; Bovera et al. 2018; Cutrignelli et al. 2018).

The legislation authorized with no restriction's fat from insects as a novel feed ingredient in animal diets. Therefore, its usage is expected on a large scale mainly in aquafeeds. Fats as insect products were previously studied in various literature; in aquaculture, nutrition trials have been performed on Atlantic salmon ( *Salmo salar* ), rainbow trout ( *Oncorhynchus mykiss* ), and Jian carp (*Cyprinus carpio var. Jian*) about the use of *H. illucens* fat as a replacement for rapeseed, fish, and soybean oils (Belghit et al. 2018; Dumas et al. 2018; Li et al. 2016).

Belghit et al. (2018) examined the effect of two sources of *H. illucens* oil (from larvae grown on organic waste streams and on seaweeds) on Atlantic salmon. They were fed rapeseed oil alone or in combination with *H. illucens* meal. The composition of the fish's whole body, dry matter, lipid levels, and crude protein were not affected by the addition of insect oil except for asparagine, alanine, leucine, glutamine, valine, and lysine which were augmented in fish fed with insect lipids (Belghit et al. 2018). Body mineral composition of most amino acids was unaffected by the diet. The use of BSF larvae oil in Atlantic salmon had no negative effect on final body weight (FBW), feed palatability, FCR, protein efficiency ratio (PER), and Fulton's body condition factor K (Belghit et al. 2018). The daily specific growth ratio (SGR) and the growth index (DGI) were contrarily decreased in comparison to the rapeseed and fish oil diet. *H. illucens* oil, in terms of trypsin and bile acid secretion, did not affect the activity of the gastrointestinal tract while it reduced the activity of leucine aminopeptidase in distal intestine tissue. Conclusively, conventional fat sources were successfully replaced by insects' oil and meal in the Atlantic salmon diet.

In a previous study, fish oil was replaced by 2.5%, 5%, and 10% of *H. illucens* larvae fat in the rainbow trout diet (Dumas et al. 2018). The incorporation of 5% of insect oil led to an increase in the composition of whole‐body crude lipids and hydroxyproline without any consequence on fillet composition. *H. illucens* fat used at higher rates (10%) significantly reduced lipid deposition. At the middle dose (5%), the highest value was registered. In terms of intestinal histomorphology and villus length and width, the authors did not observe any significant effect in the anterior as well as posterior intestine. The 10% insect oil incorporation decreased glucose levels in blood plasma analysis and had no effects on other biochemical parameters (e.g., total proteins, creatinine kinase, globulin, albumin, bilirubin, cholesterol). Oil incorporation also did not affect hematocrit and mineral levels (K, Mg, Cl, Ca, P, Na) as well as lipid, dry matter, and the most amino acids apparent digestibility coefficients, except for hydroxyproline, which increased (Dumas et al. 2018).

In juvenile Jian carp ( *C. carpio* var. Jian) diet, Li et al. (2016) replaced soybean oil with 25%–100% of 2.5% of BSF fat prepared with a cold press technique. Authors suggested that fat or flavor from insects could be a nutritional attractant for some fish species, as in the case of sturgeons and common carp (Kasumyan 2018). Herbivorous and carnivorous species such as the common carp ( *C. carpio* ) and its local varieties minimum nutritional request and a large scale of production, particularly in Asia, can generate a high market demand for dietary fats (Benzertiha et al. 2020). Seventy‐five percent and 100% of *H. illucens* oil inclusions reduced the intraperitoneal fat index, decreased adipocyte size in some tissues, while it did not affect whole‐body proximate compositions, hepatopancreas, muscle, and blood serum biochemical indices. All muscle, hepatopancreas, and intraperitoneal fat tissues were supplemented in C12:0, C14:0, and SFA as well as C22:6n3 in muscles. Increasing insect oil incorporation slightly enhanced the total *n3* PUFAs, while reducing *n6* PUFAs in muscles and intraperitoneal fat.

Silkworm chrysalis oil replaced soybean oil with an inclusion level of 3% with a 25%, 50%, 75%, and 100% in the diet of juvenile Jian carp nutrition Chen et al. (2017). A positive effect with 75% and 100% silkworm chrysalis oil inclusion in the diet was reported on the growth performance and the feed utilization by the authors. Furthermore, the omega‐3 PUFAs and the *n3/n6* ratios in the hepatopancreas, muscles, and intraperitoneal fat were significantly increased at a 100% inclusion level of silkworm chrysalis oil in the diet of juvenile Jian carp. Insect oils represent a great potential in sustainable fish nutrition compared to proteins.

Insect oil, from edible insects such as *T. molitor* and *Z. morio*, was found suitable to replace soybean oil in broiler chicken nutrition without compromising their growth performance and nutrient digestibility (Kierończyk et al. 2018).

BSFL contain more than 28% of lipids (Makkar et al. 2014; Wang and Shelomi 2017). The partial or total replacement of soybean oil with BSFL oil altered the FA profile of broiler chickens (Schiavone et al. 2016). FA composition in meats and internal organs is correlated with fat storage and metabolism, and it is related to FA composition in broilers' diet (Taulescu et al. 2010; Khatun et al. 2018).

BSFL oil lowered intraperitoneal fat deposition while increasing omega‐3 FA deposition in muscles in juvenile carp (Li et al. 2016). BSFL oil, similar to coconut oil, the only plant‐origin oil with about 50% of the FA composition, is composed of lauric acid (C12:0) (Dayrit 2014; Li et al. 2016; Ushakova et al. 2016). Medium‐chain FAs, such as lauric acid (C12:0), could be advantageous for the reduction of abdominal fat as they are preferentially used for energy compared to long‐chain SFAs or unsaturated FAs (Wang et al. 2015) in addition, to their antimicrobial effects on gut bacteria (Zeitz et al. 2015; Schiavone et al. 2017). Furthermore, insect oils rich in lauric acid could enhance growth performance and gut health in fast‐growing broiler chickens (Schiavone et al. 2018).

BSFL FA composition, predominantly rich in SFAs such as lauric and palmitic acids, reflects the FA composition of their feed (Makkar et al. 2014; Cullere et al. 2019; Kim, Bang, et al. 2020). Recently, Kim, Kim, et al. (2020) conducted, on broiler chickens, a 30‐day feeding period study to examine growth performance, carcass characteristics, short‐chain FAs, FA composition in abdominal fat, and serum parameters in fed diets containing corn oil, coconut oil, or BSF larvae oil at the level of 50 g per kg of diet. Authors found that the feed conversion ratio was decreased in the coconut and BSFL oil group compared with the corn oil group. Diets containing BSFL oil, rich in lauric and myristic acids, increased contents of SFA in chickens (Kim, Kim, et al. 2020). In 15‐day‐old broilers, it also increased ileal branched‐chain FA and discreetly total short‐chain FA and the cecal propionate at day 30 in the BSFL oil group (Kim, Kim, et al. 2020). Total antioxidant capacity in chickens also increased significantly with BSFL oil compared to corn oil (Kim, Kim, et al. 2020). In corn oil, PUFAs were dominant compared with BSFL oil (Kim, Kim, et al. 2020).

Kim, Kim, et al. (2020) concluded that including BSFL oil in broiler chickens diet improved their feed conversion ratio, serum antioxidant capacity, and increased the incorporation of medium‐chain FAs into adipose tissue of chickens (e.g., abdominal fat pad), breast meat yellowness, and altered intestinal short‐chain FAs. Furthermore, BSFL fats rich in medium‐chain FAs had no adverse effect on organ weights and intestine development (Kim, Kim, et al. 2020).

Kim, Kim, et al. (2020) study suggests that BSFL oil was suitable to improve medium‐chain FAs in edible chickens' tissues. It affects gut health and increases antioxidant capacity in broiler chickens. The inclusion of BSFL oil in broiler diets has been studied; authors reported no negative effects on chickens' growth performance, which makes it a valuable alternative dietary fat source to support productivity (Schiavone et al. 2018; Cullere et al. 2019; Kim, Kim, et al. 2020; Kim, Bang, et al. 2021).

The growth performance and the nutrient digestibility in rabbits were not altered with *H. illucens* larvae fat and *T. molitor* oil as alternative sources of fat. Nevertheless, in the case of supplementation with *H. illucens* fat, which has a high level of SFAs, the meat quality was worsened. Hence, it is recommended to combine the partial supplementation of *H. illucens* fat with vegetable oil or to enrich *H. illucens* larvae fat with n3 PUFAs. This helps to avoid its undesirable effect on the rabbit's meat quality (Benzertiha et al. 2020).

A total replacement with fat sources would be possible without negative effects. More studies on fat inclusion in animal diets are required in order to understand their impact on their physiology and nutrition and for future ì application in the feed industry, taking advantage of their health benefits and their ability to modify FA composition and properties.

The knowledge about the economic viability of insect farming is an emerging question. Insect farming can offer many benefits, like high‐quality animal feed and food. It helps to create jobs and reduce poverty, beside its role in environmental sustainability. Munthali et al. (2023) examined the economic feasibility of BSF farming for animal and fish feed in Malawi as a promising insect species for a sustainable and innovative alternative protein source in animal feed. The study assessed the financial feasibility of BSF farming at various scales of production using Cost–Benefit Analysis. The study results indicate that small‐scale farmers could generate over $2500 annually, while commercial farmers would generate significantly more. The sensitivity analysis established profitability even with variations in production costs and market prices (Munthali et al. 2023).

Waitha et al. (2022) evaluated the growth performance and cost–benefit analysis of improved indigenous chicken fed with different inclusion levels of BSFL meal. The study found that the diet significantly affected the average daily feed intake, weight gain of the chicks, and feed conversion ratio, while the cost–benefit ratio, gross profit margin, and return on investment of feeding chicks with soldier fly larvae (BSFL) meal varied in a significant way. BSFL meal could replace fish meal up to 20% without compromising growth performance, leading to a cost–benefit ratio of 2.12, demonstrating its economic viability Waitha et al. (2022).

Additionally, a recent review by Debbarma et al. (2025) on integrating BSF larvae meal and algal biomass in aquaculture highlights the potential of BSF larvae as a sustainable protein and lipid source which offers cost savings and improves growth in aquaculture species.

## 6 Safety of Fat and Oil From Insects

Since food material with high levels of SFAs such as palmitic acid and stearic acid is considered not suitable for consumption, the best inclusion levels of insects as a source of fats and FAs in food have to be considered. Astrup et al. (2020) highlighted that different SFAs have also different biological effects that are further modified by the food matrix and the carbohydrate content of the diet. Indeed, several foods relatively rich in SFAs, such as dark chocolate, whole‐fat dairy, and unprocessed meat, are not associated with increased cardiovascular diseases or diabetes risk. SFAs and unsaturated FAs of fats and oils have an effect on general health depending on their content, not straightforwardly the sum of the effects of the individual lipid components (Astrup et al. 2020). It rather depends on the interrelating effects from naturally occurring components, overlooked in the assessment of health effects of oils and fats, and from unhealthy compounds introduced by food processing (Astrup et al. 2020). Saturated fats were always a key part of the ancient human diet (Astrup et al. 2020). Stearic acid (C18:0) from dark chocolate has a neutral effect on cardiovascular disease risk. Dark chocolate indeed has been reported to have multiple beneficial health effects such as antihypertensive, antiatherogenic, and possible anti‐inflammatory, antithrombotic, and antioxidative properties. Furthermore, dark chocolate has preventive effects against cardiovascular diseases and type 2 diabetes. Unprocessed red meat, for example, if consumed in modest amounts, is a major source of protein, minerals, bioavailable vitamins, and iron (Astrup et al. 2020). Under Regulation (EU) No 2015/2283, insect food products marketed for human consumption must be first approved as a Novel Food. This authorization first requires a scientific safety assessment by the EFSA. On 13 January 2021, the EFSA published a risk assessment for the yellow mealworm to be placed as food on the market. According to this positive safety assessment, mealworm larvae, whole dried insects, and powder are safe for human consumption. Other insects were found suitable for human consumption since the mealworm was approved. On November 12, 2021, the migratory locust, *L. migratoria*, was approved as novel food by the European Commission. On January 5, 2023, the larva of the grain mold beetle, *A. diaperinus* , was added to the novel food list (Commission Implementing Regulation (EU) 2023/58 of January 5, 2023). In January 2023, the use of partially defatted powder of the domestic cricket, *A. domesticus* , which was already authorized in frozen, dried, and powdered form in February 2022, was also authorized for another applicant (Commission Implementing Regulation (EU) 2023/5 of January 3, 2023). The placing of the insects on the market to the applicants is limited to 5 years within the EU or is only allowed with their authorization. As novel food, insect ingredients must be well labeled and clearly indicated in the list of ingredients to avoid allergic reactions that may occur in individuals who are already allergic to cross‐reactions to mites or crustaceans. Additionally, larvae' diet contamination may be a source of additional allergenic ingredients in the novel foods; therefore, it is necessary to further study the allergenic potential of insects.

As Novel Food products, nevertheless, fat and oil from insects are very similar to some plant oils; equal requirements are expected to justify their safety, harmlessness, and innocuity. Though their high‐quality composition, their safety aspects, and oxidative stability will help for possible future authorization. Oil derived from insects, like any product from other biological sources, feed and food ingredients may contain microbiological and chemical hazards (e.g., bacteria, fungi, mycotoxins, heavy metals). The presence of these hazards has to be assessed for safety concerns in order to obtain regulatory approval and to be able to commercialize the product. EFSA has suggested a regulatory approach to studies of Toxicity as a required parameter of novel food assessment integrating chronic and subchronic toxicity, genotoxicity, carcinogenicity, repeated dose toxicity testing, developmental, and reproductive toxicity (EFSA 2016).

The approach of evaluating the safety of BSFL oil was highlighted by Freel et al. 2021 in dogs for a 28‐day study. Three insect oil inclusion levels were used in the study: 1%, 2.5%, and 5%. For the duration of the trial, blood parameters, as indicative of canine health, were found in normal ranges. No significant differences were observed in body weight, food consumption, and stool quality between treated groups and the control group. The authors concluded that throughout the study and up to an inclusion of 5% of BSFL oil, the general health of dogs was preserved without any safety concerns. Under the study conditions conducted by Freel et al. (2021), trials on dogs demonstrated that BSFL oil was well tolerated and accepted; body weight, stool quality, and very high levels of total tract digestibility of amino acids and macronutrients were preserved along with blood parameters indicating their nutritional status and health.

BSF oil was reported as a high‐energy feed ingredient for nursery pigs in research performed by Heugten et al. (2019). Heugten et al. (2019) ported that BSF oil increased body weight in nursery pigs without affecting hematological and serological parameters (e.g., Triglycerides, glucose, serum concentrations of total protein, and diverse blood cell counts) when compared to corn oil treatment. BSF oil supplementation significantly increased only serum cholesterol and platelet count, while all remaining tested parameters were in the expected ranges (Figure; Heugten et al. 2019).

**FIGURE 21:**
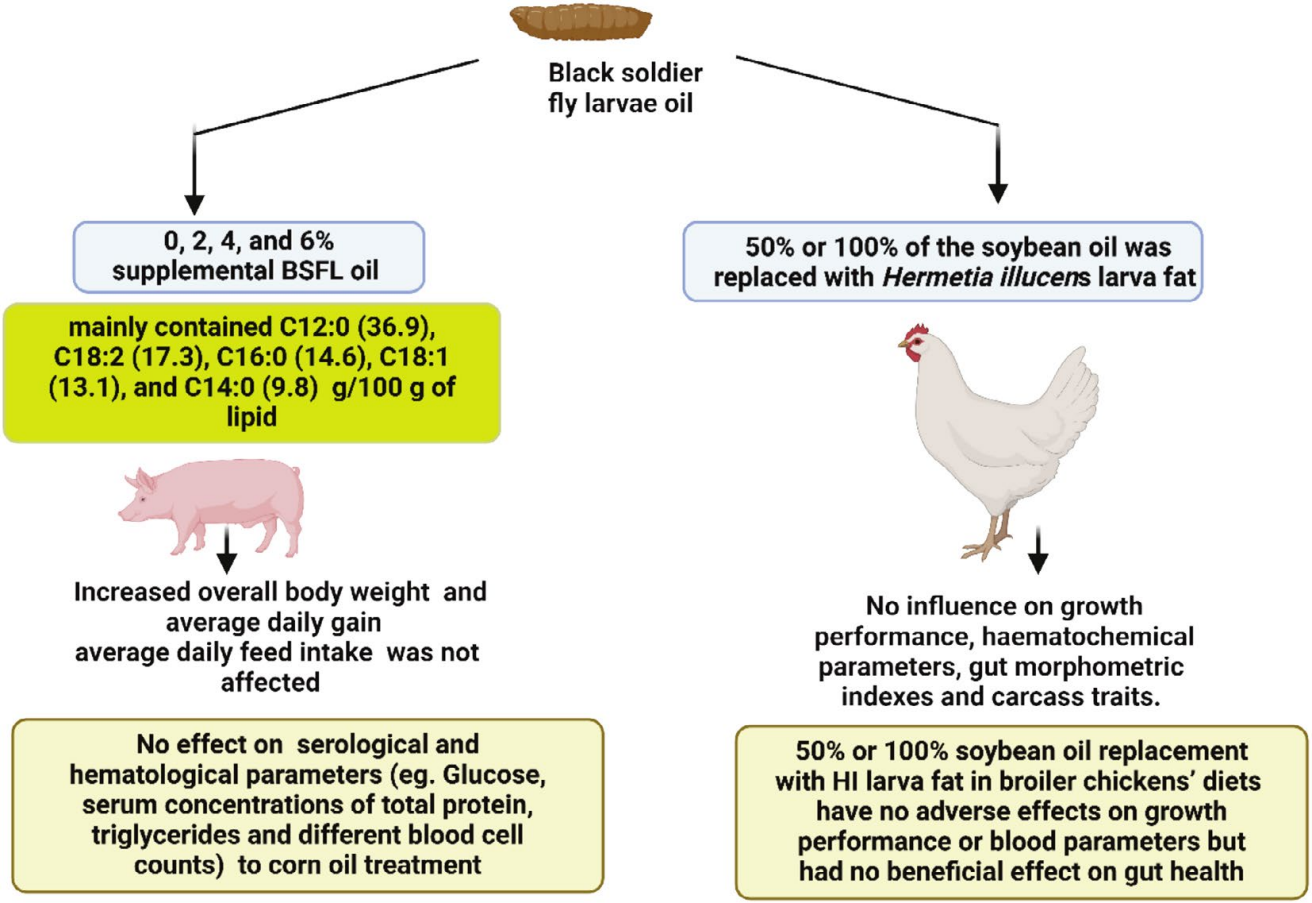
Effect of BSF supplementation in feed.

Schiavone et al. (2018) assessed the effects of partial or total replacement of finisher diet soybean oil with BSF larva fat on the growth performance, carcass traits, blood parameters, intestinal morphology, and histological features of broiler chickens. To the control diet made of soybean oil, either 50% or 100% was replaced with *H. illucens* larva fat ( *H. illucens* 50 and *H. illucens* 100 group, respectively). Authors found the dietary inclusion of *H. illucens* larva fat had no influence on growth performance, haematochemical parameters, gut morphometric indexes, and carcass traits. Schiavone et al. (2018) concluded that 50% or 100% soybean oil replacement with *H. illucens* larva fat in broiler chickens' diets has no adverse effects on growth performance or blood parameters, with no advantageous consequence on gut health (Figure 21).

Studies to determine the safe doses for human consumption of edible insect lipids are necessary. In a first work on lipid toxicity, Alves et al. (2019) investigated *T. molitor* and *P. nucleorum* oil acute toxicity (single oral administration) and subacute toxicity (28 consecutive daily administrations) using rats' models. The treated rats demonstrated a considerable reduction in glucose and cholesterol levels. *T. molitor* and *P. nucleorum* oil did not cause any lethality nor have they made changes in hematological parameters. Therefore, acute and subacute toxicology experiments may be an indicator of the low toxicity of *T. molitor* and *P. nucleorum* oil (Alves et al. 2019).

Food allergies affect about 2%–4% of the adult population and up to 9% of children, representing an important public health problem according g to EFSA. So far, 14 allergens are identified and need to be labeled according to EU rules on food. People with food allergies need detailed informations on products ingredients (Hossain et al. 2023).

The consumption of the insect proteins according to EFSA evaluation would probably lead to allergic reactions in particular cases on subjects with pre‐existing allergies to crustaceans, dust mites, and in some cases mollusks. Allergen reactions may also be related to allergens from the feed consumed by insects (e.g., gluten; Hossain et al. 2023). All those issues are clarified in the authorization of this novel food, which lays down specific labeling requirements regarding allergenicity (Hossain et al. 2023).

Insect lipid products have been used efficaciously as a source of nutrients, energy, and FAs in animal feeds. For example, lauric acid and monolaurin in BSF larvae oil as functional compounds are potential functional ingredients in feed formulations (Hossain et al. 2023). As a source of oil and essential FAs, edible insects could play a very significant role in many processes. The quantity and nutritional quality of insect lipid, a by‐product of insect meal production, depend on target species, life stages, the feeding substrate, and the extraction process (Hossain et al. 2023).

Most research on the effects of insect product inclusion in aquafeed focuses on insect meal as a protein source. Few studies focus on insect oil/lipid as a lipid source in feed. Insect lipids may safely substitute traditional energy sources in fish nutrition without negatively affecting feed utilization performance and growth. Insect lipids are found to improve fillet quality by raising the amount of long‐chain PUFAs in fish fillets (Hossain et al. 2023). Lipids from insects stimulate the immune system and manipulate the gut microbiota with a positive impact on the general health of fish (Hossain et al. 2023).

Previous studies indicated the sufficient amount of unsaturated FAs from edible insects that might meet the necessities of both humans and animals (Seni 2017; Tao and Li 2018; Feng et al. 2018; Kim et al. 2019). Insect consumption by farmed animals might have an indirect effect on human health (Nyangena et al. 2020). Edible insects are considered a suitable substitute to obtain PUFAs; their inclusion in livestock diets could improve poultry meat quality by enriching the meat with different concentrations of essential FAs (Selaledi et al. 2020; Mishyna et al. 2021; Żuk‐Gołaszewska et al. 2022). According to Elahi et al. (2022), the inclusion of 15% BSF meal enhanced MUFA levels while lowering a little the PUFA content of broiler chicken breast meat. The oil extracted from insects might be directly added into livestock diets, supplying PUFAs not available in other oil sources such as sunflower oil and soya full fat (Longvah et al. 2011; Monter‐Miranda et al. 2018). High accumulation of fats in meat leads to the oxidation of lipids and, as a consequence, an uninvited color and taste with reduced shelf life (Kinyuru et al. 2015). Thus, to help balance lipids and FA contents in animal diets, fat‐rich ingredients such as edible insects would be suitable to include.

Allergens and contaminants constitute the major insect safety hazards based on different species, growing substrate, and processing conditions (FAO 2021; Murefu et al. 2019). Food allergies may cause life‐threatening reactions. They affect approximately 5% of adults (Downs et al. 2016). Cross‐reactivity of related proteins such as allergies to shellfish (crustaceans) can cause allergic reactions when consuming edible insects, which are related to arginine kinase groups, α‐amylase, and tropomyosin groups in insects (Fels‐Klerx et al. 2018; Barre et al. 2018). Appropriate food processing methods, such as thermal processing and enzymatic hydrolysis, are crucial to diminish cross‐reactivity and the risk of allergies in edible insects (Pali‐Schöll et al. 2019). With 25%, allergens are identified as main hazards in the Asian continent (Murefu et al. 2019). Between 1980 and 2007, the fourth most commonly reported allergenic source after pineapple, turtle, and crab in China was insects (Lin et al. 2023). During that period, multiple cases of anaphylactic shock were caused by insects (27 by grasshoppers, 27 by locusts, 5 by silkworm pupae, 1 by Clanis bilineata tsingtauica, 1 by cicadas, 1 by bee larva and 1 by bee pupae; Lin et al. 2023). In Thailand, the consumption of fried insects was related to one food poisoning incident. Grasshoppers and pupae might be sources of histamine poisoning (Chomchai and Chomchai 2018).

Other than their nutritional advantage, the potential danger of insects' implication in animal feeds has also been reported because of the accumulation of heavy metals and toxins when insects were grown on contaminated substrates (Wang and Shelomi 2017; Kim, Kim, et al. 2020). The accumulation of heavy metals in insects is a threat to the safety of insect consumption, as for example the accumulation of arsenic in yellow mealworm and cadmium in BSF (Biancarosa et al. 2018; Fels‐Klerx et al. 2016; Lin et al. 2023). Crickets have been found to accumulate heavy metals in feeding trials (Bednarska et al. 2015; Diener et al. 2015). The exposure to low concentrations of heavy metals can be a reason for human death as a consequence of heavy metals toxic effects other than interfering with human metabolomics (Alengebawy et al. 2021; Rai et al. 2019). The regulated breeding of edible insects may reduce heavy metal accumulation in insects (Lin et al. 2023). The threat of pesticides may also present itself as insects can accumulate them through consumption of plants sprayed with insecticide (Imathiu 2020). A serious quality control of the contaminants level in the substrate would help to reduce the presence of most chemical contaminants in insects (EFSA 2015). The potential for zoonotic infections, instead, is considered very low as human viruses cannot replicate within insects. However, intensive rearing of edible insects could be a reason for virus spread, leading to significant losses, and as a consequence, it could increase other health issues, such as resistance to microbial drugs, zoonotic diseases, and insect‐specific viruses (Lange and Nakamura 2021). Proper hygiene and effective heat treatment can significantly reduce transmission of pathogenic bacteria as a risk to human health (EFSA 2015; Lin et al. 2023).

On the safety of common edible insects in China, where more people consume them and the Chinese Ministry of Health recognized the silkworm chrysalis as common food and the Guangxi Zhuang Autonomous Region promulgated a local food safety standard for edible frozen fresh silkworm pupae (DBS45/030–2016), still most edible insects are out of the authoritative list and the negligence of potential insects still needs to be addressed (Gao et al. 2018; Lin et al. 2023). In China, based on food toxicological evaluations including genotoxicity tests, acute toxicity tests, and 28‐day oral toxicity tests, insects usually consumed were found safe (Gao et al. 2018). Procedures for toxicological assessment of food would provide valid methods for assessment and determination of food safety indicators of insects (Feng et al. 2018).

Some insects may also contain antinutrients, such as oxalic acid, tannin, phytic acid and hydrogen cyanide that bind to nutrients maybe acting as chelators resulting in absorption disorders and affecting the bioavailability of nutrients in insects (Lin et al. 2023). Chitin found in honey bees, a well‐known edible insect to the Chinese, seems to have ‘antinutritional’ properties that lower the quality of insect proteins. Chitin as indigestible may diminish the digestion and absorption of closely proteins associated with it (Lin et al. 2023).

Currently, research on the safety of insects is still limited, while there is great interest in the insect's nutritional value. As reported above, there should be a significant concern and more attention for food safety in longer‐term research leading to a robust regulatory framework that rigorously standardizes the feeding, processing, and storage of edible insects. Food safety rules governing conventional foods should extend to insect‐based processing Lin et al. (2023). It is also crucial to establish a system to test for the safety of insects. Various contaminants affecting edible insects' lipid safety associated with insect or fat/oil consumption can be reduced by controlling insect farming and production.

## 7 Health Benefits and Medicinal Uses of Oil and Fat From Insects

The Table 4 summarizes the effect of oil/fat from different insects cited in the literature. Mlcek et al. (2019) analyzed the mealworm profile of sterols and FAs ( *T. molitor* ) and superworm (*Z. morio*), with great future interest in human nutrition. Determined cholesterol content for the mealworm (1335 mg/kg in dry matter) was lower than for superworm (3224 mg/kg kg in dry matter). Stigmasterol and β‐sitosterol were higher in superworm (stigmasterol—44 mg/kg DM and β‐sitosterol—414 mg/kg DM) than in mealworm (stigmasterol—18 mg/kg DM and β‐sitosterol—171 mg/kg DM; Mlcek et al. 2019). Negligible quantities of cholecalciferol were recorded for both species (190–199 μg/kg DM). Atherogenic index (AI), thrombogenic index (TI), and cholesterol index (CSI) were considered by authors as predictors of cardiovascular diseases. Compared to dried beef, a potential benefit of both species could be the balanced proportion of sterols of animal and plant origin. They are nutritionally well‐accessible, and consuming lower weight of dry matter can cover the daily dose of linoleic acid (Mlcek et al. 2019). Very high intake of cholesterol causes high levels of low‐density lipoprotein (LDL) and very low‐density lipoprotein (VLDL), resulting in a major risk of the development of the metabolic syndrome (cardiovascular disease, diabetes mellitus, obesity; Mlcek et al. 2019). The cholesterol is produced in the body through endosynthesis; therefore, the daily intake of 0.15–0.3 g per day is considered optimal, which is much lower than cholesterol amount in a common diet (0.6–0.8 g). A correct diet consuming fiber, phytosterols, and antioxidants would probably reduce cholesterol by 10% (Mlcek et al. 2019).

**TABLE 4:** Effect of lipid/fat extracted from insects on humans/animals.

Insect species	Stage	Fat/oil	Effect	References
*Hermetia illucens* (*BSF*)	Larvae	Fat	Antibacterial feed ingredients with a positive influence on the rabbit cecal microbiota	Dabbou et al. (2020)
*Tenebrio molitor* (Mealworm)	Larvae	Oil	MWO promoted skin wound repair by collagen synthesis, re‐epithelialization and angiogenesis in an in vivo excisional wound model	Kim, Kim, and Chung (2021)
*Acheta domesticus* (House Cricket)	Adult	—	Contain high concentrations of alpha‐linolenic acid (ALA) and linoleic acid	Stull and Weir (2023)
*Locusta migratoria* (Migratory locust)	Adult	Fat	Locust fats are rich in C16 and C18 fatty acids which making them more suitable in a skin‐care products	Verheyen et al. (2018)
*Chorthippus parallelus* (meadow grasshopper)	Adult		High concentrations linoleic acid. alpha‐linolenic acid (ALA) was the most plentiful fatty acid found in this insect. Higher concentration of unsaturated fatty acids compared with saturated fatty acids	Stull and Weir (2023)
*Conocephalus discolor* (edible stinkbug)	Adult		High concentrations of alpha‐linolenic acid (ALA) and linoleic acid	Stull and Weir (2023)
*Rhynchophorus phoenicis* (African Palm Weevil)	Larvae	Oil	PUFA/SFA ratios of 16.70 associated with desirable levels of cholesterol and reduced coronary heart diseases.	Womeni et al. (2009)
*Schistocerca gregaria* (Desert locust)	Adult	Oil	Low Omega‐6/omega‐3 fatty acids ratio; suitable for the reduction of the risk of a number of chronic diseases	Lakshimi and Kavitha (2023)
*Bombyx mori* (Domesticated silkworm)	Chrysalis	Oil	Rich in n‐3 a‐linolenic acid, that allowed to suggest that silkworm chrysalis oil can improve hyperlipidaemia and hyperglycaemia	Mentang et al. (2011)
*Gonimbrasia* sp. (Mopane worm)			Palmitic acid and linolenic acid are the two dominant fatty acids of Imbrasia oil. The ratio of PUFA/SFA is 1.01	Womeni et al. (2009)
*Zophobas morio* (Superworm)	Larvae	—	To cover the daily need for linoleic acid, up to 9 times less dry matter of superworm is needed in comparing to beef	Mlcek et al. (2019)
*Pachymerus nucleorum* (Beetle)	Larvae	Oil	Antioxidant activity (24.3 uM Trolox/g) and high level of unsaturated fatty acids	Alves, Sanjinez Argandoña, et al. (2016); Alves, Sanjinez‐Argandoña, et al. (2016)

The TI for superworm (1.4) can be compared to the fat of beef or pork or to vegetable margarine but the TI of mealworm is similar to that of chicken meat fat (Stajić et al. 2011). The AI of the profiles reported by various authors can be compared with polyunsaturated acids of margarines, and similar to that of olive oil (Mlcek et al. 2019) which makes the consumption of the mealworm more favorable to prevent the risk of cardiovascular disease (Mlcek et al. 2019). Mlcek et al. (2019) also determined the ratio of n‐3 and n‐6 FAs, significant factor describing the risk of the metabolic syndrome arising from fat consumption, also as for the values calculated by other authors it was higher than WHO recommendations (1:2–1:6). The amount of dry matter from mealworms required to cover the daily need for linoleic acid per day was estimated at 205 g and at 708 g for linolenic acid. The amount of dry matter required in the case of *Z. morio* instead is 116 g for linoleic acid and 554 g for linolenic acid (Mlcek et al. 2019). Linoleic acid and linolenic acid contents for *T. molitor* were lower than these for *Z. morio* (Mlcek et al. 2019). Those values were higher comparing to dried meat content of linoleic acid, therefore, according to Mlcek et al. (2019) to cover the daily need for linoleic acid, up to nine times less dry matter of superworm is needed in comparing to beef. Less dry meat linoleic acid is needed than mealworm or super worm dry matter instead.

The food potential of lowering cholesterol is generally indicated by ratio of PUFA/SFA. A PUFA/SFA ratio of 0.2 has been associated with high cholesterol level and high risk of coronary heart disorders. A high P/S ratio instead such as 0.8 is associated with desirable levels of cholesterol and reduced coronary heart diseases (Womeni et al. 2009). Womeni et al. (2009), found high PUFA/SFA ratios for Raphia weevil (*R. phoenicis*), Cricket and Grasshopper of 16.70, 105.75, and 24.94, respectively. Those values suggest the potential these insects or larva if used in the dietetic management of certain coronary heart diseases. The insufficiency of essential FAs caused mainly by tropical vegetable oils could be compensated by the consumption these edible insects would (Womeni et al. 2009). Insects Oil is rich in PUFA, easy to undergo oxidation, therefore heat treatments during cooking of the insects must be accorded a lot of attention and since these insects are not consumed fresh.

The dietary recommendation about reducing the intake of SFAs has to take into consideration specific FAs and food sources as mentioned by Astrup et al. (2020). Astrup et al. (2020). Such recommendations dissuade from other more effective food‐based recommendations, causing a reduction in the intake of nutrient‐dense foods such as dark chocolate, dairy, and unprocessed meat that may help to decrease the risk of type 2 diabetes, cardiovascular diseases, and other diseases, and lead to malnutrition, frailty, and deficiency diseases mainly among groups “at‐risk” (Astrup et al. 2020). Future research on fat or oil from insects should consider the types of FAs, the diverse insects' composition of SFAs, that may possess harmful, neutral, or even beneficial effects in relation to major health outcomes as recommended by Astrup et al. (2020) for food recommendations generally. Astrup et al. (2020) strongly recommended reconsidering the guidelines on the reduction in total SFAs and how food‐based explanations on how to achieve a healthy diet. Research has to focus on the effect of consuming unprocessed or gently processed insects that are more likely to be a crucial factor. Further informations about the health effects of specific process contaminants should be examined in order to minimize their levels (Astrup et al. 2020). According to Astrup et al. (2020), the long‐standing bias about foods rich in saturated fats should be updated with a view toward recommending healthy foods.

Insect lipids may safely replace traditional energy sources in fish nutrition without negatively affecting feed utilization performance and growth. Insect lipids are found to improve fillet quality by raising the amount of long‐chain PSFAs in fish fillets (Hossain et al. 2023). Lipids from insects stimulate the immune system and manipulate the gut microbiota with a positive impact on the general health of fish (Hossain et al. 2023).

**FIGURE 22:**
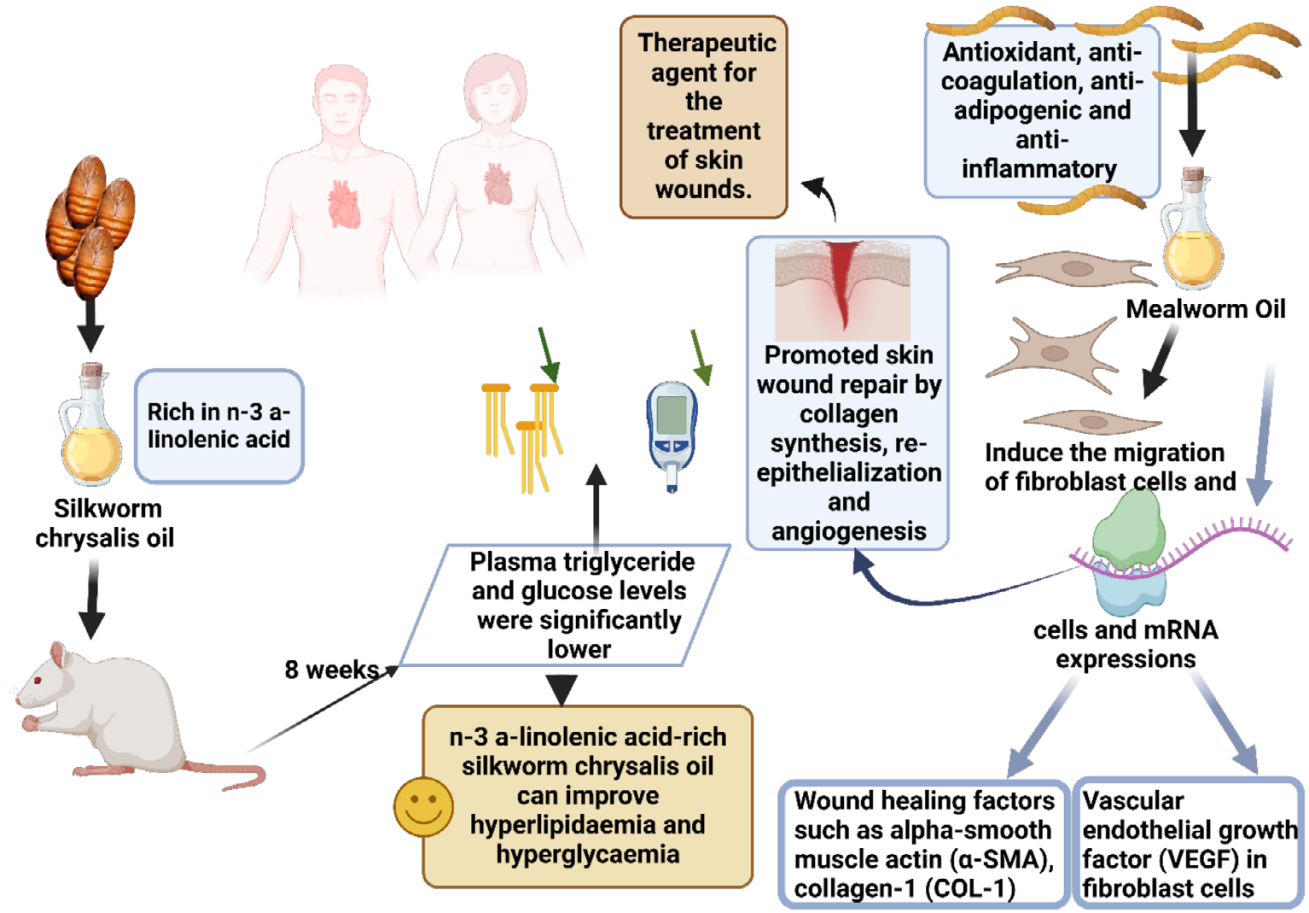
Health benefits and medicinal uses of oil and fat from insects (Kim, Kim, and Chung 2021; Mentang et al. 2011).

A recent article suggested that insect's oil, particularly *T. molitor* oil, could be considered as a therapeutic agent for the treatment of skin wounds (Figure 22). *T. molitor* oil significantly increased the re‐epithelization after skin injury (Kim, Bang, et al. 2021). The authors suggest that *T. molitor* oil positively affects the role of interactions among keratinocytes, fibroblasts, and myofibroblasts. Recent research shows that the mealworm has effects on the antioxidant, anticoagulation, antiadipogenic, and anti‐inflammatory activities (Son et al. 2019; Pyo et al. 2020; Seo et al. 2017).

A skincare product produced using purified fat from BSF larva, patented by Sangduan (2018) and marketed internationally, demonstrated different beneficial characteristics that enhance skin conditions, including smoothing, revitalizing, moisturizing, and tightening the skin. In his work, Sangduan (2018) described the entire preparation method necessary for the skincare products using BSFL fats. Extracted fats from BSF larva (solvent extraction, a screw press, or SC‐CO_2_ extraction) must be separated by filtration or centrifugation from the rest of the mixture. Extracted fats have to be sterilized and then mixed with specific ingredients like vitamins. The final product would have good stability, appearance, and adsorption property (Sangduan 2018).

Purified oil extracted from edible insects could be supplemented to human diets as cooking oil or processed into a by‐product (margarine) as a substitute for vegetable oils rich in n‐3 and n‐6 FAs (Smetana et al. 2020; Cheseto et al. 2020). Several authors highlighted the dietary inclusion of edible insects in human diets improving the livelihood of many countries under risk of malnutrition and reported the implication of essential FAs in the health and development of children and infants (Dobermann et al. 2017; A. Van Huis 2013; Anankware et al. 2015; Khalil 2018; Akullo et al. 2018; Hlongwane et al. 2020). Tao and Li (2018) reported lipids from edible insects as likely to improve dietary energy intake in human and animal diets. Cheseto et al. (2020) compared the chemistry of the oils from the desert locust *S. gregaria* and the African bush‐cricket *Ruspolia differens* with those of olive and sesame oils. Authors also compared the proximate composition of cookies prepared from the oils in order to identify the potential volatiles and FAs contributing to the aroma and taste. Cheseto et al. (2020) results showed that the insect oils were richer in omega‐3 FAs, flavonoids, and vitamin E compared to plant oils. Volatile chemistry showed that alterations in aroma and taste of the cookies were associated with their sources of oils. Consumers' acceptance was high for cookies prepared with *R. differens* (95%) and sesame (89%) oils compared to those with olive and *S. gregaria* oils. In aroma and taste, cookies prepared with insect oils had more than 50% dislike (Cheseto et al. 2020).

Insects contain from 43% to 79% of USFAs, which is near that in poultry and fish. Among unsaturated FAs, the level of PSFAs in insects, consisting of linoleic acid, arachidonic acid, and eicosapentaenoic acid, was found to be higher compared to poultry and fish (Lin et al. 2023). USFAs generally exhibit great benefits for the human body. For a total FA content of 60%, MUFAs are composed of palmitoleic acid and oleic acid (Lin et al. 2023). Yi (2015) found that insects have minor cholesterol content compared with food usually consumed. The cholesterol levels in locusts were found to be comparable to beef and pork but lower than in eggs (Nowakowski et al. 2022; Lin et al. 2023). MUFAs have widely recognized beneficial effects on blood lipid profile, blood pressure, glycemic control, and insulin sensitivity. A diet enriched with MUFAs reduces the prothrombotic environment by altering platelet adhesion, coagulation, and fibrinolysis (Twining et al. 2018).

It has already been demonstrated that oil and fat from insects may have positive effects on immune functions and cecal microbiota in animals such as fish or rabbits (Hender et al. 2021; Dabbou et al. 2020). Dabbou et al. (2020) evaluated in vitro the antimicrobial activities of two types of insect fats extracted from BSFL ( *H. illucens* L.) and yellow mealworm larvae ( *T. molitor* L.). Authors confirmed that *H. illucens* and *T. molitor* fats had a potential antibacterial effect with a positive impact on the rabbit cecal microbiota, which supports the possibility of including *H. illucens* and *T. molitor* fats in their diets.

Usually, insects have higher fat content ranging from 2% to 62% with a FA profile similar to vegetable oils and animal fats and rich in unsaturated FAs (Williams et al. 2016; Tzompa‐Sosa and Fogliano 2017). Particularly, adult locusts (*S. gregaria*), house crickets ( *A. domesticus* ) and deep‐water rose shrimps ( *Parapenaeus longirostris* ) have around 65% of unsaturated FAs (Tzompa‐Sosa et al. 2014; Zielińska et al. 2015). About 75%–76% of total FAs in whole larvae of yellow mealworms are unsaturated FAs (Tzompa‐Sosa et al. 2014) while a high ratio of SFAs (51%–68%) is characteristic of BSF larvae grown on feeding media with the inclusion of the brown algae (Liland et al. 2017).

Insect oil could be used for the cosmetic industry as a lotion or hand cream to moisturize dry skin (Verheyen et al. 2018).

**FIGURE 23:**
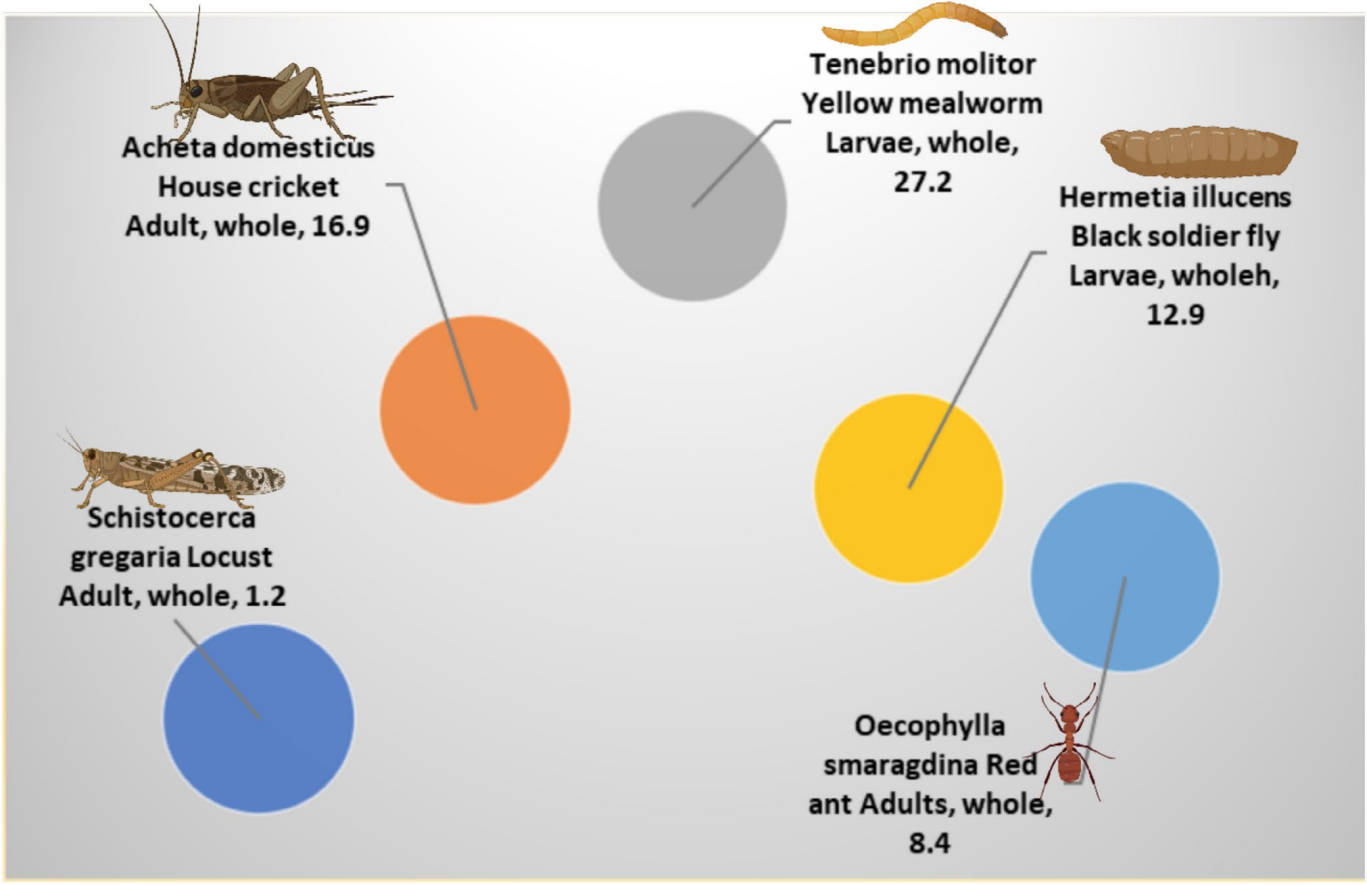
n6/n3 ratio in *Schistocerca gregaria*, *Acheta domesticus* , *Tenebrio molitor* , *Hermetia illucens* , and *Oecophylla smaragdina* (Data from Mishyna and Glumac 2021; values are based on dry weight, fresh weight, methods of lipid extraction).

**FIGURE 24:**
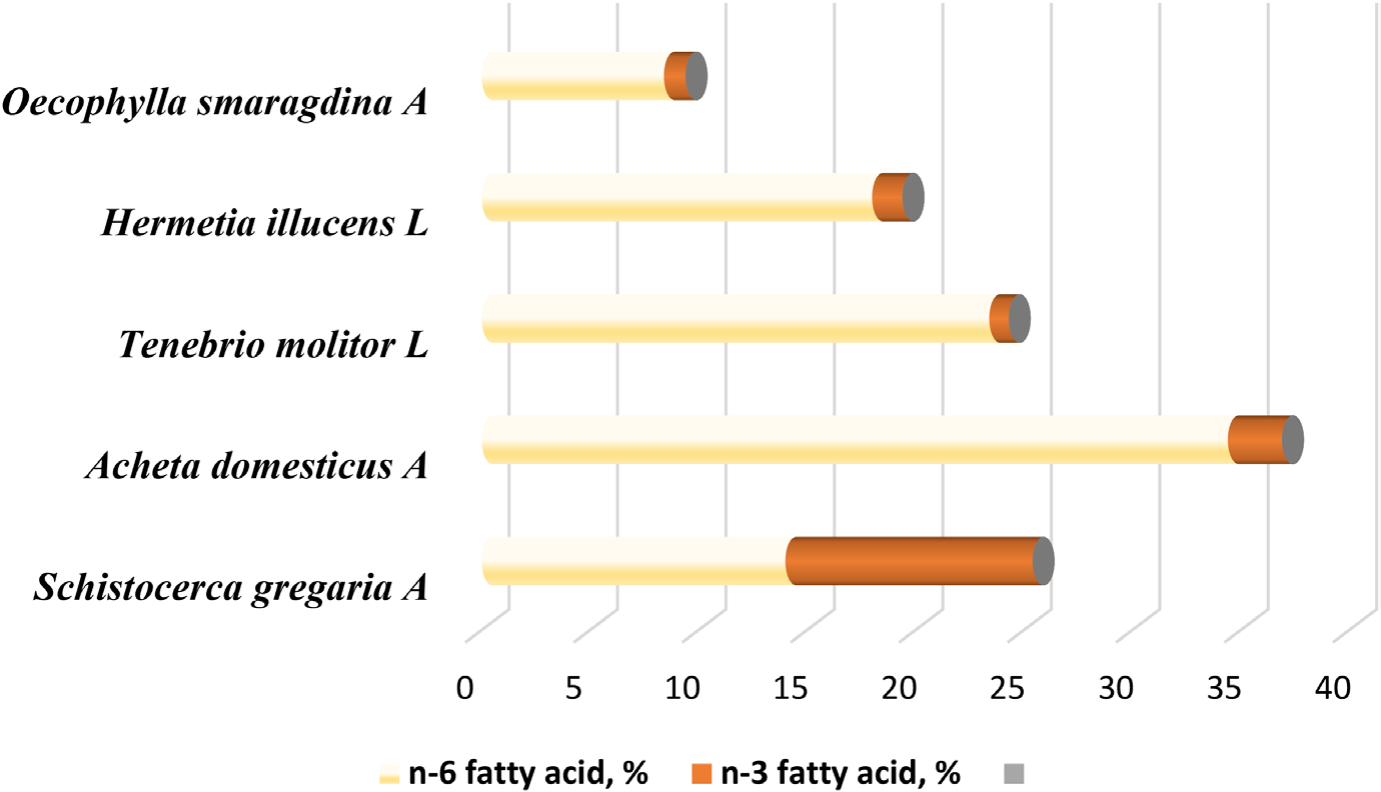
n6 and n3% amount in *Schistocerca gregaria*, *Acheta domesticus* , *Tenebrio molitor* , *Hermetia illucens* , and *Oecophylla smaragdina* (Data from Mishyna and Glumac 2021; values are based on dry weight, fresh weight, methods of lipid extraction).

Omega‐6/omega‐3 FAs ratio of whole insects varies from 1.2 in *S. gregaria* to 27.2 in *T. molitor* (Mishyna and Glumac 2021). The low omega‐6/omega‐3 FAs ratio of *S. gregaria* makes it more suitable for the reduction of the risk of a number of chronic diseases. Disproportionate amounts of omega‐6 PUFAs and a very high omega‐6/omega‐3 ratio cause many diseases such as cancer, cardiovascular disease, inflammatory and autoimmune diseases, while increasing the levels of omega‐3 PUFA, which means a lower omega‐6/omega‐3 ratio produces suppressive effects. Omega‐3 PUFAs also display protective effects against diabetes mellitus‐associated complications including diabetic retinopathy, diabetic nephropathy, and proteinuria (Lakshimi and Kavitha 2023). Omega amount in % was found balanced in *S. gregaria* (Figures 23 and 24). Insects' fat needs additional processing or mixing with other fat sources in order to achieve the wanted ratio of omega‐6/omega‐3. It should be mentioned that the oil extraction method particularly affects the yield and omega‐6/omega‐3 ratio (Tzompa‐Sosa et al. 2014) and can be controlled by the diet (Lehtovaara et al. 2017).

## 8 Consumer Acceptance of Insect Lipids

Though entomophagy is a usual practice in some African and Asian countries, it is still not accepted in others. In Western societies, the culture of eating animals as a source of protein might be a significant barrier toward edible insects (Clarkson et al. 2019; van Huis and Rumpold 2023). The actual investigation into consumer acceptance and factors affecting insect consumption highlighted different acceptance barriers and that the only sensibilization about the sustainability and nutritional benefits of insects is not enough (Clarkson et al. 2019). New strategies have to be adopted to overcome cultural barriers and initial emotions like disgust in order to encourage society to at least try insects ad food in the future. There is a great focus on studying insects mainly as a source of proteins while insect oil or fat, a valuable source of healthy compounds, might lead to an interesting sensorial experience.

Consumer acceptability of insect oil as frying oil was discussed by Tzompa‐Sosa et al. (2022) and Tzompa‐Sosa et al. (2023). Tzompa‐Sosa et al. (2022) studied the application of yellow mealworm oil (crude or deodorized) as a deep‐frying oil compared to commercial frying oil and their blends for potato chips to explore consumers' preferences and emotions. The authors found that potato chips fried in vegetable oil, deodorized oil, and the blend of vegetable and deodorized yellow mealworm oil were perceived as the tastiest, with natural flavor and the lowest aftertaste, lowest off‐flavor, and scored lowest for chemical flavor, contrary to the blend of vegetable oil plus crude yellow mealworm oil. Tzompa‐Sosa et al. (2022, 2023) concluded that deodorized yellow mealworm oil enhanced sensorial experiences and augmented consumer acceptance of dough and potato chips and were comparable to vegetable oil. The strongest acceptance and preference were found for the donuts fried in 100% deodorized yellow mealworm oil and in yellow mealworm oil blended with vegetable oil. Besides, consumer testing at home produced similar results in acceptance and preference.

An online cross‐cultural survey was conducted in France and China by Yan et al. (2024). In this study, Chinese had a higher willingness to eat insects than French participants, with a different preference ranking of 13 different insect‐based foods. In both countries, low insect visibility had a significant effect on consumers' willingness to eat insects. Consequently, the use of fat or lipids as invisible ingredients might increase consumer acceptance for insect‐based foods.

## 9 Challenges and Importance of Legal Framework

The insect industry is still dealing with some restrictions due to the out‐of‐date regulations that cover the use of insects for food and feed (Lahteenmaki‐Uutela et al. 2021; Lorrette and Sanchez 2022). Insects are sources of protein and lipid for food or feed. Eight insect species are authorized for the production of processed animal proteins intended as feed for farmed animals within Europe, according to the EU Regulation No 2017/893 (European Commission 2017): *G. sigillatus*, a tropical house cricket, *G. assimilis* , a Jamaican field cricket, *A. domesticus* , a house cricket, *T. molitor* , common in Europe as a pest of grain storages, *A. diaperinus* , also known as lesser mealworm, *H. illucens*, the BSF, *M. domestica*, a house fly, and *B. mori* , a silkworm. The substrates on which those insects may be reared are strictly defined in regulation and consist mainly of vegetal origin (Lahteenmaki‐Uutela et al. 2021). The regulation also applies to lipid fractions from the list of authorized species, since lipids are a side product during the processing of protein production from insects.

Fat and oil from insects have always been very reachable in the feed market differing to the implication of protein from insects still restricted by legislation (Lahteenmaki‐Uutela et al. 2021). Those products fall within the definition of animal fat as “product composed of fat from land animals, including invertebrates other than species pathogenic to humans and animals in all their life stages” (European Commission 2013 (EU) No 68/2013) therefore, they are allowed in aquaculture, poultry, pigs, pet food, and other animals' food. There is no need for any justification to EU authorities for the feed applications.

Entomophagy has no legal regulations in Africa (Kolobe et al. 2023; Grabowski et al. 2020). The use of fats from insects has been approved for a restricted number of edible insects including *A. diaperinus, A. domesticus*, and *L. migratoria*, which have been tested for safe consumption (Lähteenmäki ‐Uutela et al. 2018).

The ban was thereafter lifted since if consumed by humans or animals, edible insects pose less health risk. According to Żuk‐Gołaszewska et al. (2022), insect oils could be safely administered or supplemented in human and animal diets. Since food materials with high levels of SFAs such as palmitic acid and stearic acid are considered undesirable for consumption, the optimal inclusion levels of insects as a source of fats and FAs in diets must still be taken into consideration. Astrup et al. (2020) highlighted that different SFAs have also different biologic effects, which are additionally modified by the food matrix and the carbohydrate content of the food. Indeed, several foods relatively rich in SFAs, such as dark chocolate, whole‐fat dairy, and unprocessed meat, are not associated with increased cardiovascular diseases or diabetes risk. The general effect of fats and oils on health does not straightforwardly depend on the sum of the effects of the individual lipid components (Astrup et al. 2020). It depends, somewhat, on the interacting effects from naturally occurring components, overlooked in the assessment of health effects of oils and fats, and from unhealthy compounds introduced by food processing. Saturated fats were abundant and were always a key part of the ancient human diet (Astrup et al. 2020). Stearic acid (C18:0) from dark chocolate has a neutral effect on cardiovascular disease risk. Dark chocolate indeed has been reported to have multiple beneficial health effects such as antihypertensive, antiatherogenic, and potential antioxidative, anti‐inflammatory, and antithrombotic properties. Furthermore, dark chocolate has preventive effects against cardiovascular diseases and type 2 diabetes. Unprocessed red meat, for example, if consumed in modest amounts, is a major source of protein, minerals, bioavailable vitamins, and iron (Astrup et al. 2020). In many developing countries, meat constitutes a significant part of the diet for ancient populations and low‐income populations (Sandoval‐Insausti et al. 2016).

For human consumption instead, insects must be approved as a Novel Food under regulation (EU) No 2015/2283. Under this new regulation, insect food products may only be marketed when authorized after a safety assessment by the EFSA. Following its authorization as “novel food” by the Commission Implementing Regulation (EU) 2021/882, dried *T. molitor* larvae were placed legally on the EU market. This regulation defines the conditions and forms under which the insect product may be marketed, food categories in which it may be incorporated as an ingredient, labeling requirements, maximum thresholds for chemical and microbial contaminants, and applicable maximum limits. The safety of crickets and frozen mealworm was also positively assessed by EFSA. Numerous other applications for insect as proteins have been submitted for the agreement by EFSA. As a Novel Food product, insect oil and fat fall into the same requirements even though their composition is very comparable to the composition of some plant oils. It is mandatory to justify those products' safety, innocuity, and harmlessness for human consumption; therefore, the deposit of a novel food file is required. Such an authorization should be achieved soon for insect fat by virtue of its composition, oxidative stability, and safety aspects. The Novel Food Regulation aims to bring innovative food businesses to the EU and worldwide market, guaranteeing at the same time their safety and assuring a balance between innovation and safety. The novel food regulation works to guarantee the safety of novel foods for consumers. It ensures that they are properly labeled and not misleading. Therefore, Novel Foods can only be authorized in the absence of any risk to human health.

The company Ynsect NL B.V. applied for lesser mealworm; the company Cricket One Co. Ltd. applied for house cricket partially defatted powder. Both novel food applications went through stringent scientific assessments by EFSA, which concluded later that both of them are safe under the uses and use levels proposed by both companies (EU Health and Food Safety, n.d.).

Frozen, dried, and powder forms of *T. molitor* larva, frozen, dried, and powder forms of *L. migratoria*, and frozen, dried, and powder forms of *A. domesticus* are already authorized under the novel food regulation (EU Health and Food Safety, n.d.).

Uncertainty regarding the classification of whole insects under the Novel Food Regulation that explicitly considers whole insects as novel foods and if it must thus get an approval was clarified by the ruling of the European Court of Justice. On October 1, 2020, the court concluded that whole insects do not fall within the scope of that regulation and could therefore be placed on the market without a premarket authorization (EU Health and Food Safety, n.d.). The importing countries of insects intended for human consumption are regulated by Article 20 of Regulation (EU) 2019/626 that was next amended on February 15, 2021 with the implementing regulation (EU) 2021/17. According to this regulation, in addition to Canada, Thailand, Vietnam, South Korea, and Switzerland are also included as importing countries of insects intended for human consumption.

On May 3, 2021, larvae of the yellow mealworm *T. molitor* from the black beetle family made the start and were announced as authorized as a novel food.

## 10 Conclusion

Due to the global growing interest in integrating insect‐derived ingredients into the circular economy, extensive studies have been conducted revealing the excellent nutritional qualities of edible insectsas a source of high protein and FAs content.

Actually, though there are still many restrictions on the use of insect products in food and feed. The lifting of those restrictions would play a key role in sponsoring insect farming as a source of essential fats and FAs for humans and animals. Fats and FAs are found in abundance in most edible insects, and they may meet the recommended fats and FAs levels indispensable for humans. If incorporated in human diets, they could play an important role as a source of energy and by providing essential PUFAs required for the normal functioning of human body cells. Insects as a source of fats and FAs in human diets are still to be investigated since any food with high levels of SFAs such as palmitic acid and stearic acid is reported as undesirable for consumption. The lipid profile of insects could be manipulated through various processing techniques, which contribute to improving their nutrient content.

Legislation must establish regulations to guarantee the safety of food and feed from insects.

Large‐scale insect farming could minimize food safety issues other than preventing contamination during the farming process. Processing techniques are essential to produce safe, high‐quality, and nutritious edible insect products. Research should focus on recognizing potential allergens and toxins and contribute to developing effective control measures.

This review aimed to contribute to the essential social awareness and promote the culture and utilization of edible insect resources in the circular economy.

Insects play a critical role in food and agricultural industries for both the local economy and the global community. Scientific research can improve the culture of utilization of edible insect resources.

The long‐standing bias against foods that are rich in saturated fats has to be reconsidered with a view toward recommending diets consisting of healthy foods by enhancing the people's understanding that many foods which may be rich in saturated fats could also play an important role in meeting dietary and nutritional recommendations. Low‐carbohydrate diets high in saturated fat may improve metabolic disease endpoints in some individuals, but emphasize that the health effects of dietary carbohydrate, just like those of saturated fat, depend on the amount, type, and quality of carbohydrate, food sources, and degree of processing.

The dietary recommendation about reducing the intake of SFAs has to take into consideration specific FAs and food sources. Future research on fat or oil from insects has to consider the types of FAs, the different insects' composition of SFAs, that may possess harmful, neutral, or even beneficial effects in relation to major health outcomes. Research has to focus on the effect of consuming unprocessed or gently processed insects that are more likely a key factor. Further informations about the health effects of specific process contaminants should be examined so that their levels can be minimized.

There is a critical need for searching alternate lipid sources for feed, food, biodiesel, cosmetic, and pharmaceutical industries. Solid Safety Regulation would bring innovative food businesses to the EU and worldwide market, guaranteeing at the same time their safety. As a consequence, the knowledge about health benefits of insect oils as an alternative food source could help to improve the global market of insect and farming practices in communities with limited access to food sources. To conclude, based on a good FA profile with PUFAs, edible insects make a valuable alternative for feed and food as long as it is safe, legal, and accepted. For this reason, more research is required to evaluate their optimal inclusion level and their safety as a source of fats and FAs in diets.

Edible insects' lipids could address many serious issues such as malnutrition and food security issues. Still, they require low soil and water resources. They are able to reduce waste with less greenhouse gas emissions, which gives them a lipid source ecologically significant.

## Author Contributions

**Shahida Anusha Siddiqui:** conceptualization (equal), formal analysis (equal), methodology (equal), supervision (equal), validation (equal), writing – review and editing (equal). **Asma Zeiri:** conceptualization (equal), data curation (equal), formal analysis (equal), methodology (equal), validation (equal), writing – original draft (equal), writing – review and editing (equal). **Mohd Asif Shah:** supervision (equal).

## Ethics Statement

The authors have nothing to report.

## Consent

The authors have nothing to report.

## Conflicts of Interest

The authors declare no conflicts of interest.

## Data Availability

The authors have nothing to report.

## References

[fsn370553-bib-0001] Akullo, J. , J. G. Agea , B. B. Obaa , J. Okwee‐Acai , and D. Nakimbugwe . 2018. “Nutrient Composition of Commonly Consumed Edible Insects in the Lango Sub‐Region of Northern Uganda.” International Food Research Journal 25, no. 1: 159–165.

[fsn370553-bib-0003] Al‐Esawy, M. T. 2023. “Honey Bee Immunity and Physiology Are Enhanced by Consuming High‐Fat Diets.” Journal of Plant Protection Research 63, no. 2: 185–195. 10.24425/jppr.2023.145753.

[fsn370553-bib-0002] Alengebawy, A. , S. T. Abdelkhalek , S. R. Qureshi , and M. Q. Wang . 2021. “Heavy Metals and Pesticides Toxicity in Agricultural Soil and Plants: Ecological Risks and Human Health Implications.” Toxics 9, no. 3: 42. 10.3390/toxics.90300-42. 33668829 PMC7996329

[fsn370553-bib-0004] Alnajim, I. , X. Du , B. Lee , M. Agarwal , T. Liu , and Y. Ren . 2019. “New Method of Analysis of Lipids in *Tribolium castaneum* (Herbst) and *Rhyzopertha dominica* (Fabricius) Insects by Direct Immersion Solid‐Phase Microextraction (DI‐SPME) Coupled With GC‐MS.” Insects 10, no. 10: 363. 10.3390/insects10100363. 31635132 PMC6835878

[fsn370553-bib-0270] Aluyor, E. O. , K. O. Obahiagbon , and M. Ori‐Jesu . 2009. “Biodegradation of Vegetable Oils: A Review.” Scientific Research and Essay 4, no. 6: 543–548.

[fsn370553-bib-0006] Alves, A. V. , E. J. Sanjinez Argandoña , A. M. Linzmeier , C. A. L. Cardoso , and M. L. R. Macedo . 2016. “Chemical Composition and Food Potential of *Pachymerus nucleorum* Larvae Parasitizing *Acrocomia aculeata* Kernels.” PLoS One 11, no. 3: e0152125. 10.1371/journal.pone.0152125. 27031500 PMC4816322

[fsn370553-bib-0007] Alves, A. V. , E. J. Sanjinez‐Argandoña , A. M. Linzmeier , C. A. L. Cardoso , and M. L. R. Macedo . 2016. “Food Value of Mealworm Grown on *Acrocomia aculeata* Pulp Flour.” PLoS One 11: e0151275. 26974840 10.1371/journal.pone.0151275 PMC4807927

[fsn370553-bib-0005] Alves, A. V. , F. Freitas de Lima , T. Granzotti da Silva , V. S. Oliveira , C. A. L. Kassuya , and E. J. Sanjinez‐Argandoña . 2019. “Safety Evaluation of the Oils Extracted From Edible Insects (*Tenebrio molitor* and *Pachymerus nucleorum*) as Novel Food for Humans.” Regulatory Toxicology and Pharmacology 102: 90–94. 10.1016/j.yrtph.2019.01.013. 30611818

[fsn370553-bib-0259] Anankware, J. P. , B. J. Roberts , X. Cheseto , I. Osuga , V. Savolainen , and C. M. Collins . 2021. “The Nutritional Profiles of Five Important Edible Insect Species From West Africa‐An Analytical and Literature Synthesis.” Frontiers in Nutrition 8: 792941. 10.3389/fnut.2021.792941. 34926558 PMC8678595

[fsn370553-bib-0008] Anankware, P. J. , K. O. Fening , E. Osekre , and D. Obeng‐Ofori . 2015. “Insects as Food and Feed: A Review.” International Journal of Agricultural Research and Review 3, no. 1: 143–151.

[fsn370553-bib-0290] André, D. , R. Thiago , B. Justin , L. Elizabeth , and W. Erika . 2018. “The Oil Fraction and Partially Defatted Meal of Black Soldier Fly Larvae (*Hermetia illucens*) Affect Differently Growth Performance, Feed Efficiency, Nutrient Deposition, Blood Glucose and Lipid Digestibility of Rainbow Trout (*Oncorhynchus mykiss*).” Aquaculture 492: 24–34. 10.1016/j.aquaculture.2018.03.038.

[fsn370553-bib-0248] Araújo, R. R. , T. A. dos Santos Benfica , V. P. Ferraz , and E. M. Santos . 2019. “Nutritional Composition of Insects *Gryllus assimilis* and *Zophobas morio*: Potential Foods Harvested in Brazil.” Journal of Food Composition and Analysis 76: 22–26. 10.1016/j.jfca.2018.11.005.

[fsn370553-bib-0009] Arrese, E. L. , and J. L. Soulages . 2010. “Insect Fat Body: Energy, Metabolism, and Regulation.” Annual Review of Entomology 55: 207–225. 10.1146/annurev-ento-112408-085356 PMC3075550 19725772

[fsn370553-bib-0010] Astrup, A. , F. Magkos , D. M. Bier , et al. 2020. “Saturated Fats and Health: A Reassessment and Proposal for Food‐Based Recommendations: JACC State‐Of‐The‐Art Review.” Journal of the American College of Cardiology 76, no. 7: 844–857. 10.1016/j.jacc.2020.05.077. 32562735

[fsn370553-bib-0011] Baiano, A. 2020. “Edible Insects: An Overview on Nutritional Characteristics, Safety, Farming, Production Technologies, Regulatory Framework, and Socio‐Economic and Ethical Implications.” Trends in Food Science and Technology 100: 35–50. 10.1016/j.tifs.2020.03.040.

[fsn370553-bib-0012] Barennes, H. , M. Phimmasane , and C. Rajaonarivo . 2015. “Insect Consumption to Address Undernutrition, a National Survey on the Prevalence of Insect Consumption Among Adults and Vendors in Laos.” PLoS One 10, no. 8: e0136458. 10.1371/journal.pone.0136458. 26317772 PMC4552653

[fsn370553-bib-0013] Barre, A. , M. Simplicien , G. Cassan , H. Benoist , and P. Rougé . 2018. “Food Allergen Families Common to Different Arthropods (Mites, Insects, Crustaceans), Mollusks and Nematods: Cross‐Reactivity and Potential Cross‐Allergenicity.” Revue Française d'Allergologie 58, no. 8: 581–593. 10.1016/j.reval.2018.10.008.

[fsn370553-bib-0014] Batalha, M. M. C. , H. F. Goulart , A. E. G. Santana , et al. 2020. “Chemical Composition and Antimicrobial Activity of Cuticular and Internal Lipids of the Insect *Rhynchophorus palmarum* .” Archives of Insect Biochemistry and Physiology 105: e21723. 10.1002/arch.21723. 32623787

[fsn370553-bib-0015] Bednarska, A. J. , M. Opyd , E. Żurawicz , and R. Laskowski . 2015. “Regulation of Body Metal Concentrations: Toxicokinetics of Cadmium and Zinc in Crickets.” Ecotoxicology and Environmental Safety 119: 9–14. 10.1016/j.ecoenv.2015.04.056. 25958030

[fsn370553-bib-0289] Belghit, I. , N. S. Liland , R. Waagbø , et al. 2018. “Potential of Insect‐Based Diets for Atlantic Salmon (*Salmo salar*).” Aquaculture 491: 72–81.

[fsn370553-bib-0017] Benzertiha, A. , B. Kierończyk , M. Rawski , et al. 2020. “Insect Fat in Animal Nutrition – A Review.” Annals of Animal Science 20, no. 4: 1217–1240. 10.2478/aoas-2020-0076.

[fsn370553-bib-0016] Benzertiha, A. , B. Kieronczyk , M. Rawski , P. Kołodziejski , M. Bryszak , and D. Józefiak . 2019. “Insect Oil as an Alternative to Palm Oil and Poultry Fat in Broiler Chicken Nutrition.” Animals 9: 116–133. 30934626 10.3390/ani9030116 PMC6465997

[fsn370553-bib-0018] Berezina, N. 2017. “Insects: Novel Source of Lipids for a Fan of Applications.” OCL 24, no. 4: D402.

[fsn370553-bib-0019] Biancarosa, I. , N. S. Liland , D. Biemans , et al. 2018. “Uptake of Heavy Metals and Arsenic in Black Soldier Fly ( *Hermetia illucens* ) Larvae Grown on Seaweed‐Enriched Media.” Journal of the Science of Food and Agriculture 98, no. 6: 2176–2183. 10.1002/jsfa.8702. 28960324

[fsn370553-bib-0020] Borrelli, L. , L. Varriale , L. Dipineto , A. Pace , L. F. Menna , and A. Fioretti . 2021. “Insect Derived Lauric Acid as Promising Alternative Strategy to Antibiotics in the Antimicrobial Resistance Scenario.” Frontiers in Microbiology 12: 620798. 33717009 10.3389/fmicb.2021.620798 PMC7952302

[fsn370553-bib-0021] Bovera, F. , R. Loponte , M. E. Pero , et al. 2018. “Laying Performance, Blood Profiles, Nutrient Digestibility and Inner Organs Traits of Hens Fed an Insect Meal From *Hermetia illucens* Larvae.” Research in Veterinary Science 120: 86–93. 30293041 10.1016/j.rvsc.2018.09.006

[fsn370553-bib-0262] Bukkens, S. G. 2005. “Insects in the Human Diet: Nutritional Aspects.” In Ecological Implications of Minilivestock: Potential of Insects, Rodents, Frogs and Snails, edited by M. G. Paoletti , 545–577. Science Publishers, Inc.

[fsn370553-bib-0022] Canavoso, L. E. , Z. E. Jouni , K. J. Karnas , J. E. Pennington , and M. A. Wells . 2001. “Fat Metabolism in Insects.” Annual Review of Nutrition 21: 23–46. 10.1146/annurev.nutr.21.1.23. 11375428

[fsn370553-bib-0292] Chen, H. , J. Tian , Y. Wang , K. Yang , H. Ji , and J. Li . 2017. “Effects of Dietary Soybean Oil Replacement by Silkworm, *Bombyx mori* L., Chrysalis Oil on Growth Performance, Tissue Fatty Acid Composition, and Health Status of Juvenile Jian Carp, *Cyprinus carpio* var. Jian.” Journal of the World Aquaculture Society 48: 453–466. 10.1111/jwas.12373.

[fsn370553-bib-0023] Cheseto, X. , S. B. S. Baleba , C. M. Tanga , S. Kelemu , and B. Torto . 2020. “Chemistry and Sensory Characterization of a Bakery Product Prepared With Oils From African Edible Insects.” Food 9: 800. 10.3390/foods9060800. PMC7353482 32570724

[fsn370553-bib-0024] Chng, W.‐b. A. , V. Hietakangas , and B. Lemaitre . 2017. “Physiological Adaptations to Sugar Intake: New Paradigms From *Drosophila melanogaster* .” Trends in Endocrinology and Metabolism 28, no. 2: 131–142. 10.1016/j.tem.2016.11.003. 27923532

[fsn370553-bib-0025] Choe, D. H. , S. R. Ramírez , and N. D. Tsutsui . 2012. “A Silica Gel Based Method for Extracting Insect Surface Hydrocarbons.” Journal of Chemical Ecology 38, no. 2: 176–187. 10.1007/s10886-012-0074-1. 22327277

[fsn370553-bib-0026] Chomchai, S. , and C. Chomchai . 2018. “Histamine Poisoning From Insect Consumption: An Outbreak Investigation From Thailand.” Clinical Toxicology 56, no. 2: 126–131. 10.1080/15563650.2017.1349320. 28748745

[fsn370553-bib-0258] Cito, A. , E. Dreassi , R. Frosinini , et al. 2017. “The Potential Beneficial Effects of *Tenebrio molitor* (Coleoptera Tenebrionidae) and *Galleria mellonella* (Lepidoptera Pyralidae) on Human Health.” Redia 100: 125–133. 10.19263/REDIA-100.17.16.

[fsn370553-bib-0027] Clarkson, C. , B. John , and M. Miranda . 2019. “Locusts as a Source of Lipids and Proteins and Consumer Acceptance.” In Encyclopedia of Food Chemistry, edited by L. Melton , F. Shahidi , and P. Varelis , 167–172. Academic Press. 10.1016/B978-0-08-100596-5.22420-7.

[fsn370553-bib-0245] Commission Regulation . 2013. “Commission Regulation (EU).” No 68/2013 of 16 January 2013 on the Catalogue of feed materials,EU, Brussels, belgium.

[fsn370553-bib-0028] Costa‐Neto, E. M. , and F. V. Dunkel . 2016. “Chapter 2 ‐ Insects as Food: History, Culture, and Modern Use Around the World.” In Insects as Sustainable Food Ingredients, edited by A. T. Dossey , J. A. Morales‐Ramos , and M. G. Rojas , 29–60. Academic Press.

[fsn370553-bib-0029] Cullere, M. , A. Schiavone , S. Dabbou , L. Gasco , and A. D. Zotte . 2019. “Meat Quality and Sensory Traits of Finisher Broiler Chickens Fed With Black Soldier Fly (*Hermetia illucens* L.) Larvae Fat as Alternative Fat Source.” Animals 9: 140. 30986996 10.3390/ani9040140 PMC6523764

[fsn370553-bib-0030] Cutrignelli, M. I. , M. Messina , F. Tulli , et al. 2018. “Evaluation of an Insect Meal of the Black Soldier Fly (*Hermetia illucens*) as Soybean Substitute: Intestinal Morphometry, Enzymatic and Microbial Activity in Laying Hens.” Research in Veterinary Science 117: 209–215. 29304440 10.1016/j.rvsc.2017.12.020

[fsn370553-bib-0031] Dabbou, S. , I. Ferrocino , and L. Gasco . 2020. “Antimicrobial Effects of Black Soldier Fly and Yellow Mealworm Fats and Their Impact on Gut Microbiota of Growing Rabbits.” Animals 10: 1292–1310. 10.3390/ani10081292. 32731566 PMC7460256

[fsn370553-bib-0296] Dayrit, F. M. 2014. “Lauric Acid is a Medium‐Chain Fatty Acid, Coconut Oil is a Medium‐Chain Triglyceride.” Philippine Journal of Science 143: 157–166.

[fsn370553-bib-0032] Debbarma, S. , S. Deb , N. K. Yadav , et al. 2025. “Waste Not, Want Not: Unlocking the Innovative Potential of Organic and Eco‐Friendly Insect and Algal Resources for Future Aquaculture.” Aquaculture International 33: 158. 10.1007/s10499-024-01814-8.

[fsn370553-bib-0033] Delicato, C. , J. J. Schouteten , K. Dewettinck , X. Gellynck , and D. A. Tzompa‐Sosa . 2020. “Consumers' Perception of Bakery Products With Insect Fat as Partial Butter Re‐Placement.” Food Quality and Preference 79: 103755.

[fsn370553-bib-0034] Devi, W. D. , R. Bonysana , K. Kapesa , P. K. Mukherjee , and Y. Rajashekar . 2022. “Ethnotherapeutic Practice of Entomophagy Species by the Ethnic Community of Tangkhul, Mao and Poumai Community of Manipur, NER India.” Journal of Ethnic Foods 9: 17. 10.1186/s42779-022-00132-9.

[fsn370553-bib-0035] Diener, S. , C. Zurbrügg , and K. Tockner . 2015. “Bioaccumulation of Heavy Metals in the Black Soldier Fly, Hermetia Illucens and Effects on Its Life Cycle.” Journal of Insects as Food and Feed 1, no. 4: 261–270. 10.3920/JIFF2015.0030.

[fsn370553-bib-0273] Dijkstra, A. J. 2016. “Vegetable Oils: Composition and Analysis.” In Encyclopedia of Food and Health, edited by B. Caballero , P. M. Finglas , and F. Toldrá , 357–364. Academic Press. 10.1016/B978-0-12-384947-2.00708-X.

[fsn370553-bib-0036] Dobermann, D. , J. A. Swift , and L. M. Field . 2017. “Opportunities and Hurdles of Edible Insects for Food and Feed.” Nutrition Bulletin 42, no. 4: 293–308. 10.1111/nbu.12291.

[fsn370553-bib-0251] Doley, A. K. , and J. Kalita . 2012. “Traditional Uses of Insect and Insect Products in Medicine and Food by the Mishing Tribe of Dhemaji District, Assam, North‐East India.” Social Science Research 1, no. 2: 11–21.

[fsn370553-bib-0037] dos Santos, A. G. 2021. “An Overview of Lipids From Insects.” Biocatalysis and Agricultural Biotechnology 33: 101967. 10.1016/j.bcab.2021.101967.

[fsn370553-bib-0038] dos Santos, O. V. , P. C. S. Dias , S. D. Soares , L. R. V. da Conceição , and B. E. Teixeira‐Costa . 2021. “Artisanal Oil Obtained From Insects' Larvae (*Speciomerus ruficornis*): Fatty Acids Composition, Physicochemical, Nutritional and Antioxidant Properties for Application in Food.” European Food Research and Technology 247: 1803–1813. 10.1007/s00217-021-03752-8.

[fsn370553-bib-0039] Downs, M. , P. Johnson , and M. Zeece . 2016. “Chapter 9 ‐ Insects and Their Connection to Food Allergy.” In Insects as Sustainable Food Ingredients: Production Processing and Food Application, edited by A. T. Dossey , J. A. Morales‐Ramos , and M. G. Rojas , 255–272. Nikki Levy. 10.1016/B978-0-12-802856-8.00009-0.

[fsn370553-bib-0040] Dreassi, E. , A. Cito , A. Zanfini , L. Materozzi , M. Botta , and V. Francardi . 2017. “Dietary Fatty Acids Influence the Growth and Fatty Acid Composition of the Yellow Mealworm *Tenebrio molitor* (Coleoptera: Tenebrionidae).” Lipids 52, no. 3: 285–294. 10.1007/s11745-016-4220-3. 28083781

[fsn370553-bib-0042] EFSA Panel on Dietetic Products, Nutrition and Allergies (NDA) . 2016. “Guidance on the Preparation and Presentation of an Application for Authorisation of a Novel Food in the Context of Regulation (EU) 2015/2283.” EFS2 2016, 14. 10.2903/j.efsa.2021.6555 PMC7996107 33791039

[fsn370553-bib-0041] EFSA . 2015. “Scientific Committee. Risk Profile Related to Production and Consumption of Insects as Food and Feed.” EFSA Journal 13, no. 10: 4257. 10.2903/j.efsa.2015.4257.

[fsn370553-bib-0044] Elahi, U. , C. Xu , J. Wang , et al. 2022. “Insect Meal as a Feed Ingredient for Poultry.” Animal Bioscience 35, no. 2: 332–346. 10.5713/ab.21.0435. 34991217 PMC8831830

[fsn370553-bib-0045] Energy UD of 2016 . 2016. “International Energy Outlook 2016.”

[fsn370553-bib-0046] Enriquez, T. , and B. Visser . 2023. “The Importance of Fat Accumulation and Reserves for Insect Overwintering.” Current Opinion in Insect Science 60: 101118. 10.2139/ssrn.4389731. 37739063

[fsn370553-bib-0047] EU Health and Food Safety . n.d. “Novel Food Regulation.” Accessed March 15, 2024. https://food.ec.europa.eu/safety/novel‐food/authorisations/approval‐insect‐novel‐food_en.

[fsn370553-bib-0050] European Commission . 2017. “Commission Regulation (EU) 2017/997 of 13 May 2017 Amending Annexes I and IV to Regulation (EC) No 99/2009 as Regards the Placing on the Market and the Use of Feed From Insects.” Official Journal of the European Union, L 136. 1–6.

[fsn370553-bib-0053] FAO . 2021. Looking at Edible Insects from a Food Safety Perspective. Challenges and Opportunities for the Sector. FAO. 10.4060/cb4094.en.

[fsn370553-bib-0055] Fels‐Klerx, V. D. H. J. , L. Camenzuli , M. K. van der Lee , and D. G. Oonincx . 2016. “Uptake of Cadmium, Lead and Arsenic by *Tenebrio molitor* and *Hermetia illucens* From Contaminated Substrates.” PLoS One 11, no. 11: e0166186. 10.1371/journal.pone.0166186. 27846238 PMC5112862

[fsn370553-bib-0054] Fels‐Klerx, V. D. H. J. , L. Camenzuli , S. Belluco , N. Meijer , and A. Ricci . 2018. “Food Safety Issues Related to Uses of Insects for Feeds and Foods.” Comprehensive Reviews in Food Science and Food Safety 17, no. 5: 1172–1183. 10.1111/1541-4337.12385. 33350154

[fsn370553-bib-0057] Feng, Y. , M. Zhao , W. F. Ding , and X. M. Chen . 2020. “Overview of Edible Insect Resources and Common Species Utilisation in China.” Journal of Insects as Food and Feed 6, no. 1: 13–25.

[fsn370553-bib-0056] Feng, Y. , X. Chen , M. Zhao , et al. 2018. “Edible Insects in China: Utilization and Prospects.” Insect Science 25, no. 2: 184–198. 10.1111/1744-7917.12449. 28225201

[fsn370553-bib-0058] Ferdousi, L. , M. Begum , M. S. Yeasmin , et al. 2023. “Facile Acid Fermentation Extraction of Silkworm Pupae Oil and Evaluation of Its Physical and Chemical Properties for Utilization as Edible Oil.” Heliyon 9: e12815. 36647348 10.1016/j.heliyon.2023.e12815 PMC9840356

[fsn370553-bib-0059] Ferreira‐Caliman, M. J. , I. C. Turatti , N. P. Lopes , R. Zucchi , and F. S. Nascimento . 2012. “Analysis of Insect Cuticular Compounds by Non‐Lethal Solid Phase Micro Extraction With Styrene‐Divinylbenzene Copolymers.” Journal of Chemical Ecology 38, no. 4: 418–426. 10.1007/s10886-012-0109-7. 22476959

[fsn370553-bib-0265] Fombong, F. , J. N. Kinyuru , J. Ng'ang'a , et al. 2021. “Affordable Processing of Edible Orthopterans Provides a Highly Nutritive Source of Food Ingredients.” Food 10, no. 1: 144. 10.3390/foods10010144. PMC7826988 33445608

[fsn370553-bib-0252] Fontaneto, D. , M. Tommaseo‐Ponzetta , C. Galli , P. Risé , R. H. Glew , and M. G. Paoletti . 2011. “Differences in Fatty Acid Composition between Aquatic and Terrestrial Insects Used as Food in Human Nutrition.” Ecology of Food and Nutrition 50, no. 4: 351–367. 10.1080/03670244.2011.586316. 21888601

[fsn370553-bib-0061] Franco, A. , C. Scieuzo , R. Salvia , et al. 2021. “Lipids From *Hermetia illucens*, an Innovative and Sustainable Source.” Sustainability 13, no. 18: 10198. 10.3390/su131810198.

[fsn370553-bib-0060] Franco, A. , R. Salvia , C. Scieuzo , E. Schmitt , A. Russo , and P. Falabella . 2022. “Lipids From Insects in Cosmetics and for Personal Care Products.” Insects 13: 41. 10.3390/insects13010041. PMC8779901 35055884

[fsn370553-bib-0062] Freel, T. A. , A. McComb , and E. A. Koutsos . 2021. “Digestibility and Safety of Dry Black Soldier Fly Larvae Meal and Black Soldier Fly Larvae Oil in Dogs.” Journal of Animal Science 99, no. 3: skab047. 10.1093/jas/skab047. 33585915 PMC7999617

[fsn370553-bib-0063] Fruttero, L. L. , J. Leyria , and L. E. Canavoso . 2017. “Lipids in Insect Oocytes: From the Storage Pathways to Their Multiple Functions.” Results and Problems in Cell Differentiation 63: 403–434. 10.1007/978-3-319-60855-6_18. 28779328

[fsn370553-bib-0065] Fukumura, K. , T. Konuma , Y. Tsukamoto , and S. Nagata . 2018. “Adipokinetic Hormone Signaling Determines Dietary Fatty Acid Preference Through Maintenance of Hemolymph Fatty Acid Composition in the Cricket *Gryllus bimaculatus* .” Scientific Reports 8, no. 1: 4737. 10.1038/s41598-018-22987-2. 29549314 PMC5856772

[fsn370553-bib-0066] Gáliková, M. , M. Diesner , P. Klepsatel , et al. 2015. “Energy Homeostasis Control in Drosophila Adipokinetic Hormone Mutants.” Genetics 201: 665–683. 10.1534/genetics.115.178897. 26275422 PMC4596676

[fsn370553-bib-0300] Gao, Y. , D. Wang , M. L. Xu , S. S. Shi , and J. F. Xiong . 2018. “Toxicological Characteristics of Edible Insects in China: A Historical Review.” Food and Chemical Toxicology 119: 237–251. 10.1016/j.fct.2018.04.016. 29649491

[fsn370553-bib-0067] Ghosh, A. , M. Ray , and D. Gangopadhyay . 2020. “Evaluation of Proximate Composition and Antioxidant Properties in Silk‐Industrial Byproduct.” LWT ‐ Food Science and Technology 132: 109900.

[fsn370553-bib-0068] Ghosh, S. , S.‐M. Leeb , C. Jungb , and V. B. Meyer‐Rochowc . 2017. “Nutritional Composition of Five Commercial Edible Insects in South Korea.” Journal of Asia‐Pacific Entomology 20: 686–694.

[fsn370553-bib-0069] Giannetto, A. , S. Oliva , C. F. Ceccon Lanes , et al. 2020. “ *Hermetia illucens* (Diptera: Stratiomydae) Larvae and Prepupae: Biomass Production, Fatty Acid Profile and Expression of Key Genes Involved in Lipid Metabolism.” Journal of Biotechnology 307: 44–54. 31678205 10.1016/j.jbiotec.2019.10.015

[fsn370553-bib-0070] Gil, A. , W. Zhang , J. C. Wolters , et al. 2018. “One‐ Vs Two‐Phase Extraction: Re‐Evaluation of Sample Preparation Procedures for Untargeted Lipidomics in Plasma Samples.” Analytical and Bioanalytical Chemistry 410: 5859–5870. 29968103 10.1007/s00216-018-1200-x PMC6096717

[fsn370553-bib-0075] Gołębiowski, M. , E. Maliński , J. Nawrot , and P. Stepnowski . 2008. “Identification and Characterization of Surface Lipid Components of the Dried‐Bean Beetle *Acanthoscelides obtectus* (Say) (Coleoptera: Bruchidae).” Journal of Stored Products Research 44: 386–388. 10.1016/j.jspr.2008.02.010.

[fsn370553-bib-0074] Gołebiowski, M. , E. Maliński , M. I. Boguś , J. Kumirska , and P. Stepnowski . 2008. “The Cuticular Fatty Acids of *Calliphora vicina* , Dendrolimus Pini and Galleria Mellonella Larvae and Their Role in Resistance to Fungal Infection.” Insect Biochemistry and Molecular Biology 38, no. 6: 619–627. 10.1016/j.ibmb.2008.03.005. 18510973

[fsn370553-bib-0072] Gołębiowski, M. , M. Cerkowniak , A. Urbanek , et al. 2014. “Antimicrobial Activity of Untypical Lipid Compounds in the Cuticular and Internal Lipids of Four Fly Species.” Journal of Applied Microbiology 116: 269–287. 10.1111/jam.12370. 24238211

[fsn370553-bib-0073] Gołębiowski, M. , M. Cerkowniak , A. Urbanek , M. Słocińska , G. Rosiński , and P. Stepnowski . 2014. “Adipokinetic Hormone Induces Changes in the Fat Body Lipid Composition of the Beetle *Zophobas atratus* .” Peptides 58: 65–73. 24905623 10.1016/j.peptides.2014.05.013

[fsn370553-bib-0071] Gołębiowski, M. , M. I. Boguś , M. Paszkiewicz , and P. Stepnowski . 2011. “Cuticular Lipids of Insects as Potential Biofungicides: Methods of Lipid Composition Analysis.” Analytical and Bioanalytical Chemistry 399: 3177–3191. 10.1007/s00216-010-4439-4. 21153591

[fsn370553-bib-0076] Gondim, K. C. , G. C. Atella , E. G. Pontes , and D. Majerowicz . 2018. “Lipid Metabolism in Insect Disease Vectors.” Insect Biochemistry and Molecular Biology 101: 108–123. 10.1016/j.ibmb.2018.08.005. 30171905

[fsn370553-bib-0308] Grabowski, N. T. , S. Tchibozo , A. Abdulmawjood , et al. 2020. “Edible Insects in Africa in Terms of Food, Wildlife Resource, and Pest Management Legislation.” Food 9: 502. 10.3390/foods9040502. PMC7230556 32316132

[fsn370553-bib-0077] Gutiérrez, Y. , M. Fresch , C. Scherber , and J. Brockmeyer . 2022. “The Lipidome of an Omnivorous Insect Responds to Diet Composition and Social Environment.” Ecology and Evolution 12, no. 11: e9497. 10.1002/ece3.9497. 36381391 PMC9643132

[fsn370553-bib-0078] Hammoud Mahdi, D. , J. Hubert , J.‐H. Renault , et al. 2020. “Chemical Profile and Antimicrobial Activity of the Fungus‐Growing Termite Strain *Macrotermes Bellicosus* Used in Traditional Medicine in the Republic of Benin.” Molecules 25: 5015. 10.3390/molecules25215015. 33138110 PMC7662623

[fsn370553-bib-0079] Han, S. H. , and C. Bordereau . 1982. “Ultrastrucuture of the Fat Body of the Reproductive Pair in Higher Termites.” Journal of Morphology 172, no. 3: 313–322. 10.1002/jmor.1051720306. 30110994

[fsn370553-bib-0281] He, S. , X. Liu , W. Xu , S. Qi , W. Lian , and W. Wang . 2022. “Transesterification Synthesis of High‐Yield Biodiesel From Black Soldier Fly Larvae by Using the Combination of Lipase Eversa Transform 2.0 and Lipase SMG1.” Food Science and Technology (online) 42, no. 6: e103221.

[fsn370553-bib-0080] Hender, A. , M. A. B. Siddik , J. Howieson , and R. Fotedar . 2021. “Black Soldier Fly, *Hermetia illucens* as an Alternative to Fishmeal Protein and Fish Oil: Impact on Growth, Immune Response, Mucosal Barrier Status, and Flesh Quality of Juvenile Barramundi, *Lates calcarifer* .” Biology 10: 505–521. 34200162 10.3390/biology10060505 PMC8230191

[fsn370553-bib-0082] Henry, M. A. , L. Gasco , G. Piccolo , and E. Fountoulaki . 2015. “Review on the Use of Insects in the Diet of Farmed Fish: Past and Future.” Animal Feed Science and Technology 203: 1–22.

[fsn370553-bib-0083] Hlongwane, Z. T. , R. Slotow , and T. C. Munyai . 2020. “Indigenous Knowledge About Consumption of Edible Insects in South Africa.” Insects 12, no. 1: 22. 10.3390/insects12010022. 33396313 PMC7824724

[fsn370553-bib-0085] Hoekman, S. K. , A. Broch , C. Robbins , E. Ceniceros , and M. Natarajan . 2012. “Review of Biodiesel Composition, Properties, and Specifications.” Renewable and Sustainable Energy Reviews 16, no. 1: 143–169.

[fsn370553-bib-0086] Hossain, S. , B. C. Small , and R. Hardy . 2023. “Insect Lipid in Fish Nutrition: Recent Knowledge and Future Application in Aquaculture.” Reviews in Aquaculture 15: 1664–1685. 10.1111/raq.12810.

[fsn370553-bib-0087] Hou, L. , S. Guo , Y. Wang , et al. 2021. “Neuropeptide ACP Facilitates Lipid Oxidation and Utilization During Long‐Term Flight in Locusts.” eLife 10: e65279. 10.7554/eLife.65279. 34151772 PMC8324298

[fsn370553-bib-0088] Hou, L. , S. Y. Guo , D. Ding , B. Z. Du , and X. H. Wang . 2022. “Neuroendocrinal and Molecular Basis of Flight Performance in Locusts.” Cellular and Molecular Life Sciences 79: 325. 35644827 10.1007/s00018-022-04344-9 PMC11071871

[fsn370553-bib-0089] Imathiu, S. 2020. “Benefits and Food Safety Concerns Associated With Consumption of Edible Insects.” NFS Journal 18: 1–11. 10.1016/j.nfs.2019.11.002.

[fsn370553-bib-0279] Ishak, S. , and A. Kamari . 2019. “Biodiesel From Black Soldier Fly Larvae Grown on Restaurant Kitchen Waste.” Environmental Chemistry Letters 17: 1143–1150. 10.1007/s10311-018-00844-y.

[fsn370553-bib-0272] Jacqueline, M. B. 2013. “Chapter 6 – Lipids Basics: Fats and Oils in Foods and Health: Healthy Lipid Choices, Roles and Applications in Nutrition.” In Food Science and the Culinary Arts. Culinary Nutrition, edited by J. B. Marcus , vol. 2013, 231–277. Academic Press. 10.1016/B978-0-12-391882-6.00006-6.

[fsn370553-bib-0092] Jajić, I. , A. Popović , M. I. Urošević , et al. 2020. “Fatty and Aminoacid Profile of Mealworms Larvae (*Tenebrio molitor* L.).” Biotechnology in Animal Husbandry 36, no. 2: 167–180.

[fsn370553-bib-0091] Jajić, I. , A. Popović , M. Urošević , S. Krstović , M. Petrović , and D. Guljaš . 2019. “Chemical Composition of Mealworm Larvae ( *Tenebrio molitor* ) Reared in Serbia.” Contemporary Agriculture 68, no. 1–2: 23–27.

[fsn370553-bib-0093] Jayanegara, A. , R. Gustanti , R. Ridwan , and Y. Widyastuti . 2020. “Fatty Acid Profiles of Some Insect Oils and Their Effects on in Vitro Bovine Rumen Fermentation and Methanogenesis.” Italian Journal of Animal Science 19, no. 1: 1310–1317. 10.1080/1828051X.2020.1841571.

[fsn370553-bib-0095] Jongema, Y. 2017. “List of Edible Insects of the World [Online].” http://www.wur.nl/en/Expertise‐Services/Chair‐groups/Plant‐Sciences/Laboratory‐of‐Entomology/Edible‐insects/Worldwide‐species‐list.htm.

[fsn370553-bib-0096] Jucker, C. , D. Lupi , C. D. Moore , M. G. Leonardi , and S. Savoldelli . 2020. “Nutrient Recapture From Insect Farm Waste: Bioconversion With *Hermetia illucens* (L.) (Diptera: Stratiomyidae).” Sustainability 12, no. 1: 362. 10.3390/su12010362.

[fsn370553-bib-0097] Kaczmarek, A. , and M. Boguś . 2021. “The Metabolism and Role of Free Fatty Acids in Key Physiological Processes in Insects of Medical, Veterinary and Forensic Importance.” PeerJ 9: e12563. 10.7717/peerj.12563. 35036124 PMC8710053

[fsn370553-bib-0291] Kasumyan, A. O. 2018. “Olfaction and Gustation in Acipenseridae, With Special References to the Siberian Sturgeon.” In The Siberian sturgeon (Acipenser baerii, Brandt 1869) Volume 1—Biology, edited by P. Williot , G. Nonnotte , D. Vizziano‐Cantonnet , and D. Chebanov , vol. 1, 1st ed., 173–205. Springer.

[fsn370553-bib-0250] Kelemu, S. , S. Niassy , B. Torto , et al. 2015. “African Edible Insects for Food and Feed: Inventory, Diversity, Commonalities and Contribution to Food Security.” Journal of Insects as Food and Feed 1, no. 2: 103–120. 10.3920/JIFF2014.0016.

[fsn370553-bib-0098] Khalil, R. M. 2018. “Locust (*Schistocerca gregaria*) as an Alternative Source of Protein Compared With Other Conventional Protein Sources.” Doctoral dissertation, Sudan University of Science and Technology. http://repository.sustech.edu/handle/123456789/21581.

[fsn370553-bib-0295] Khatun, J. , T. C. Loh , H. Akit , H. L. Foo , and R. Mohamad . 2018. “Influence of Different Sources of Oil on Performance, Meat Quality, Gut Morphology, Ileal Digestibility and Serum Lipid Profile in Broilers.” Journal of Applied Animal Research 46: 479–485. 10.1080/09712119.2017.1337580.

[fsn370553-bib-0099] Kierończyk, B. , M. Rawski , A. Józefiak , et al. 2018. “Effects of Replacing Soybean Oil With Selected Insect Fats on Broilers.” Animal Feed Science and Technology 240: 170–183.

[fsn370553-bib-0100] Kim, B. , H. T. Bang , J. Y. Jeong , et al. 2021. “Effects of Dietary Supplementation of Black Soldier Fly ( *Hermetia illucens* ) Larvae Oil on Broiler Health.” Journal of Poultry Science 58, no. 4: 222–229. 10.2141/jpsa.0200070. PMC8630405 34899017

[fsn370553-bib-0101] Kim, B. , H. T. Bang , K. H. Kim , et al. 2020. “Evaluation of Black Soldier Fly Larvae Oil as a Dietary Fat Source in Broiler Chicken Diets.” Journal of Animal Science and Technology 62: 187–197. 32292926 10.5187/jast.2020.62.2.187 PMC7142286

[fsn370553-bib-0102] Kim, J. H. , E. Y. Kim , and K. J. Chung . 2021. “Mealworm Oil (MWO) Enhances Wound Healing Potential Through the Activation of Fibroblast and Endothelial Cells.” Molecules 26: 779–790. 33546205 10.3390/molecules26040779 PMC7913324

[fsn370553-bib-0103] Kim, T. , H. I. Yong , Y. Kim , H. Kim , and H. Paik . 2019. “Edible Insects as a Protein Source: A Review of Public Perception, Processing Technology, and Research Trends.” Food Science of Animal Resources 39, no. 4: 521–540. 10.5851/kosfa.2019.e53. 31508584 PMC6728817

[fsn370553-bib-0104] Kim, Y. B. , D.‐H. Kim , S.‐B. Jeong , et al. 2020. “Black Soldier Fly Larvae Oil as an Alternative Fat Source in Broiler Nutrition.” Poultry Science 99, no. 6: 3133–3143. 10.1016/j.psj.2020.01.018. PMC7597637 32475450

[fsn370553-bib-0261] Kinyuru, J. N. , S. O. Konyole , N. Roos , et al. 2013. “Nutrient Composition of Four Species of Winged Termites Consumed in Western Kenya.” Journal of Food Composition and Analysis 30, no. 2: 120–124. 10.1016/j.jfca.2013.02.008.

[fsn370553-bib-0286] Kinyuru, J. N. U. 2014. “Nutrient Composition and Utilization of Edible Termites (*Macrotermes subhylanus*) and Grasshoppers (*Ruspolia differenes*) From Lake Victoria Region of Kenya (Doctoral dissertation).” http://ir.jkuat.ac.ke/handle/123456789/1407.

[fsn370553-bib-0105] Kinyuru, J. N. , J. B. Mogendi , C. Riwa , and N. S. Ndung'u . 2015. “Edible Insects—A Novel Source of Essential Nutrients for Human Diet: Learning From Traditional Knowledge.” Animal Frontiers 5, no. 2: 14–19. 10.2527/af.2015-0014.

[fsn370553-bib-0263] Köhler, R. , A. Irias‐Mata , E. Ramandey , et al. 2020. “Nutrient Composition of the Indonesian Sago Grub (*Rhynchophorus bilineatus*).” International Journal of Tropical Insect Science 40: 677–686. 10.1007/s42690-020-00120-z.

[fsn370553-bib-0106] Kolobe, S. D. , T. G. Manyelo , E. Malematja , N. A. Sebola , and M. Mabelebele . 2023. “Fats and Major Fatty Acids Present in Edible Insects Utilised as Food and Livestock Feed.” Veterinary and Animal Science 22: 100312. 10.1016/j.vas.2023.100312. 37736572 PMC10509705

[fsn370553-bib-0107] Kourimská, L. , and A. Adámková . 2016. “Nutritional and Sensory Quality of Edible Insects.” NFS Journal 4: 22–26. 10.1016/j.nfs.2016.07.001.

[fsn370553-bib-0108] Kumar, S. P. J. , S. R. Prasad , R. Banerjee , D. K. Agarwal , K. S. Kulkarni , and K. V. Ramesh . 2017. “Green Solvents and Technologies for Oil Extraction From Oilseeds.” Chemistry Central Journal 11: 28123451. 10.1186/s13065-017-0238-8 PMC5258651 28123451

[fsn370553-bib-0109] Lähteenmäki ‐Uutela, A. , S. B. H'enault‐Ethier , S. Marimuthu , et al. 2018. “The Impact of the Insect Regulatory System on the Insect Marketing System.” Journal of Insects as Food and Feed 4: 187–198.

[fsn370553-bib-0110] Lahteenmaki‐Uutela, A. , S. B. Marimuthu , and N. Meijer . 2021. “Regulations on Insects as Food and Feed: A Global Comparison.” Journal of Insects as Food and Feed 7: 849–856.

[fsn370553-bib-0111] Lai, E. P. C. 2014. “Biodiesel: Environmental Friendly Alternative to Petrodiesel.” Journal of Petroleum and Environmental Biotechnology 5: 122.

[fsn370553-bib-0112] Lakshimi, V. I. , and M. Kavitha . 2023. “New Insights Into Prospective Health Potential of ω‐3 PUFAs.” Current Nutrition Reports 12, no. 4: 813–829. 10.1007/s13668-023-00508-6. 37996669

[fsn370553-bib-0113] Lange, K. W. , and Y. Nakamura . 2021. “Edible Insects as Future Food: Chances and Challenges.” Journal of Future Foods 1, no. 1: 38–46. 10.1016/j.jfutfo.2021.10.001.

[fsn370553-bib-0114] Lange, K. W. , and Y. Nakamura . 2023. “Potential Contribution of Edible Insects to Sustainable Consumption and Production.” Frontiers in Sustainability 4: 1112950. 10.3389/frsus.2023.1112950.

[fsn370553-bib-0115] Laroche, M. , V. Perreault , A. Marciniak , A. Gravel , J. Chamberland , and A. Doyen . 2019. “Comparison of Conventional and Sustainable Lipid Extraction Methods for the Production of Oil and Protein Isolate From Edible Insect Meal.” Food 8: 572. 10.3390/foods8110572. PMC6915342 31766306

[fsn370553-bib-0116] Latif, S. , F. Anwar , A. I. Hussain , and M. Shahid . 2011. “Aqueous Enzymatic Process for Oil and Protein Extraction From *Moringa oleifera* Seed.” European Journal of Lipid Science and Technology 113: 1012–1018.

[fsn370553-bib-0117] Lease, H. M. , and B. O. Wolf . 2011. “Lipid Content of Terrestrial Arthropods in Relation to Body Size, Phylogeny, Ontogeny and Sex.” Physiological Entomology 36: 29–38. 10.1111/j.1365-3032.2010.00767.x.

[fsn370553-bib-0118] Lee, D. J. , M. Kim , and S. Jung . 2022. “Direct Conversion of Yellow Mealworm Larvae Into Biodiesel via a Non‐Catalytic Transesterification Platform.” Chemical Engineering Journal 427: 131782.

[fsn370553-bib-0121] Lehtovaara, V. J. , A. Valtonen , J. Sorjonen , et al. 2017. “The Fatty Acid Contents of the Edible Grasshopper *Ruspolia differens* Can Be Manipulated Using Artificial Diets.” Journal of Insects as Food and Feed 3, no. 4: 253–262.

[fsn370553-bib-0275] Lenaerts, S. , M. Van Der Borght , A. Callens , and L. Van Campenhout . 2018. “(2018). Suitability of Microwave Drying for Mealworms (*Tenebrio molitor*) as Alternative to Freeze Drying: Impact on Nutritional Quality and Colour.” Food Chemistry 254: 129–136. 10.1016/j.foodchem.2018.02.006. 29548432

[fsn370553-bib-0123] Li, S. , H. Ji , B. Zhang , J. Tian , J. Zhou , and H. Yu . 2016. “Influence of Black Soldier Fly ( *Hermetia illucens* ) Larvae Oil on Growth Performance, Body Composition, Tissue Fatty Acid Composition and Lipid Deposition in Juvenile Jian Carp ( *Cyprinus carpio* Var. Jian).” Aquaculture 465: 43–52.

[fsn370553-bib-0124] Li, T. H. , C. R. Zhang , P. F. Che , Y. Ma , and L. S. Zang . 2021. “Recycling of Spent Mushroom Substrate and Food Waste: Utilisation as Feed Materials for Black Soldier Fly (*Hermetia illucens* (L.) Diptera: Stratiomyidae).” Journal of Insects as Food and Feed 7: 409–417. 10.3920/JIFF2020.0105.

[fsn370553-bib-0284] Li, Q. , L. Zheng , N. Qiu , H. Cai , J. K. Tomberlin , and Y. Ziniu . 2011. “Bioconversion of Dairy Manure by Black Soldier Fly (Diptera: Stratiomyidae) for Biodiesel and Sugar Production.” Waste Management 31, no. 6: 1316–1320. 10.1016/j.wasman.2011.01.005. 21367596

[fsn370553-bib-0125] Li, X. , Y. Zhou , and K. Wu . 2023. “Biological Characteristics and Energy Metabolism of Migrating Insects.” Metabolites 13, no. 3: 439. 10.3390/metabo13030439. 36984878 PMC10055822

[fsn370553-bib-0307] Liland, N. S. , I. Biancarosa , P. Araujo , et al. 2017. “Modulation of Nutrient Composition of Black Soldier fly (*Hermetia illucens*) Larvae by Feeding Seaweed‐Enriched Media.” PLoS One 12, no. 8: e0183188. 10.1371/journal.pone.0183188. 28837591 PMC5570497

[fsn370553-bib-0126] Lin, X. , F. Wang , Y. Lu , et al. 2023. “A Review on Edible Insects in China: Nutritional Supply, Environmental Benefits, and Potential Applications.” Current Research in Food Science 7: 100596. 10.1016/j.crfs.2023.100596. 37744556 PMC10517268

[fsn370553-bib-0127] Liya, Y. 2015. “A Study on the Potential of Insect Protein and Lipid as a Food Source.” Wageningen University and Research ProQuest Dissertations Publishing, 28230080.

[fsn370553-bib-0128] Longvah, T. , K. Mangthya , and P. Ramulu . 2011. “Nutrient Composition and Protein Quality Evaluation of Eri Silkworm (*Samia ricinii*) Prepupae and Pupae.” Food Chemistry 128, no. 2: 400–403. 10.1016/j.foodchem.2011.03.041. 25212147

[fsn370553-bib-0129] Loponte, R. , S. Nizza , F. Bovera , et al. 2017. “Growth Performance, Blood Profiles and Carcass Traits of Barbary Partridge (*Alectoris barbara*) Fed Two Different Insect Larvae Meals (*Tenebrio molitor* and *Hermetia illucens*).” Research in Veterinary Science 115: 183–188. 28472736 10.1016/j.rvsc.2017.04.017

[fsn370553-bib-0130] Lorrette, B. , and L. Sanchez . 2022. “New Lipid Sources in the Insect Industry, Regulatory Aspects and Applications.” OCL 29: 22. 10.1051/ocl/2022017.

[fsn370553-bib-0131] Losey, J. E. , and M. Vaughan . 2006. “The Economic Value of Ecological Services Provided by Insects.” Bioscience 56, no. 4: 311–323.

[fsn370553-bib-0132] Lu, K. , X. Chen , Y. Li , W. Li , and Q. Zhou . 2018. “Lipophorin Receptor Regulates *Nilaparvata lugens* Fecundity by Promoting Lipid Accumulation and Vitellogenin Biosynthesis.” Comparative Biochemistry and Physiology ‐Part a: Molecular and Integrative Physiology 219‐220: 28–37. 10.1016/j.cbpa.2018.02.008. 29476823

[fsn370553-bib-0133] Lückstädt, W. , and C. Panknin . 2016. “Antimicrobial Activity of Black Soldier Fly Larvae (*Hermetia illucens*) Against Pathogenic Bacteria in Aquaculture.” Aquaculture 456: 225–232.

[fsn370553-bib-0134] Makkar, H. P. S. , G. Tran , V. Heuze , and P. Ankers . 2014. “State‐Of‐The‐Art on Use of Insects as Animal Feed.” Animal Feed Science and Technology 197: 1–33.

[fsn370553-bib-0271] Mancini, A. , E. Imperlini , E. Nigro , et al. 2015. “Biological and Nutritional Properties of Palm Oil and Palmitic Acid: Effects on Health.” Molecules 20, no. 9: 17339–17361. 26393565 10.3390/molecules200917339 PMC6331788

[fsn370553-bib-0135] Mangas‐Sánchez, J. , and P. Adlercreutz . 2015. “Highly Efficient Enzymatic Biodiesel Production Promoted by Particle‐Induced Emulsification.” Biotechnology for Biofuels 8: 58. 25873996 10.1186/s13068-015-0247-6 PMC4396811

[fsn370553-bib-0278] Manzano‐Agugliaro, F. , M. J. Sanchez‐Muros , F. G. Barroso , A. Martínez‐Sánchez , S. Rojo , and C. Pérez‐Bañón . 2012. “Insects for Biodiesel Production.” Renewable and Sustainable Energy Reviews 16, no. 6: 3744–3753. 10.1016/j.rser.2012.03.017.

[fsn370553-bib-0247] Marcucci, C. 2020. “Food Frontiers: Insects as Food, Is the Future Already here? Mediterranean.” Journal of Nutrition and Metabolism 13, no. 1: 43–52. 10.3233/MNM-190348.

[fsn370553-bib-0288] Mariod, A. A. 2020. “The Legislative Status of Edible Insects in the World.” In African Edible Insects as Alternative Source of Food, Oil, Protein and Bioactive Components, edited by A. A. Mariod , 141–148. Springer International Publishing.

[fsn370553-bib-0136] Mariod, A. A. 2013. “Insect Oil and Protein: Biochemistry, Food and Other Uses: Review.” Agricultural Sciences 4, no. 9B: 76–80. 10.4236/as.2013.49B013.

[fsn370553-bib-0254] Mariod, A. A. , S. I. Abdel‐Wahab , and N. M. Ain . 2011. “Proximate Amino Acid, Fatty Acid and Mineral Composition of Two Sudanese Edible Pentatomid Insects.” International Journal of Tropical Insect Science 31: 145–153. 10.1017/S1742758411000282.

[fsn370553-bib-0137] Marusich, E. , H. Mohamed , Y. Afanasev , and S. Leonov . 2020. “Fatty Acids From *Hermetia illucens* Larvae Fat Inhibit the Proliferation and Growth of Actual Phytopathogens.” Microorganisms 8: 1423–1443. 32948050 10.3390/microorganisms8091423 PMC7563668

[fsn370553-bib-0138] Mat Yusoff, M. , M. H. Gordon , and K. Niranjan . 2015. “Aqueous Enzyme Assisted Oil Extraction From Oilseeds and Emulsion de‐Emulsifying Methods: A Review.” Trends in Food Science and Technology 41: 60–82.

[fsn370553-bib-0139] Mattioli, S. , F. Fratini , C. Cacchiarelli , et al. 2024. “Chemical Composition, Fatty Acid Profile, Antioxidant Content, and Microbiological Loads of Lesser Mealworm, Mealworm, and Superworm Larvae.” Italian Journal of Animal Science 23, no. 1: 125–137. 10.1080/1828051X.2023.2293856.

[fsn370553-bib-0140] Mentang, F. , M. Maita , H. Ushio , and T. Ohshima . 2011. “Efficacy of Silkworm (*Bombyx mori* L.) Chrysalis Oil as a Lipid Source in Adult Wistar Rats.” Food Chemistry 127: 899–904. 10.1016/j.foodchem.2011.01.045. 25214076

[fsn370553-bib-0141] Meschi, E. , and R. Delanoue . 2021. “Adipokine and Fat Body in Flies: Connecting Organs.” Molecular and Cellular Endocrinology 533: 111339. 10.1016/j.mce.2021.111339. 34082046

[fsn370553-bib-0142] Meyer‐Rochow, V. B. , R. T. Gahukar , S. Ghosh , and C. Jung . 2021. “Chemical Composition, Nutrient Quality and Acceptability of Edible Insects Are Affected by Species, Developmental Stage, Gender, Diet, and Processing Method.” Food 10: 1036. 10.3390/foods10051036. PMC8150737 34068654

[fsn370553-bib-0143] Mishyna, M. , and M. Glumac . 2021. “So Different, Yet So Alike Pancrustacea: Health Benefits of Insects and Shrimps.” Journal of Functional Foods 76: 104316. 10.1016/j.jff.2020.104316.

[fsn370553-bib-0144] Mishyna, M. , J. K. Keppler , and J. Chen . 2021. “Techno‐Functional Properties of Edible Insect Proteins and Effects of Processing.” Current Opinion in Colloid & Interface Science 56: 101508. 10.1016/j.cocis.2021.101508.

[fsn370553-bib-0145] Mlcek, J. , A. Adamkova , M. Adamek , M. Borkovcova , M. Bednarova , and I. Knizkova . 2019. “Fat From Tenebrionidae Bugs ‐ Sterols Content, Fatty Acid Profiles, and Cardiovascular Risk Indexes.” Polish Journal of Food and Nutrition Sciences 69, no. 3: 247–254. 10.31883/pjfns/109666.

[fsn370553-bib-0146] Mohamed, H. , E. Marusich , Y. Afanasev , and S. Leonov . 2022. “Bacterial Outer Membrane Permeability Increase Underlies the Bactericidal Effect of Fatty Acids From *Hermetia illucens* (Black Soldier Fly) Larvae Fat Against Hypermucoviscous Isolates of *Klebsiella pneumoniae* .” Frontiers in Microbiology 13: 844811. 10.3389/fmicb.2022.844811. 35602017 PMC9121012

[fsn370553-bib-0147] Monter‐Miranda, J. , J. M. T. P. B. Zamudio‐Flores , F. J. M. E. O. C. Rios‐Velasco , F. H. M. Hernandez‐Gonzalez , and H. Y. L. De La Peña . 2018. “Nutritional Characterization of Fatty Acids and Minerals in *Brachystola magna* (Girard) During Their Development.” Emirates Journal of Food and Agriculture 30: 389. 10.9755/ejfa.2018.v30.i5.1682.

[fsn370553-bib-0149] Mudalungu, C. M. , H. O. Mokaya , and C. M. Tanga . 2023. “Beneficial Sterols in Selected Edible Insects and Their Associated Antibacterial Activities.” Scientific Reports 13: 10786. 10.1038/s41598-023-37905-4. 37402875 PMC10319837

[fsn370553-bib-0150] Munthali, M. G. , L. Chilora , M. Goliath , et al. 2023. “The Economic Cost‐benefit Analysis of Black Soldier Fly as an Alternative Animal and Fish Feed Ingredient in Malawi.” MwAPATA Institute Working Paper No. 23/01.

[fsn370553-bib-0151] Murefu, T. R. , L. Macheka , R. Musundire , and F. A. Manditsera . 2019. “Safety of Wild Harvested and Reared Edible Insects: A Review.” Food Control 101: 209–224. 10.1016/j.foodcont.2019.03.003.

[fsn370553-bib-0287] Musundire, R. , I. M. Osuga , X. Cheseto , J. Irungu , and B. Torto . 2016. “Aflatoxin Contamination Detected in Nutrient and Anti‐Oxidant Rich Edible Stink Bug Stored in Recycled Grain Containers.” PLoS One 11, no. 1: e0145914. 10.1371/journal.pone.0145914. 26731419 PMC4701502

[fsn370553-bib-0152] Nelson, D. R. , T. P. Freeman , and J. S. Buckner . 2000. “Waxes and Lipids Associated With the External Waxy Structures of Nymphs and Pupae of the Giant Whitefly, Aleurodicus Dugesii.” Comparative Biochemistry and Physiology. Part B, Biochemistry and Molecular Biology 125, no. 2: 265–278. 10.1016/s0305-0491(99)00177-7. 10817914

[fsn370553-bib-0153] Nelson, D. R. , T. P. Freeman , J. S. Buckner , K. A. Hoelmer , C. G. Jackson , and J. R. Hagler . 2003. “Characterization of the Cuticular Surface Wax Pores and the Waxy Particles of the Dustywing, *Semidalis flinti* (Neuroptera: Coniopterygidae).” Comparative Biochemistry and Physiology Part B: Biochemistry and Molecular Biology 136, no. 2: 343–356. 10.1016/S1096-4959(03)00216-1. 14529760

[fsn370553-bib-0154] Nestel, D. , N. T. Papadopoulos , C. Pascacio‐Villafán , N. Righini , A. R. Altuzar‐Molina , and M. Aluja . 2016. “Resource Allocation and Compensation During Development in Holometabolous Insects.” Journal of Insect Physiology 95: 78–88. 10.1016/j.jinsphys.2016.09.010. 27650504

[fsn370553-bib-0283] Nguyen, H. D. , M. H. Thi Nguyen , T. D. Nguyen , and P. T. Nguyen . 2016. “ *Nephelium lappaceum* Oil: A Low‐Cost Alternative Feedstock for Sustainable Biodiesel Production Using Magnetic Solid Acids.” Environmental Progress & Sustainable Energy 35: 603–610.

[fsn370553-bib-0155] Nowak, V. , D. Persijn , D. Rittenschober , and U. R. Charrondiere . 2016. “Review of Food Composition Data for Edible Insects.” Food Chemistry 193: 39–46. 10.1016/j.foodchem.2014.10.114. 26433285

[fsn370553-bib-0305] Nowakowski, A. C. , A. C. Miller , M. E. Miller , H. Xiao , and X. Wu . 2022. “Potential Health Benefits of Edible Insects.” Critical Reviews in Food Science and Nutrition 62, no. 13: 3499–3508. 10.1080/10408398.2020.1867053. 33397123

[fsn370553-bib-0156] Nyangena, D. M. , C. Mutungi , S. Imathiu , et al. 2020. “Effects of Traditional Processing Techniques on the Nutritional and Microbiological Quality of Four Edible Insect Species Used for Food and Feed in East Africa.” Food 9, no. 5: 574. 10.3390/foods9050574. PMC7278588 32375385

[fsn370553-bib-0157] Ohler, K. , V. C. Schreiner , D. Martin‐Creuzburg , and R. B. Schäfer . 2023. “Trophic Transfer of Polyunsaturated Fatty Acids Across the Aquatic‐Terrestrial Interface: An Experimental Tritrophic Food Chain Approach.” Ecology and Evolution 13, no. 3: e9927. 10.1002/ece3.9927. 36969929 PMC10037435

[fsn370553-bib-0256] Otero, P. , A. Gutierrez‐Docio , J. N. Del Hierro , G. Reglero , and D. Martin . 2020. “Extracts From the Edible Insects *Acheta domesticus* and *Tenebrio molitor* With Improved Fatty Acid Profile due to Ultrasound Assisted or Pressurized Liquid Extraction.” Food Chemistry 314: 126200. 10.1016/j.foodchem.2020.126200. 31972408

[fsn370553-bib-0267] Oyarzun, S. E. , G. J. Crawshaw , and E. V. Valdes . 1996. “Nutrition of the Tamandua: I. Nutrient Composition of Termites (*Nasutitermes* spp.) and Stomach Contents From Wild Tamanduas (*Tamandua tetradactyla*).” Zoo Biology 15: 509–524.

[fsn370553-bib-0163] Pali‐Schöll, I. , P. Meinlschmidt , D. Larenas‐Linnemann , et al. 2019. “Edible Insects: Cross‐Recognition of IgE From Crustacean‐ and House Dust Mite Allergic Patients, and Reduction of Allergenicity by Food Processing.” World Allergy Organization Journal 12, no. 1: 100006. 10.1016/j.waojou.2018.10.001. 30937131 PMC6439408

[fsn370553-bib-0164] Palm, W. , and J. Rodenfels . 2020. “Understanding the Role of Lipids and Lipoproteins in Development.” Development (Cambridge, England) 147, no. 24: dev186411. 10.1242/dev.186411. 33355243

[fsn370553-bib-0165] Paoletti, M. G. , E. Buscardo , D. J. Vanderjagt , et al. 2003. “Nutrient Content of Termites (Syntermes Soldiers) Consumed Bymakiritare Amerindians of the Altoorinoco of Venezuela.” Ecology of Food and Nutrition 42, no. 2: 177–191. 10.1080/036702403902-2255177.

[fsn370553-bib-0166] Park, J.‐Y. , S. Jung , Y.‐G. Na , et al. 2022. “Biodiesel Production From the Black Soldier Fly Larvae Grown on Food Waste and Its Fuel Property Characterization as a Potential Transportation Fuel.” Environmental Engineering Research 27, no. 3: 200704. 10.4491/eer.2020.704.

[fsn370553-bib-0167] Paul, A. , M. Frederich , R. C. Megido , et al. 2017. “Insect Fatty Acids: A Comparison of Lipids From Three Orthopterans and *Tenebrio molitor* L. Larvae.” Journal of Asia‐Pacific Entomology 20: 337–340.

[fsn370553-bib-0168] Pilco‐Romero, G. , A. M. Chisaguano‐Tonato , E. M. Herrera‐Fontana , et al. 2023. “House Cricket (*Acheta domesticus*): A Review Based on Its Nutritional Composition, Quality, and Potential Uses in the Food Industry.” Trends in Food Science and Technology 142: 104226. 10.1016/j.tifs.2023.104226.

[fsn370553-bib-0169] Pomeranz, Y. 2013. Food Analysis: Theory and Practice. Springer Science & Business Media.

[fsn370553-bib-0170] Poyraz, E. , E. Göncü , and O. Parlak . 2013. “Investigation of the Interaction Between Fat Body and Ovary Development During Pupal Transformation in Silkworm, *Bombyx mori* .” IUFS Journal of Biology 72, no. 2: 1–13.

[fsn370553-bib-0171] Preece, K. E. , N. Hooshyar , and N. J. Zuidam . 2017. “Whole Soybean Protein Extraction Processes: A Review.” Innovative Food Science and Emerging Technologies 43: 163–172.

[fsn370553-bib-0172] Purschke, B. , T. Stegmann , M. Schreiner , and H. Jager . 2017. “Pilot‐Scale Supercritical CO2 Extraction of Edible Insect Oil From *Tenebrio molitor* L. Larvae—Influence of Extraction Conditions on Kinetics, Defatting Performance and Compositional Properties.” European Journal of Lipid Science and Technology 119: 1600134.

[fsn370553-bib-0302] Pyo, S. J. , D. G. Kang , C. Jung , and H. Y. Sohn . 2020. “Anti‐Thrombotic, Anti‐Oxidant and Haemolysis Activities of Six Edible Insect Species.” Food 9: 401. 10.3390/foods9040401. PMC7231258 32244589

[fsn370553-bib-0173] Ragossnig, H. A. , and A. M. Ragossnig . 2021. “Biowaste Treatment Through Industrial Insect Farms: One Bioeconomy Puzzle Piece Towards a Sustainable Net‐Zero Carbon Economy?” Waste Management and Research 39, no. 8: 1005–1006. 10.1177/0734242x211036949. 34338094

[fsn370553-bib-0249] Raheem, D. , C. Carrascosa , O. B. Oluwole , et al. 2019. “Traditional Consumption of and Rearing Edible Insects in Africa, Asia and Europe.” Critical Reviews in Food Science and Nutrition 59, no. 14: 2169–2188. 10.1080/10408398.2018.1440191. 29446643

[fsn370553-bib-0174] Rai, P. K. , S. S. Lee , M. Zhang , Y. F. Tsang , and K. H. Kim . 2019. “Heavy Metals in Food Crops: Health Risks, Fate, Mechanisms, and Management.” Environment International 125: 365–385. 10.1016/j.envint.2019.01.067. 30743144

[fsn370553-bib-0175] Rajapakse, S. , D. Qu , A. S. Ahmed , J. Rickers‐Haunerland , and N. H. Haunerland . 2019. “Effects of FABP Knockdown on Flight Performance of the Desert Locust, Schistocerca Gregaria.” Journal of Experimental Biology 222, no. Pt 21: jeb203455. 10.1242/jeb.203455. 31597730

[fsn370553-bib-0176] Raksakantong, P. , N. Meeso , J. Kubola , and S. Siriamornpun . 2010. “Fatty Acids and Proximate Composition of Eight Thai Edible Terricolous Insects.” Food Research International 43, no. 1: 350–355.

[fsn370553-bib-0177] Ravzanaadii, N. , S. H. Kim , W. H. Choi , S. J. Hong , and N. J. Kim . 2012. “Nutritional Value of Mealworm, *Tenebrio molitor* as Food Source.” International Journal of Industrial Entomology 25: 93–98.

[fsn370553-bib-0246] Rodríguez‐Miranda, J. , J. P. Alcántar‐Vázquez , T. Zúñiga‐Marroquín , et al. 2019. “Insects as an Alternative Source of Protein: A Review of the Potential Use of Grasshopper (*Sphenarium purpurascens* Ch.) as a Food Ingredient.” European Food Research and Technology 245: 2613–2620. 10.1007/s00217-019-03383-0.

[fsn370553-bib-0179] Rumbos, C. I. , and C. G. Athanassiou . 2021. “The Superworm, Zophobas Morio (Coleoptera:Tenebrionidae): A ‘Sleeping Giant’ in Nutrient Sources.” Journal of Insect Science 21, no. 2: 1–11. 10.1093/jisesa/ieab014. PMC8033247 33834209

[fsn370553-bib-0181] Rumpold, B. A. , and O. K. Schlüter . 2013a. “Nutritional Composition and Safety Aspects of Edible Insects.” Molecular Nutrition and Food Research 57, no. 5: 802–823. 10.1002/mnfr.201200735. 23471778

[fsn370553-bib-0180] Rumpold, B. A. , and O. K. Schlüter . 2013b. “Potential and Challenges of Insects as an Innovative Source for Food and Feed Production.” Innovative Food Science and Emerging Technologies 17: 1–11.

[fsn370553-bib-0182] Sabolová, M. , A. Adámková , L. Kouřimská , D. Chrpová , and J. Pánek . 2016. “Minor Lipophilic Compounds in Edible Insects.” Potravinarstvo Slovak Journal of Food Sciences 10, no. 1: 400–406. 10.5219/605.

[fsn370553-bib-0183] Saitou, N. , and M. Nei . 1987. “The Neighbor‐Joining Method: A New Method for Reconstructing Phylogenetic Trees.” Molecular Biology and Evolution 4: 406–425. 3447015 10.1093/oxfordjournals.molbev.a040454

[fsn370553-bib-0285] Salomone, R. , G. Saija , G. Mondello , A. Giannetto , S. Fasulo , and D. Savastano . 2017. “Savastano Environmental Impact of Food Waste Bioconversion by Insects: Application of Life Cycle Assessment to Process Using *Hermetia illucens* .” Journal of Cleaner Production 140: 890–905.

[fsn370553-bib-0264] Sánchez‐Muros, M. J. , F. G. Barroso , and F. Manzano‐Agugliaro . 2014. “Insect Meal as Renewable Source of Food for Animal Feeding: A Review.” Journal of Cleaner Production 65: 16–27. 10.1016/j.jclepro.2013.11.068.

[fsn370553-bib-0184] Sandoval‐Insausti, H. , R. F. Perez‐Tasigchana , E. Lopez‐Garcia , E. Garcia‐Esquinas , F. Rodriguez‐Artalejo , and P. Guallar‐Castillon . 2016. “Macronutrients Intake and Incident Frailty in Older Adults: A Prospective Cohort Study.” Journals of Gerontology. Series A, Biological Sciences and Medical Sciences 71: 1329–1334. 26946103 10.1093/gerona/glw033

[fsn370553-bib-0185] Sangduan, C. 2018. “Skincare Product Containing *Hermetia illucens* Extract.” U.S. Patent Application No 15/981,689.

[fsn370553-bib-0186] Saviane, A. , L. Tassoni , D. Naviglio , et al. 2021. “Mechanical Processing of *Hermetia illucens* Larvae and *Bombyx mori* Pupae Allows to Obtain Oil With Antimicrobial Activity.” Animals 11: 783. 10.3390/ani11030783. 33799904 PMC8001418

[fsn370553-bib-0293] Schiavone, A. , M. Cullere , M. De Marco , et al. 2016. “Partial or Total Replacement of Soybean Oil by Black Soldier Fly Larvae (*Hermetia illucens* L.) Fat in Broiler Diets: Effect on Growth Performances, Feed‐Choice, Blood Traits, Carcass Characteristics and Meat Quality.” Italian Journal of Animal Science 16, no. 1: 93–100. 10.1080/1828051X.2016.1249968.

[fsn370553-bib-0187] Schiavone, A. , S. Dabbou , M. De Marco , et al. 2018. “Black Soldier Fly Larva Fat Inclusion in Finisher Broiler Chicken Diet as an Alternative Fat Source.” Animal 12: 2032–2039. 10.1017/S1751731117003743. 29343316

[fsn370553-bib-0188] Selaledi, L. A. , C. A. Mbajiorgu , and M. Mabelebele . 2020. “The Use of Yellow Mealworm (*T. molitor*) as Alternative Source of Protein in Poultry Diets: A Review.” Tropical Animal Health and Production 52, no. 1: 7–16. 10.1007/s11250-019-02033-7. 31392553

[fsn370553-bib-0253] Sen, Y. , L. Qing , G. Yang , Z. Longyu , and L. Ziduo . 2014. “Biodiesel Production From Swine Manure via Housefly Larvae (*Musca domestica* L.).” Renewable Energy 66: 222–227. 10.1016/j.renene.2013.11.076.

[fsn370553-bib-0189] Seni, A. 2017. “Edible Insects: Future Prospects for Dietary Regimen.” International Journal of Current Microbiology and Applied Sciences 6, no. 8: 1302–1314. 10.20546/ijcmas.2017.608.158.

[fsn370553-bib-0303] Seo, M. , T. W. Goo , M. Y. Chung , et al. 2017. “ *Tenebrio molitor* Larvae Inhibit Adipogenesis Through AMPK and MAPKs Signaling in 3T3‐L1 Adipocytes and Obesity in High‐Fat Diet‐Induced Obese Mice.” International Journal of Molecular Sciences 18, no. 3: 518. 10.3390/ijms18030518. 28264489 PMC5372534

[fsn370553-bib-0190] Siddiqui, S. A. , M. Ghisletta , B. M. Yunusa , et al. 2023. “Grasshoppers and Locusts as Human Foods – A Comprehensive Review.” Journal of Insects as Food and Feed 9, no. 10: 1247–1264. 10.1163/23524588-20230025.

[fsn370553-bib-0191] Sinclair, B. J. , and K. E. Marshall . 2018. “The Many Roles of Fats in Overwintering Insects.” Journal of Experimental Biology 221, no. Suppl_1: jeb161836. 10.1242/jeb.161836. 29514877

[fsn370553-bib-0192] Siow, H. S. , K. Sudesh , P. Murugan , and S. Ganesan . 2021. “Mealworm ( *Tenebrio molitor* ) oil Characterization and Optimization of the Free Fatty Acid Pretreatment via Acid‐Catalyzed Esterification.” Fuel 299: 120905.

[fsn370553-bib-0193] Skowronek, P. , Ł. Wójcik , and A. Strachecka . 2021. “Fat Body‐Multifunctional Insect Tissue.” Insects 12, no. 6: 547. 10.3390/insects12060547. 34208190 PMC8230813

[fsn370553-bib-0194] Smetana, S. , L. Leonhardt , S. M. Kauppi , A. Pajic , and V. Heinz . 2020. “Insect Margarine: Processing, Sustainability and Design.” Journal of Cleaner Production 264: 121670. 10.1016/j.jclepro.2020.121670.

[fsn370553-bib-0255] Son, Y. J. , J. C. Lee , I. K. Hwang , C. W. Nho , and S. H. Kim . 2019. “Physicochemical Properties of Mealworm (*Tenebrio molitor*) Powders Manufactured by Different Industrial Processes.” LWT – Food Science and Technology 116: 108514.

[fsn370553-bib-0197] Stabler, D. , M. Al‐Esawy , J. A. Chennells , G. Perri , A. Robinson , and G. A. Wright . 2021. “Regulation of Dietary Intake of Protein and Lipid by Nurse‐Age Adult Worker Honey Bees.” Journal of Experimental Biology 224, no. 3: jeb230615. 10.1242/jeb.230615. 33443043 PMC7888720

[fsn370553-bib-0301] Stajić, S. , D. Živković , M. Perunović , S. Šobajić , and D. Vranić . 2011. “Cholesterol Content and Atherogenicity of Fermented Sausages Made of Pork Meat From Various Breeds.” Procedia Food Science 1: 568–575.

[fsn370553-bib-0198] Stork, N. E. 2018. “How Many Species of Insects and Other Terrestrial Arthropods Are There on Earth?” Annual Review of Entomology 63: 31–45. 10.1146/annurev-ento-020117-043348. 28938083

[fsn370553-bib-0199] Stull, V. J. , and T. L. Weir . 2023. “Chitin and Omega‐3 Fatty Acids in Edible Insects Have Underexplored Benefits for the Gut Microbiome and Human Health.” Nature Food 4: 283–287. 10.1038/s43016-023-00728-7. 37117549

[fsn370553-bib-0282] Su, C. H. , H. C. Nguyen , T. L. Bui , and D. L. Huang . 2019. “Enzyme‐Assisted Extraction of Insect Fat for Biodiesel Production.” Journal of Cleaner Production 223: 436–444. 10.1016/j.jclepro.2019.03.150.

[fsn370553-bib-0200] Surendra, K. C. , R. Olivier , J. K. Tomberlin , R. Jha , and S. K. Khanal . 2016. “Bioconversion of Organic Wastes Into Biodiesel and Animal Feed via Insect Farming.” Renewable Energy 98: 197–202. 10.1016/j.renene.2016.03.022.

[fsn370553-bib-0201] Tafi, E. 2022. “Use of the Black Soldier Fly *Hermetia illucens* L. (Diptera: Stratiomyidae) as an Alternative Source of Chitin and Chitosan for the Production of Biopolymeric Films in Agro‐Food Applications.” Thesis dessertation. https://hdl.handle.net/11563/154465.

[fsn370553-bib-0202] Tamura, K. , G. Stecher , and S. Kumar . 2021. “MEGA 11: Molecular Evolutionary Genetics Analysis Version 11.” Molecular Biology and Evolution 38: 3022–3027. 10.1093/molbev/msab120. 33892491 PMC8233496

[fsn370553-bib-0204] Tan, K. W. M. , and Y. K. Lee . 2016. “The Dilemma for Lipid Productivity in Green Microalgae: Importance of Substrate Provision in Improving Oil Yield Without Sacrificing Growth.” Biotechnology for Biofuels 9: 255. 27895709 10.1186/s13068-016-0671-2 PMC5120525

[fsn370553-bib-0205] Tang, C. , D. Yang , H. Liao , et al. 2019. “Edible Insects as a Food Source: A Review.” Food Production, Processing and Nutrition 1: 8. 10.1186/s43014-019-0008-1.

[fsn370553-bib-0206] Tao, J. , and Y. Li . 2018. “Edible Insects as a Means to Address Global Malnutrition and Food Insecurity Issues.” Food Quality and Safety 2, no. 1: 17–26. 10.1093/fqsafe/fyy001.

[fsn370553-bib-0294] Taulescu, C. , M. Mihaiu , C. Bele , et al. 2010. “Manipulating the Fatty Acid Composition of Poultry Meat for Improving Consumer's Health.” Bulletin of the University of Agricultural Sciences and Veterinary Medicine 67: 220–225. 10.15835/buasvmcn-vm:67:2:6034.

[fsn370553-bib-0207] Terra, W. R. , and C. Ferreira . 2020. “Evolutionary Trends of Digestion and Absorption in the Major Insect Orders.” Arthropod Structure and Development 56: 100931. 10.1016/j.asd.2020.100931. 32203883

[fsn370553-bib-0209] Toprak, U. , and L. P. Musselman . 2021. “From Cellular Biochemistry to Systems Physiology: New Insights Into Insect Lipid Metabolism.” Insect Biochemistry and Molecular Biology 133: 103585. 10.1016/j.ibmb.2021.103585. 33915290

[fsn370553-bib-0208] Toprak, U. , D. Hegedus , C. Doğan , and G. Güney . 2020. “A Journey Into the World of Insect Lipid Metabolism.” Archives of Insect Biochemistry and Physiology 2020: e21682. 10.1002/arch.21682. 32335968

[fsn370553-bib-0306] Twining, C. W. , P. Lawrence , D. W. Winkler , A. S. Flecker , and J. T. Brenna . 2018. “Brenna Conversion Efficiency of α‐Linolenic Acid to Omega‐3 Highly Unsaturated Fatty Acids in Aerial Insectivore Chicks.” Journal of Experimental Biology 221, no. 3: jeb165373. 10.1242/jeb.165373. 29217628

[fsn370553-bib-0214] Tzompa‐Sosa, D. A. , and V. Fogliano . 2017. “Potential of Insect‐Derived Ingredients for Food Applications.” In Insect Physiology and Ecology. IntechOpen. 10.5772/67318.

[fsn370553-bib-0213] Tzompa‐Sosa, D. A. , k. Dewettinck , P. Provijn , J. F. Brouwers , B. de Meulenaer , and D. G. A. B. Oonincx . 2021. “Lipidome of Cricket Species Used as Food.” Food Chemistry 349: 129077. 10.1016/j.foodchem.2021.129077. 33571921

[fsn370553-bib-0211] Tzompa‐Sosa, D. A. , K. Dewettinck , X. Gellynck , and J. J. Schouteten . 2021. “Replacing Vegetable Oil by Insect Oil in Food Products: Effect of Deodorization on the Sensory Evaluation.” Food Research International 141: 110140. 33642007 10.1016/j.foodres.2021.110140

[fsn370553-bib-0212] Tzompa‐Sosa, D. A. , K. Dewettinck , X. Gellynck , and J. J. Schouteten . 2022. “Consumer Acceptance Towards Potato Chips Fried in Yellow Mealworm Oil.” Food Quality and Preference 97: 104487. 10.1016/j.foodqual.2021.104487.

[fsn370553-bib-0216] Tzompa‐Sosa, D. A. , L. Yi , H. J. F. van Valenberg , and C. M. M. Lakemond . 2019. “Four Insect Oils as Food Ingredient: Physical and Chemical Characterisation of Insect Oils Obtained by an Aqueous Oil Extraction.” Journal of Insects as Food and Feed 5, no. 4: 279–292. 10.3920/JIFF2018.0020.

[fsn370553-bib-0217] Tzompa‐Sosa, D. A. , L. Yi , H. J. F. van Valenberg , M. A. J. S. van Boekel , and C. M. M. Lakemond . 2014. “Insect Lipid Profile: Aqueous Versus Organic Solvent‐Based Extraction Methods.” Food Research International 62: 1087–1094. 10.1016/j.foodres.2014.05.052.

[fsn370553-bib-0215] Tzompa‐Sosa, D. A. , P. Provijn , X. Gellynck , and J. J. Schouteten . 2023. “Frying Dough With Yellow Mealworm Oil: Aroma Profile and Consumer Perception at a Central Location Test and at Home.” Journal of Food Science 88, no. S1: A130–A146. 10.1111/1750-3841.16389. 36478571

[fsn370553-bib-0260] Udomsil, N. , S. Imsoonthornruksa , C. Gosalawit , and M. Ketudat‐Cairns . 2019. “Nutritional Values and Functional Properties of House Cricket (*Acheta domesticus*) and Field Cricket (*Gryllus bimaculatus*).” Food Science and Technology Research 25, no. 4: 597–605. 10.3136/fstr.25.597.

[fsn370553-bib-0297] Ushakova, N. A. , E. S. Brodskii , A. A. Kovalenko , A. I. Bastrakov , A. A. Kozlova , and D. S. Pavlov . 2016. “Characteristics of Lipid Fractions of Larvae of the Black Soldier Fly *Hermetia illucens* .” Doklady. Biochemistry and Biophysics 468: 209–212. 10.1134/S1607672916030145. 27417723

[fsn370553-bib-0218] Van De Bor, V. , V. Loreau , M. Malbouyres , et al. 2021. “A Dynamic and Mosaic Basement Membrane Controls Cell Intercalation in Drosophila Ovaries.” Development 148: dev195511. 10.1242/dev.195511. 33526583

[fsn370553-bib-0299] van Heugten, E. , G. Martinez , A. McComb , and E. Koutsos . 2019. “285 Black Soldier fly (*Hermetia illucens*) Larvae Oil Improves Growth Performance of Nursery Pigs.” Journal of Animal Science 97, no. Supplement_3: 118. 10.1093/jas/skz258.244. PMC9736315 36496772

[fsn370553-bib-0219] Van Huis, A. 2013. “Potential of Insects as Food and Feed in Assuring Food Security.” Annual Review of Entomology 58, no. 1: 563–583. 10.1146/annurev-ento-120811-153704. 23020616

[fsn370553-bib-0220] van Huis, A. 2014. The Global Impact of Insects. Wageningen University.

[fsn370553-bib-0221] van Huis, A. , and B. Rumpold . 2023. “Strategies to Convince Consumers to Eat Insects? A Review.” Food Quality and Preference 110: 104927. 10.1016/j.foodqual.2023.104927.

[fsn370553-bib-0222] van Huis, A. , and J. K. Tomberlin , eds. 2017. Insects as Food and Feed: From Production to Consumption. Wageningen Academic Publishers. 10.3920/978-90-8686-849-0.

[fsn370553-bib-0223] Veldkamp, T. , G. Van Duinkerken , A. Van Huis , et al. 2012. Insects as a Sustainable Feed Ingredient in Pig and Poultry Diets: A Feasibility Study. Wageningen UR Livestock Research.

[fsn370553-bib-0224] Velíšek, J. 2014. The Chemistry of Food. 1st ed, 348. John Wiley & Sons, Ltd.

[fsn370553-bib-0225] Verheyen, G. R. , T. Ooms , L. Vogels , et al. 2018. “Insects as an Alternative Source for the Production of Fats for Cosmetics.” Journal of Cosmetic Science 69, no. 3: 187–202. 30052193

[fsn370553-bib-0226] Waitha, M. K. , k. M. Osuga , L. W. Kabuage , et al. 2022. “Evaluating the Growth and Cost–Benefit Analysis of Feeding Improved Indigenous Chicken With Diets Containing Black Soldier Fly Larva Meal.” Frontiers in Insect Science 2: 933571. 10.3389/finsc.2022.933571. 38468810 PMC10926451

[fsn370553-bib-0268] Wang, Y. , D. S. Lin , L. Bolewicz , and W. E. Connor . 2006. “The Predominance of Polyunsaturated Fatty Acids in the Butterfly *Morpho peleides* Before and After Metamorphosis.” Journal of Lipid Research 47, no. 3: 530–536. 10.1194/jlr.M500346-JLR200. 16322637

[fsn370553-bib-0280] Wang, H. , K. U. Rehman , X. Liu , et al. 2017. “Insect Biorefinery: A Green Approach for Conversion of Crop Residues Into Biodiesel and Protein.” Biotechnology for Biofuels 10: 304. 10.1186/s13068-017-0986-7. 29255487 PMC5729465

[fsn370553-bib-0227] Wang, J. , X. Wang , J. Li , Y. Chen , W. Yang , and L. Zhang . 2015. “Effects of Dietary Coconut Oil as a Medium‐Chain Fatty Acid Source on Performance, Carcass Composition and Serum Lipids in Male Broilers.” Asian‐Australasian Journal of Animal Sciences 28: 223–230. 25557818 10.5713/ajas.14.0328 PMC4283167

[fsn370553-bib-0228] Wang, Y.‐S. , and M. Shelomi . 2017. “Review of Black Soldier Fly (*Hermetia illucens*) as Animal Feed and Human Food.” Food 6: 91. 10.3390/foods6100091 PMC5664030 29057841

[fsn370553-bib-0229] Williams, J. P. , J. R. Williams , A. Kirabo , D. Chester , and M. Peterson . 2016. “Nutrient Content and Health Benefits of Insects.” In Insects as Sustainable Food Ingredients, 61–84. Elsevier Inc. 10.1016/B978-0-12-802856-8.00003-X.

[fsn370553-bib-0269] Womeni, H. M. , B. Tiencheu , F. T. Mbiapo , et al. 2009. “Oils of Insects and Larvae Consumed in Africa: Potential Sources of Polyunsaturated Fatty Acids.” Oléagineux, Corps Gras, Lipides 16: 230–235.

[fsn370553-bib-0277] Wong, C.‐Y. , S.‐S. Rosli , Y. Uemura , et al. 2019. “Potential Protein and Biodiesel Sources From Black Soldier Fly Larvae: Insights of Larval Harvesting Instar and Fermented Feeding Medium.” Energies 12: 1570. 10.3390/en12081570.

[fsn370553-bib-0231] Wrońska, A. K. , A. Kaczmarek , M. I. Boguś , and A. Kuna . 2023. “Lipids as a Key Element of Insect Defense Systems.” Frontiers in Genetics 14: 1183659. 10.3389/fgene.2023.1183659. 37359377 PMC10289264

[fsn370553-bib-0230] Wrońska, A. K. , M. I. Boguś , E. Włóka , M. Kazek , A. Kaczmarek , and K. Zalewska . 2018. “Cuticular Fatty Acids of *Galleria Mellonella* (Lepidoptera) Inhibit Fungal Enzymatic Activities of Pathogenic *Conidiobolus Coronatus* .” PLoS One 13, no. 3: e0192715. 10.1371/journal.pone.0192715. 29518079 PMC5843172

[fsn370553-bib-0257] Wu, R. A. , Q. Ding , L. Yin , et al. 2020. “Comparison of the Nutritional Value of Mysore Thorn Borer (*Anoplophora chinensis*) and Mealworm Larva (*Tenebrio molitor*): Amino Acid, Fatty Acid, and Element Profiles.” Food Chemistry 323: Article 126818. 10.1016/j.foodchem.2020.126818. 32330649

[fsn370553-bib-0232] Wu, Z. , J. Soulages , B. Joshi , S. Daniel , Z. Hager , and E. Arrese . 2014. “TGL‐Mediated Lipolysis in *Manduca sexta* Fat Body: Possible Roles for Lipoamide‐Dehydrogenase (LipDH) and High‐Density Lipophorin (HDLp).” Insect Biochemistry and Molecular Biology 45, no. 1: 58–68. 10.1016/j.ibmb.2013.12.001. 24333838 PMC3932539

[fsn370553-bib-0233] Yan, X. , M. Federighi , J. M. Cappelier , V. Jury , and G. Boué . 2024. “Consumer Acceptance of Different Insect‐Based Foods: A Cross‐Cultural Study in China and France.” Journal of Insects as Food and Feed 11, no. 4: 621–639. 10.1163/23524588-00001090.

[fsn370553-bib-0266] Yang, L.‐F. , S. Siriamornpun , and D. Li . 2006. “Polyunsaturated Fatty Acid Content of Edible Insects in Thailand.” Journal of Food Lipids 13: 277–285. 10.1111/j.1745-4522.2006.00051.x.

[fsn370553-bib-0234] Yeo, H. , K. Youn , M. Kim , et al. 2013. “Fatty Acid Composition and Volatile Constituents of Protaetia Brevitarsis Larvae.” Preventive Nutrition and Food Science 18, no. 2: 150–156. 10.3746/pnf.2013.18.2.150. 24471125 PMC3892504

[fsn370553-bib-0235] Yi, L. , C. M. Lakemond , L. M. Sagis , V. Eisner‐Schadler , A. van Huis , and M. A. Boekel . 2013. “Extraction and Characterisation of Protein Fractions From Five Insect Species.” Food Chemistry 141, no. 4: 3341–3348. 10.1016/j.foodchem.2013.05.115. 23993491

[fsn370553-bib-0304] Yi, L. Y. 2015. A Study on the Potential of Insect Protein and Lipid as a Food Source. Wageningen University.

[fsn370553-bib-0298] Zeitz, J. O. , J. Fennhoff , H. Kluge , G. I. Stangl , and K. Eder . 2015. “Effects of Dietary Fats Rich in Lauric and Myristic Acid on Performance, Intestinal Morphology, Gut Microbes, and Meat Quality in Broilers.” Poultry Science 94, no. 10: 2404–2413. 10.3382/ps/pev191. 26240391

[fsn370553-bib-0274] Zhang, C. , D. Wei , G. Shi , et al. 2019. “Understanding the Regulation of Overwintering Diapause Molecular Mechanisms in *Culex pipiens pallens* Through Comparative Proteomics.” Scientific Reports 9, no. 1: 6485. 10.1038/s41598-019-42961-w. 31019237 PMC6482188

[fsn370553-bib-0237] Zhang, J. B. , J. Zhang , J. H. Li , et al. 2021. “Black Soldier Fly: A New Vista for Livestock and Poultry Manure Management.” Journal of Integrative Agriculture 20, no. 5: 1167–1179. 10.1016/S2095-3119(20)63423-2.

[fsn370553-bib-0238] Zhao, M. , C.‐Y. Wang , L. Sun , et al. 2021. “Edible Aquatic Insects: Diversities, Nutrition, and Safety.” Food 10: 3033. 10.3390/foods10123033. PMC8700862 34945584

[fsn370553-bib-0239] Zhao, X. , J. L. Vázquez‐Gutiérrez , D. P. Johansson , R. Landberg , and M. Langton . 2016. “Yellow Mealworm Protein for Food Purposes ‐ Extraction and Functional Properties.” PLoS One 11: 0147791. 10.1371/journal.pone.0147791. PMC4739691 26840533

[fsn370553-bib-0241] Zheng, L. , Q. Li , J. Zhang , and Z. Yu . 2012. “Double the Biodiesel Yield: Rearing Black Soldier Fly Larvae, *Hermetia illucens* , on Solid Residual Fraction of Restaurant Waste After Grease Extraction for Biodiesel Production.” Renewable Energy 41: 75–79.

[fsn370553-bib-0240] Zheng, L. , Y. Hou , W. Li , S. Yang , Q. Li , and Z. Yu . 2013. “Exploring the Potential of Grease From Yellow Mealworm Beetle ( *Tenebrio molitor* ) as a Novel Biodiesel Feedstock.” Applied Energy 101: 618–621.

[fsn370553-bib-0242] Zielińska, E. , B. Baraniak , M. Karaś , K. Rybczyńska , and A. Jakubczyk . 2015. “Selected Species of Edible Insects as a Source of Nutrient Composition.” Food Research International 77: 460–466. 10.1016/j.foodres.2015.09.008.

[fsn370553-bib-0243] Żuk‐Gołaszewska, K. , R. Gałęcki , K. Obremski , S. Smetana , S. Figiel , and J. Gołaszewski . 2022. “Edible Insect Farming in the Context of the EU Regulations and Marketing—An Overview.” Insects 13, no. 5: 446. 10.3390/insects13050446. 35621781 PMC9147295

